# Implementation of a Secure Firearm Storage Program in Pediatric Primary Care: A Cluster Randomized Trial

**DOI:** 10.1001/jamapediatrics.2024.3274

**Published:** 2024-09-03

**Authors:** Rinad S. Beidas, Kristin A. Linn, Jennifer M. Boggs, Steven C. Marcus, Katelin Hoskins, Shari Jager-Hyman, Christina Johnson, Melissa Maye, LeeAnn Quintana, Courtney Benjamin Wolk, Leslie Wright, Celeste Pappas, Arne Beck, Katy Bedjeti, Alison M. Buttenheim, Matthew F. Daley, Marisa Elias, Jason Lyons, Melissa Lynne Martin, Bridget McArdle, Debra P. Ritzwoller, Dylan S. Small, Nathaniel J. Williams, Shiling Zhang, Brian K. Ahmedani

**Affiliations:** 1 Department of Medical Social Sciences, Northwestern University Feinberg School of Medicine, Chicago, Illinois; 2 Department of Biostatistics, Epidemiology, & Informatics, University of Pennsylvania Perelman School of Medicine, Philadelphia; 3 Kaiser Permanente Colorado Institute for Health Research, Aurora; 4 University of Pennsylvania School of Social Policy and Practice, Philadelphia; 5 Department of Biobehavioral Health Sciences, University of Pennsylvania School of Nursing, Philadelphia; 6 Leonard Davis Institute of Health Economics, University of Pennsylvania, Philadelphia; 7 Department of Psychiatry, University of Pennsylvania Perelman School of Medicine, Philadelphia; 8 Henry Ford Health, Center for Health Policy & Health Services Research, Detroit, Michigan; 9 Department of Family and Community Health, University of Pennsylvania School of Nursing, Philadelphia; 10 Department of Pediatrics, Henry Ford Health, Royal Oak, Michigan; 11 Henry Ford Health, Sterling Heights, Michigan; 12 Department of Statistics and Data Science, the Wharton School, University of Pennsylvania, Philadelphia; 13 Boise State University School of Social Work, Boise, Idaho

## Abstract

**Importance:**

Increased secure firearm storage can reduce youth firearm injury and mortality, a leading cause of death for children and adolescents in the US. Despite the availability of evidence-based secure firearm storage programs and recommendations from the American Academy of Pediatrics, few pediatric clinicians report routinely implementing these programs.

**Objective:**

To compare the effectiveness of an electronic health record (EHR) documentation template (nudge) and the nudge plus facilitation (ie, clinic support to implement the program; nudge+) at promoting delivery of a brief evidence-based secure firearm storage program (SAFE Firearm) that includes counseling about secure firearm storage and free cable locks during all pediatric well visits.

**Design, Setting, and Participants:**

The Adolescent and Child Suicide Prevention in Routine Clinical Encounters (ASPIRE) unblinded parallel cluster randomized effectiveness-implementation trial was conducted from March 14, 2022, to March 20, 2023, to test the hypothesis that, relative to nudge, nudge+ would result in delivery of the firearm storage program to an additional 10% or more of the eligible population, and that this difference would be statistically significant. Thirty pediatric primary care clinics in 2 US health care systems (in Michigan and Colorado) were included, excluding clinics that were not the primary site for participating health care professionals and a subset selected at random due to resource limitations. All pediatric well visits at participating clinics for youth ages 5 to 17 years were analyzed.

**Interventions:**

Clinics were randomly assigned in a 1:1 ratio to receive either the nudge or nudge+.

**Main Outcomes and Measures:**

Patient-level outcomes were modeled to estimate the primary outcome, reach, which is a visit-level binary indicator of whether the parent received both components of the firearm storage program (counseling and lock), as documented by the clinician in the EHR. Secondary outcomes explored individual program component delivery.

**Results:**

A total of 47 307 well-child visits (median [IQR] age, 11.3 [8.1-14.4] years; 24 210 [51.2%] male and 23 091 [48.8%] female) among 46 597 children and 368 clinicians were eligible to receive the firearm storage program during the trial and were included in analyses. Using the intention-to-treat principle, a higher percentage of well-child visits received the firearm storage program in the nudge+ condition (49%; 95% CI, 37-61) compared to nudge (22%; 95% CI, 13-31).

**Conclusions and Relevance:**

In this study, the EHR strategy combined with facilitation (nudge+) was more effective at increasing delivery of an evidence-based secure firearm storage program compared to nudge alone.

**Trial Registration:**

ClinicalTrials.gov Identifier: NCT04844021

## Key Points

**Question:**

Will an electronic health record (EHR) strategy coupled with facilitation (nudge+) perform better than an EHR strategy alone (nudge) at increasing clinician-reported delivery of an evidence-based secure firearm storage program (SAFE Firearm) during pediatric well visits?

**Findings:**

In this cluster randomized trial among 47 307 well-child visits at 30 clinics in Michigan and Colorado, the chance of receiving the firearm storage program was significantly higher in clinics in the nudge+ group than in the nudge group.

**Meaning:**

The findings suggest that an EHR strategy combined with facilitation may be more effective at increasing delivery of an evidence-based firearm storage program in pediatric primary care than an EHR strategy alone.

## Teaser

This cluster randomized trial evaluates the use of an electronic health record documentation template alone vs with facilitation support for promoting delivery of a secure firearm storage program.

## Introduction

Firearms are a leading cause of death for young people.^1^ Firearm suicides make up approximately half of youth suicides, contributed to a cumulative loss of 1.3 million years of life from 2013 to 2020,^2^ and comprise more than one-third of all youth firearm deaths.^3,4^ In 2021, approximately 4.6 million children in the US lived in households with a loaded and unlocked firearm.^5^ Deploying secure firearm storage programs with parents to reduce children’s unauthorized access to firearms is a promising yet underused strategy that can reduce firearm mortality and suicide, given that most youth firearm suicides involve a family member’s firearm.^6^ A modest increase in secure firearm storage could prevent up to 32% of youth firearm suicide and unintentional injury deaths.^7^

Evidence-based secure firearm storage programs include brief firearm safety counseling between a pediatric primary care clinician and parent, plus locking device distribution.^8^ An American Academy of Pediatrics policy statement recommends the role that pediatric clinicians can play in firearm safety-related anticipatory guidance.^9,10^ However, few pediatric clinicians report routinely counseling parents on secure firearm storage (28%) or offering locks (2%) during well-child visits,^11^ representing a research-to-practice gap. The 2024 Surgeon General advisory explicitly calls for implementation research focused on closing this gap in firearm injury and mortality.^12^

The primary aim of the Adolescent and Child Suicide Prevention in Routine Clinical Encounters (ASPIRE) trial was to test 2 strategies to implement a brief evidence-based secure firearm storage program, SAFE Firearm,^13,14,15^ in pediatric primary care. This trial builds on preimplementation work^13,14,15^ and explicitly targets barriers relevant to the use of secure firearm storage programs.^16^ We conducted a parallel cluster randomized effectiveness-implementation trial in which 30 pediatric primary care clinics within 2 health care systems were randomly assigned to receive either a strategy using the electronic health record (EHR; nudge), which included integration of a new EHR documentation template into the standard well-child visit workflow, or the EHR strategy combined with facilitation (ie, clinic-level support to embed the program; nudge+). We examined the hypothesis that the EHR strategy coupled with facilitation would perform significantly better than the EHR strategy alone with a 10% superiority margin.

## Methods

### Study Design, Population, and Setting

The ASPIRE trial was a 1-year unblinded parallel cluster randomized effectiveness-implementation trial conducted within 30 clinics that are part of 2 large and geographically, demographically, and culturally diverse health care systems that are members of the Mental Health Research Network, a consortium of 14 embedded research centers dedicated to improving patient mental health through research, practice, and policy. The health care systems provide comprehensive medical and behavioral health care to urban, suburban, and rural communities in Michigan and Colorado, respectively, and the demographic characteristics of patient populations served across the 2 systems are diverse in age, ethnicity, and race. The trial protocol (Supplement 1) was published previously^17^ and was approved by the independent data safety and monitoring board that oversaw the study and the University of Pennsylvania single institutional review board. Informed consent and Health Insurance Portability and Accountability Act (HIPAA) authorization for the firearm storage program delivery (considered standard of care) and EHR data extraction were waived by the institutional review board because the research was considered no more than minimal risk and would not adversely impact the rights and welfare of participants; moreover, consent for every health care professional and parent was not considered practicable.

**Figure 1.**
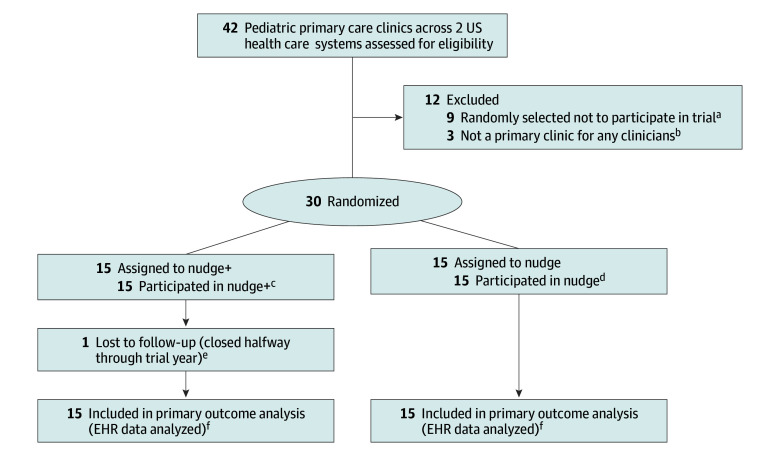
Adolescent and Child Suicide Prevention in Routine Clinical Encounters (ASPIRE) Trial Clinic Screening, Randomization, and Follow-Up The figure shows selection and randomization of clinics from 2 health care systems, 1 in Michigan and 1 in Colorado. ^a^The 3 clinics that were excluded from the trial because they were not primary locations where services were provided for any clinicians still received the electronic health record (EHR) strategy (ie, nudge) as a quality improvement initiative but were not included in our analyses. ^b^Resources were only available to collect all study outcomes from 18 clinics in the Colorado health care system, so a random subset of 9 eligible Colorado site clinics was selected to not participate in data collection. These clinics still received the electronic health record (EHR) strategy (ie, nudge) as a quality improvement initiative but were not included in our analyses. ^c^Median (range) well-child visits per clinic during trial year, 1212 (73-4840). ^d^Median (range) well-child visits per clinic during trial year, 1008 (301-3523). ^e^The clinic in the nudge plus facilitation (nudge+, the EHR strategy plus facilitation support) condition that closed during the trial had their data included in analyses for the period that the clinic was open during the trial. ^f^Visit data were extracted from the EHR from all participating clinics using the following criteria: (1) the well-child visit was completed at a participating study clinic from March 14, 2022, to March 13, 2023 (Michigan), or March 21, 2022, to March 20, 2023 (Colorado); (2) the visit was conducted in person; (3) the department assigned to the visit was pediatrics (both systems) or primary care (Colorado only); (4) the health care professional for the visit was a clinician within the health care system’s medical group (Michigan only); (5) the health care professional for the visit had a specialty of pediatrics or had a specialty of primary care with 5% or more of their patient panel being pediatric patients (Colorado only); (6) the health care professional for the visit was a medical doctor, doctor of osteopathy, physician assistant, nurse practitioner, registered nurse, or resource clinician (ie, a clinician filling in at a clinic temporarily); (7) the patient was not on the health care system’s research exclusion list; and (8) the patient’s age was between 5.0 and 17.9 years at the time of the visit.

Participants included parents or legal guardians (hereafter, parents) at participating pediatric clinics who attended an in-person well-child visit with their child aged 5 to 17 years from March 14, 2022, until March 20, 2023, and pediatric physicians and nonphysician clinicians (eg, advanced practice nurses and physician assistants) delivering well-child visit care through pediatrics or family medicine within the 2 systems (hereafter, clinicians). The Consolidated Standards of Reporting Trials (CONSORT) reporting guideline was followed in reporting this study (Figure 1).

### Randomization

Clinics were randomly assigned to either nudge or nudge+ using covariate-constrained randomization^18^ to achieve balance with respect to 3 practice-level covariates. These included health care system, clinic size, and whether a clinician partner (ie, someone involved in study planning and advising) worked at the clinic.

### Program

Prior to this trial, we adapted an evidence-based secure firearm storage program (Safety Check^15^) using a systematic approach informed by constituent input.^13,14^ The adapted firearm storage program is a universal approach that includes brief counseling (<1 minute) between the pediatric clinician and parent(s) about secure firearm storage and offering free cable locks (Table 1^19^). The firearm storage program does not include firearm ownership screening or documentation in the EHR^20^ to increase acceptance among firearm-owning families.^21^ The focus on maximizing secure storage rather than curtailing firearm ownership is consistent with a harm-reduction approach.^9,22^ Harm reduction embraces autonomy and pragmatism, supporting incremental steps toward more secure storage with the ultimate goal of reducing firearm accessibility to unauthorized people.^23^ Program materials are available on request.^24^

**Table 1.** Firearm Storage Program and Implementation Strategy Components Delivered in Each Study Condition

Component and approach	Actor	Specific action	Recipient	Condition	
				Nudge	Nudge+
Firearm storage program					
Brief counseling about secure firearm storage	Clinician	Clinician engaging parents in a <1-min discussion about secure firearm storage as part of anticipatory guidance related to safety during the pediatric well visit.	Parents	Yes	Yes
Offering free cable locks	Clinician	Clinician offering free cable locks to parents during the pediatric well visit.	Parents	Yes	Yes
Collateral documents related to secure storage (handouts, link in after visit summary, posters)	Clinic	Clinic making handouts available to parents with more information about secure firearm storage; adding a link to website containing additional resources about secure firearm storage to the after visit summary; hanging posters on wall within clinics to create a culture that signals secure firearm storage is important.	Parents	Yes	Yes
Implementation strategies					
Training	Research team^a^	Brief training prior to trial launch during pediatrics department meeting regarding program delivery, electronic health record documentation, and collateral documents. After the training, clinicians were also encouraged to access brief online training videos from the American Academy of Pediatrics^19^ that simulate secure firearm storage counseling scenarios and offered continuing medical education credit.	Clinicians	Yes	Yes
Identifying clinician partners	Research team^a^	Clinician partners within the pediatric department at each health care system who advised on study design and delivery of the program across clinics.	Clinics	Yes	Yes
Electronic health record prompt	Research team^a^	Information technology specialists and the research team developed (in collaboration with clinician partners at the health care systems) and deployed a SmartList to add to the well-child visit template; it included prompts related to both program components (counseling and locks). SmartLists are predefined lists of choices that users can select, which are helpful for documenting values that a clinician is required to use repeatedly. The prompt was refined based on behavioral economic theory and pilot tested prior to trial launch.	Health care system	Yes	Yes
Facilitation^b^	Research team^a^	Clinic-level implementation strategy that included many potential activities and could be tailored to the individual clinic’s needs and requests for assistance. Components that were delivered most commonly during the present study are listed in the following rows. The overall goal of the facilitator was to engage with study clinics, assist each clinic in setting goals around the implementation of SAFE Firearm, and troubleshoot barriers to implementation. Facilitators, who were health care system employees and members of the research team but not employees of study clinics, attended the US Department of Veterans Affairs Quality Enhancement Research Initiative facilitation training and met regularly with each other and 2 psychologists with expertise in facilitation.	Clinicians, clinic change agents, clinics^c^	No	Yes
Identification/selection of local change agents	Research team^a^	Identify and prepare individuals within each clinic who dedicate themselves to supporting, marketing, and driving through the implementation, and overcoming indifference or resistance to SAFE Firearm in the clinic.	Clinicians, clinic change agents^c^	No	Yes
Engaging local change agents, obtaining buy-in	Research team^a^	Engage or include relevant constituents in the implementation effort, cultivate relationships, generate enthusiasm.	Clinicians, clinic change agents^c^	No	Yes
Development of implementation plan	Research team^a^	Develop a formal implementation blueprint that includes all goals and strategies. The blueprint included the aim/purpose of the implementation, the scope of the change (eg, what organizational units are affected), timeframe and milestones, and appropriate performance/progress measures.	Clinicians, clinic change agents^c^	No	Yes
Ongoing action/implementation planning	Research team^a^	Use and update the implementation plan to guide the implementation effort over time.	Clinicians, clinic change agents^c^	No	Yes
Providing updates	Research team^a^	Share information about the project or share information about the health care system that is relevant to the implementation.	Clinicians, clinic change agents^c^	No	Yes
Ongoing consultation	Research team^a^	Provide ongoing consultation with ≥1 experts in the strategies used to support implementing SAFE Firearm (ie, offer expertise, answer questions, guide practice).	Clinicians, clinic change agents^c^	No	Yes
Problem solving	Research team^a^	Engage in a collaborative process with clinics to generate potential solutions to identified problems and support them in choosing and developing a plan to implement ≥1 of the identified solutions.	Clinicians, clinic change agents^c^	No	Yes
Consulting with content expert	Research team^a^	Consult with firearm safety expert.	Clinicians, clinic change agents^c^	No	Yes
Problem identification and goal setting	Research team^a^	Engage in a collaborative process with clinics to identify implementation problems or goals.	Clinicians, clinic change agents^c^	No	Yes
Data collection to assess context/baseline performance	Research team^a^	Collect and summarize clinical performance data over a specified period.	Clinicians, clinic change agents^c^	No	Yes
Providing feedback	Research team^a^	Share clinical performance data or impressions with clinicians and administrators to support clinician implementation.	Clinicians, clinic change agents^c^	No	Yes
Transitioning from active facilitation to self-maintenance	Research team^a^	Support transitioning the clinic from implementation with support from a facilitator to independence.	Clinicians, clinic change agents^c^	No	Yes
Administrative tasks	Research team^a^	Includes scheduling meetings and documentation of facilitation activities.	Clinicians, clinic change agents^c^	No	Yes

^a^ Research team included external researchers and internal researchers embedded within the health care systems. Facilitators were part of the research team and were health care system employees not affiliated with the pediatric clinics.

^b^ Approximately 6% of facilitation was delivered via in-person interactions, with the rest delivered via asynchronous and synchronous communication including email and chat.

^c^ Clinic change agents were administrative or clinical staff identified at the beginning of facilitation to serve as the point of contact for the facilitator.

### Study Conditions

Study conditions were (1) nudge, the integration of a new EHR documentation template into the standard well-child visit workflow, and (2) nudge+, the nudge protocol combined with facilitation. For the nudge condition, we worked with health care system leadership and information technology to develop, pilot test, and deploy a new EHR documentation template within the standard well-child visit workflow to serve as a reminder and allow for tracking of the firearm storage program delivery. Specifically, we used a SmartList approach in each health care system’s instance of the Epic EHR (Epic Systems). SmartLists are predefined lists of choices that are helpful for documenting values within progress notes that clinicians use repeatedly, thus improving efficiency. The SmartList was incorporated into the standard template used by all clinicians for a well-child visit for children aged 5 to 17 years. We designed the nudge strategy based on insights from behavioral economics^25,26,27^ (eg, requiring clinicians to make an active choice regarding the firearm storage program delivery). During a routine well-child visit, the clinician keystrokes through the required prompts and selects responses from a drop-down list (eg, respond yes or no separately for each: “secure firearm storage discussed” and “cable lock offered”) (eFigure 1 in Supplement 2). While we asked clinicians to complete it at each visit, clinicians sometimes left it blank (ie, skipped) which was treated as nondelivery.

For the nudge+ condition, the nudge strategy was combined with facilitation provided by health care system employees—who were members of the research team and not affiliated with the pediatric primary care clinics—to provide clinic-level implementation support. Facilitation was offered for 12 months to each clinic, in keeping with other trials.^28,29,30,31,32^ Facilitators, trained through the US Department of Veterans Affairs Quality Enhancement Research Initiative facilitation program, spent on average 8.7 hours per clinic over the trial year (approximately 6% via in-person interactions, with the rest delivered via asynchronous and synchronous communication, including email and chat) delivering these supports. See Table 1 for a detailed description of conditions.

### Outcomes

All data used in our analyses, including reach and patient-reported demographic characteristics (eg, age, race, ethnicity, and sex), were extracted from the EHR. Reporting race and ethnicity in this study was mandated by the US National Institutes of Health. Additionally, we used race and ethnicity to characterize the representativeness of the study population, generalizability of the results, and provide information about potential disparities and inequities. Response options for patient-reported demographic characteristics were set by the health care systems. The primary outcome, reach, was a visit-level binary indicator of whether the parent received both components of the firearm storage program (counseling and lock), as documented by the clinician in the EHR. Secondary outcomes explored whether the parent received each individual program component (ie, separate binary indicators for counseling and lock).

### Statistical Analysis

We powered our study based on the availability of 32 clinics to randomize (18 in the Colorado system and 14 in the Michigan system). We determined 32 clinics would provide more than 90% power to detect a difference of 10% or more in the rate of program delivery in nudge+, assuming a type I error of 0.05, intracluster correlation of 0.03 within clinic, and 30% reach in nudge.

In the months preceding randomization and trial launch, we evaluated all clinics for eligibility and identified that 12 were eligible in Michigan and 27 in Colorado. Due to resource constraints, we were unable to add more trial clinics at the Colorado site to reach 32 total clinics across the 2 systems. Thus, we only randomized and included 30 clinics in the trial (12 in Michigan and 18 in Colorado, with a random subset of Colorado clinics selected for exclusion).

We first calculated standardized mean differences (SMDs) to assess whether the clinic-level randomization sufficiently balanced clinic- and patient-level variables across study conditions. The primary analysis involved fitting generalized estimating equations with a binomial distribution and logit link^33,34^ to estimate reach for nudge and nudge+ along with the risk difference between conditions. In all models, we specified an exchangeable working correlation structure at the clinic level and used the delta method to generate confidence intervals for the risk difference. We fit adjusted generalized estimating equation models that included terms for the variables used to constrain the clinic-level randomization (health care system, clinic size, and clinician partner at clinic), variables prespecified based on results from our team’s preimplementation pilot (patient sex),^35^ and variables with an absolute SMD value that exceeded 0.2. We used Kauermann and Carroll^36^ small cluster correction in all generalized estimating equation analyses. All analyses were conducted in Stata version 18 (StataCorp), using the xtgeebcv^37^ procedure and the margins command (K.A.L. and K.B.).

We used a multiple hypothesis testing framework to evaluate the effectiveness of nudge+ compared to nudge,^38^ considering 3 scenarios: (1) nudge+ is more effective than nudge by more than 10 percentage points (PP); (2) nudge+ is as effective as nudge, with a difference of no more than 10 percentage points in either direction; and (3) nudge+ is less effective than nudge by more than 10 percentage points. The margin of 10 percentage points (0.1 on the risk difference scale) was selected based on discussions with health care system leaders and previous studies to ensure the differences were clinically meaningful.^39,40,41,42^ We treated each scenario as a separate hypothesis to be tested at a 5% significance level for the primary outcome, reach. Since these scenarios are mutually exclusive, we did not need to adjust for testing multiple hypotheses.^38^ If the 95% CI for the risk difference for reach did not contain any values within a region (nudge+ superior, equivalence, or nudge+ inferior), that region could be rejected. For the secondary outcomes (counseling and lock), we applied a Bonferroni correction to account for multiplicity, constructing 97.5% CIs to test the 3 scenarios at a 2.5% significance level. Two-sided *P* values less than .05 were considered statistically significant for the primary outcome. Two-sided *P* values less than .025 were considered statistically significant for the secondary outcomes. See Supplement 1 for a detailed statistical analysis plan.

## Results

### Study Population

Data from 47 307 well-child visits (median [IQR] age, 11.3 [8.1-14.4] years; 24 210 [51.2%] male and 23 091 [48.8%] female) among 46 597 children and 368 clinicians and across 30 pediatric primary care clinics were included in the final analytic sample (24 989 in nudge and 22 318 in nudge+). Table 2^43^ presents clinic- and patient-level summaries by condition and the SMD of each variable. Hispanic ethnicity (SMD, 0.22) and Black race (SMD, −0.31) were imbalanced across arms. We included Hispanic ethnicity as a covariate in adjusted models but not race because models that included both health system and race did not converge.

**Table 2.** Clinic- and Patient-Level Characteristics by Condition

Characteristic	Unadjusted No. (%)		SMD^a^
	Nudge+ (n = 15)	Nudge (n = 15)	
Clinic level			
Clinic size, mean (SD), No. of patient visits^b^	1550 (1158)	1681 (1295)	−0.19
Clinician partner at clinic^c^	4 (27)	3 (20)	0.35
Proportion rural, mean (SD)^d^	0.003 (0.005)	0.014 (0.048)	−0.14
Patient level			
No.	22 318	24 989	NA
Patient age, mean (SD), y	11.4 (3.7)	11.1 (3.7)	0.09
Sex			
Male	11 367 (51)	12 843 (51)	−0.01
Female	10 948 (49)	12 143 (49)	0.01
Missing	3 (<0.1)	3 (<0.1)	NA
Ethnicity^e^			
Hispanic	3879 (17)	2459 (10)	0.22
Non-Hispanic	16 580 (74)	19 966 (80)	−0.13
Unknown	1859 (8)	2564 (10)	−0.07
Race^e^			
Black	3438 (15)	7013 (28)	−0.31
White	11 181 (50)	10 741 (43)	0.14
Other^f^	3623 (16)	3533 (14)	0.06
Unknown	4076 (18)	3702 (15)	0.09
Language			
English	20 295 (91)	22 283 (89)	0.06
Other^g^	570 (3)	574 (2)	0.02
Missing or declined	1453 (7)	2132 (9)	−0.08

Abbreviations: NA, not applicable; SMD, standardized mean difference.

^a^ SMD was calculated as (x̅_N+_ − x̅_N_) / [(σ_N+_ + σ_N_)/2], where x̅_N_, σ_N_ and x̅_N+_, σ_N+_ indicate mean (SD) for nudge and nudge+, respectively, calculated across visit-level observations (eg, x̅_N_ is the mean of all visit-level observations within nudge; for clinic-level variables all visits within a clinic have the same value). Variables with absolute value SMD ≥0.2 were included in the adjusted model(s).

^b^ Defined as the number of well-child visits at the clinic in the calendar year prior to the trial (2021).

^c^ Clinician partners were clinicians or administrative leaders who advised on study design and delivery of the program across clinics.

^d^ For each clinic, we calculated the percentage of well-child visits in the calendar year preceding the trial (2021) with patients with addresses in rural areas—ie, a micropolitan or noncore area according to the National Center for Health Statistics Urban-Rural Classification Scheme for Counties 2013.^43^

^e^ Reporting race and ethnicity in this study was mandated by the US National Institutes of Health. Additionally, we used race and ethnicity to characterize the representativeness of the study population, generalizability of the results, and provide information about potential disparities and inequities.

^f^ Including Asian, American Indian or Alaska Native, Hawaiian/Other Pacific Islander, other, and more than one race. These groups were consolidated owing to small numbers.

^g^ Spanish was endorsed as the primary language by 1.2% of the sample (n = 554). All other primary languages (n = 53) were endorsed by less than 1% of the sample.

### Effectiveness of Strategies: Reach

**Figure 2.**
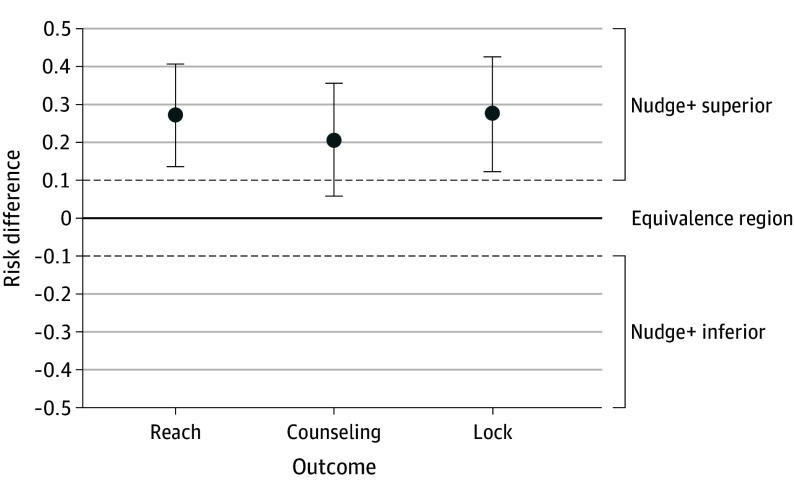
Adjusted Model Results for the Risk Difference in Reach and Its 2 Components The 95% CI generated by the generalized estimating equation model for the risk difference of the primary outcome (reach) between the nudge condition and the nudge+ condition was (0.14-0.41). The 97.5% CIs for the risk difference between conditions for our secondary outcomes were as follows: counseling, 0.06-0.36 and lock, 0.12-0.43. Risk differences were computed using a generalized estimating equation model that adjusted for health care system, clinic size, clinician partner at clinic, patient sex, and an indicator of Hispanic ethnicity. Because the CIs for reach and lock are both contained within the nudge+ superior region alone, we can conclude that nudge+ was significantly better than nudge at a 10% margin for both reach and lock. Because the CI for counseling partially overlaps with the equivalence region, nudge+ cannot be considered superior for counseling.

Based on our adjusted model (Table 3), a higher percentage of well-child visits received both program components of the firearm storage program (ie, reach) as documented by the clinician in the EHR in the nudge+ condition (49%; 95% CI, 37-61) compared to nudge (22%; 95% CI, 13-31). Figure 2 presents 2-sided 95% CIs for reach and 97.5% CIs for each of its 2 components (counseling and lock) by condition. The lower limit of the confidence interval for the risk difference in reach between the groups exceeded the equivalence margin of 0.1. Based on this result, we can conclude that nudge+ had superior reach compared to nudge with statistical significance (*P* < .05) at the prespecified margin of 0.1. Results based on unadjusted models and adjusted models with alternative specifications were similar to those based on the adjusted models (eResults in Supplement 2).

**Table 3.** Primary Outcomes From Adjusted Model^a^

Program component	Marginal probability (95% CI for reach, 97.5% CI for counseling and lock)^b^		
	Nudge+	Nudge	Nudge+ − nudge
Reach^c^	0.49 (0.37-0.61)	0.22 (0.13-0.31)	0.27 (0.14-0.41)
Counseling	0.61 (0.49-0.73)	0.41 (0.28-0.53)	0.21 (0.06-0.36)
Lock	0.49 (0.36-0.63)	0.22 (0.12-0.32)	0.27 (0.12-0.43)

^a^ Generalized estimating equation with exchangeable working correlation, logit link, and Kauermann-Carroll (small cluster) correction. A logit link was used to obtain the marginal probabilities. Covariates in adjusted model: variables used to constrain the clinic-level randomization (health system, clinic size, and clinician partner at clinic), variables prespecified based on results from our team’s preimplementation pilot (patient sex), and variables with an absolute standardized mean difference value that exceeded 0.2 (Hispanic ethnicity). Black race exceeded an absolute standardized mean difference value of 0.2, but models with both health care system and race failed to converge, and thus race was removed as a covariate from all adjusted models.

^b^ Marginalized over the empirical distribution of model covariates. The marginal probability of each outcome can take a value between 0 and 1, with values close to 0 indicating low delivery and values close to 1 indicating high delivery. The risk difference in the rightmost column can take values −1 to 1 with positive values indicating higher delivery under nudge+ than nudge.

^c^ Reach is a composite outcome consisting of counseling and lock and thus is driven by the component with lower delivery rate.

### Secondary Outcomes: Reach by Program Component

Nudge+ demonstrated superiority to nudge on clinician-reported offer of a cable lock alone, but not on clinician-reported counseling alone, at a margin of 0.1. The estimate of the risk difference for the lock component was 0.27 (97.5% CI, 0.12-0.43), and for counseling it was 0.21 (97.5% CI, 0.06-0.36). The lower limit of the 97.5% CI for lock exceeded the 0.1 equivalence margin, indicating superiority of nudge+. The lower limit of the 97.5% CI for counseling did not exceed the 0.1 equivalence margin; however, it exceeded zero, suggesting that nudge+ resulted in higher delivery of counseling. Results based on the unadjusted models were similar to those based on the adjusted models (eResults in Supplement 2).

## Discussion

In this cluster randomized trial, we implemented an evidence-based program for secure firearm storage in 30 pediatric primary care clinics across 2 geographically, demographically, and culturally diverse health systems, and found that an EHR strategy with facilitation was more effective at increasing the firearm storage program reach (49%) compared to the EHR strategy alone (22%). Based on a clinically significant margin of 10 percentage points, this finding provides support for both clinical and statistical significance. Secondary analyses indicated that facilitation significantly increased the likelihood that a clinician would offer a lock, but not counseling. This work represents one of the first studies conducted across multiple health systems and leveraging rigorous methods from implementation science to investigate how to routinize secure firearm storage programs within pediatric primary care well visits as a universal population health approach. Prior work has primarily used quality improvement methods to increase documentation of firearm screening and/or access in the EHR and has demonstrated heterogeneity in effectiveness.^44,45,46,47,48^ Previous studies that used a combination of educational and EHR-based strategies corroborate our findings that multiple discrete strategies, rather than education or EHR-based strategies alone, may more fully support clinician behavior change to close research-to-practice gaps.^46,49^

We found that when implemented alone, the EHR strategy, which was designed with insights from behavioral economics,^25,26,27^ resulted in modest but meaningful change in clinician behavior vs rates of baseline delivery of counseling and lock offers observed in previous work. For example, previous work found that only 2% of clinicians report both delivering counseling about secure storage and offering a cable lock most or all the time.^11^ Facilitation,^50^ which consisted of internal health system personnel providing support to clinicians and clinics around deployment of the new program, emerged as an important approach for boosting behavior change, consistent with prior literature.^51^ While facilitation included a number of discrete activities, the most commonly delivered activities included providing feedback, providing updates, and ongoing action and implementation planning. Providing feedback includes sharing clinical performance data related to reach with clinicians and administrators to support implementation, while providing updates includes sharing general information about the project or health system that is relevant to implementation. Ongoing action and implementation planning refers to using and updating the implementation plan, developed at the beginning of the trial, to guide the implementation effort over the year. Our findings support the hypothesis that in the case of this secure firearm storage program, facilitation, which provides targeted support to implementers, is an effective strategy. Notably, facilitation offered within this trial was not resource or time intensive, although it required staff who were trained in deploying this strategy, but nonetheless resulted in a robust effect. Health systems considering how best to implement the firearm storage program in their contexts should consider which strategies are most feasible for them to deploy this evidence-based program while balancing resources needed, available staffing, and scalability.

This trial built on preimplementation work that likely contributed to the success of this effort. This formative work included interviews with all affected constituent groups,^16,20^ systematic adaptation^13^ of the program centering voices of end users,^14^ development of a blueprint for implementation via implementation mapping,^52,53^ and a pilot trial^35^ to problem-solve barriers prior to fully powered trial execution. Further, this work was done within the context of a broader sociopolitical context that was affected by increasing rates of firearm injury and death in young people. However, even after observing robust practice change with respect to the firearm storage program delivery in this trial, there is still room for improvement. Greater implementation across well-child visits is needed for this approach to fully realize its potential. Our qualitative work^54^ with clinicians and leaders in these clinics, which is ongoing, will allow us to better understand barriers to implementation including patient-, clinician-, clinic-, system-, and societal-level barriers to further improve our approach. Generally, more work is needed to ensure that secure firearm storage information reaches all families, even if it cannot always occur within the context of well-child visits due to competing priorities and time constraints or in large health care systems, as in this trial. Potential complementary approaches could include messages through patient portals following visits and implementation of the firearm storage program in other contexts where children and parents frequent (eg, community health centers or federally qualified health centers, adult primary care, afterschool programs, and faith-based organizations).

### Limitations

Although this study has methodological strengths, including a demographically diverse participant sample, rigorous randomized design, and constituent-partnered approach, it has limitations. These include: (1) no control condition (as required by the health care systems we were working with); (2) the limitations of EHR data and strategies (ie, our primary outcome extracted from the EHR does not allow us to understand granular delivery of the program [ie, depth, quality, and dosage]; we were not able to extract parent-level characteristics; and not all clinicians actively document in the EHR during visits, thus potentially reducing the effectiveness of the EHR strategy); (3) patients, clinicians, and clinics could have differed at baseline in important but unmeasured ways; (4) heterogeneity in health care systems regarding involvement of trainee clinicians which we were unable to measure^49^; and (5) only providing cable locks, given recent work suggesting that they may not be the preferred locking mechanism for firearms that are kept for protection purposes.^55,56^ We provided cable locks given they are the most scalable approach for distribution in health care systems. After-visit summaries and parent handouts at clinics linked to the study website, which described alternative locking mechanisms and resources for those who did not prefer cable locks; we view the locks as an entry point for the discussion between the clinician and parent.

## Conclusions

Our EHR and facilitation strategies led to robust practice change related to the firearm storage program delivery during pediatric primary care visits across 2 health care systems, with the EHR strategy plus facilitation being more effective at increasing program reach compared to the EHR strategy alone. Future work should explore the impact of program delivery on parental secure firearm storage, which is the primary mechanism through which injury and mortality risk is reduced for young people.

## Notes

Supplement 1. Trial protocol and statistical analysis plan

Supplement 2. eResults eFigure. Approximation of EHR nudges at the two health systems (i.e., not exact replicas or copies)

Supplement 3. Data sharing statement

## References

[1] Goldstick JE, Cunningham RM, Carter PM. Current causes of death in children and adolescents in the United States. N Engl J Med. 2022;386(20):1955-1956. doi:10.1056/NEJMc2201761 35443104 PMC10042524

[2] Garcia S, Entrup P, Hall OT, Deaner M, Thomas A, Lim R. Years of life lost to firearm suicide among young people in the US. JAMA Pediatr. 2023;177(11):1230-1232. doi:10.1001/jamapediatrics.2023.3366 37669072 PMC10481317

[3] United States Department of Health and Human Services, Centers for Disease Control and Prevention, National Center for Health Statistics. Underlying cause of death 1999-2020 on CDC WONDER online database. Accessed October 10, 2023. https://wonder.cdc.gov/

[4] US Centers for Disease Control and Prevention. Web-based injury statistics query and reporting system (WISQARS). Accessed February 6, 2023. https://www.cdc.gov/injury/wisqars/index.html

[5] Miller M, Azrael D. Firearm storage in US households with children: findings from the 2021 National Firearm Survey. JAMA Netw Open. 2022;5(2):e2148823. doi:10.1001/jamanetworkopen.2021.48823 35191973 PMC8864510

[6] Barber C, Azrael D, Miller M, Hemenway D. Who owned the gun in firearm suicides of men, women, and youth in five US states? Prev Med. 2022;164:107066. doi:10.1016/j.ypmed.2022.107066 35461957

[7] Monuteaux MC, Azrael D, Miller M. Association of increased safe household firearm storage with firearm suicide and unintentional death among US youths. JAMA Pediatr. 2019;173(7):657-662. doi:10.1001/jamapediatrics.2019.1078 31081861 PMC6515586

[8] Rowhani-Rahbar A, Simonetti JA, Rivara FP. Effectiveness of interventions to promote safe firearm storage. Epidemiol Rev. 2016;38(1):111-124. doi:10.1093/epirev/mxv006 26769724

[9] Lee LK, Fleegler EW, Goyal MK, . Firearm-related injuries and deaths in children and youth. Pediatrics. 2022;150(6):e2022060071. doi:10.1542/peds.2022-060071 36207778

[10] Hagan JF, Shaw JS, Duncan PM, eds. Bright Futures: Guidelines for Health Supervision of Infants, Children, and Adolescents. 4th ed. American Academy of Pediatrics; 2017. doi:10.1542/9781610020237

[11] Beidas RS, Jager-Hyman S, Becker-Haimes EM, . Acceptability and use of evidence-based practices for firearm storage in pediatric primary care. Acad Pediatr. 2019;19(6):670-676. doi:10.1016/j.acap.2018.11.007 30508600 PMC6542719

[12] US Department of Health and Human Services: Office of the Surgeon General. Firearm violence: a public health crisis in America: the U.S. Surgeon General’s advisory 2024. Accessed June 25, 2024. https://www.hhs.gov/sites/default/files/firearm-violence-advisory.pdf 39042747

[13] Davis M, Johnson C, Pettit AR, . Adapting safety check as a universal suicide prevention strategy in pediatric primary care. Acad Pediatr. 2021;21(7):1161-1170. doi:10.1016/j.acap.2021.04.012 33901726 PMC8429196

[14] Hoskins K, Johnson C, Davis M, . A mixed methods evaluation of parents’ perspectives on the acceptability of the S.A.F.E. Firearm program. J Appl Res Child. 2021;12(2):2. doi:10.58464/2155-5834.1473 36883133 PMC9987154

[15] Barkin SL, Finch SA, Ip EH, . Is office-based counseling about media use, timeouts, and firearm storage effective? results from a cluster-randomized, controlled trial. Pediatrics. 2008;122(1):e15-e25. doi:10.1542/peds.2007-2611 18595960 PMC4486046

[16] Benjamin Wolk C, Van Pelt AE, Jager-Hyman S, . Stakeholder perspectives on implementing a firearm safety intervention in pediatric primary care as a universal suicide prevention strategy: a qualitative study. JAMA Netw Open. 2018;1(7):e185309. doi:10.1001/jamanetworkopen.2018.5309 30646398 PMC6324366

[17] Beidas RS, Ahmedani BK, Linn KA, . Study protocol for a type III hybrid effectiveness-implementation trial of strategies to implement firearm safety promotion as a universal suicide prevention strategy in pediatric primary care. Implement Sci. 2021;16(1):89. doi:10.1186/s13012-021-01154-8 34551811 PMC8456701

[18] Moulton LH. Covariate-based constrained randomization of group-randomized trials. Clin Trials. 2004;1(3):297-305. doi:10.1191/1740774504cn024oa 16279255

[19] American Academy of Pediatrics. Safe storage of firearms. Accessed August 5, 2024. https://www.aap.org/en/patient-care/gun-safety-and-injury-prevention/safe-storage-of-firearms/

[20] Jager-Hyman S, Benjamin Wolk C, Ahmedani BK, . Perspectives from firearm stakeholders on firearm safety promotion in pediatric primary care as a suicide prevention strategy: a qualitative study. J Behav Med. 2019;42(4):691-701. doi:10.1007/s10865-019-00074-9 31367934 PMC7603788

[21] Beidas RS, Rivara F, Rowhani-Rahbar A. Safe firearm storage: a call for research informed by firearm stakeholders. Pediatrics. 2020;146(5):e20200716. doi:10.1542/peds.2020-0716 33037120 PMC7605082

[22] Lee LK, Fleegler EW, Goyal MK, . Firearm-related injuries and deaths in children and youth: injury prevention and harm reduction. Pediatrics. 2022;150(6):e2022060070. doi:10.1542/peds.2022-060070 36207776

[23] Hawk M, Coulter RWS, Egan JE, . Harm reduction principles for healthcare settings. Harm Reduct J. 2017;14(1):70. doi:10.1186/s12954-017-0196-4 29065896 PMC5655864

[24] SAFE Firearm materials. https://redcap.link/SAFEFirearm

[25] Harrison JD, Patel MS. Designing nudges for success in health care. AMA J Ethics. 2020;22(9):E796-E801. doi:10.1001/amajethics.2020.796 33009777

[26] Last BS, Buttenheim AM, Timon CE, Mitra N, Beidas RS. Systematic review of clinician-directed nudges in healthcare contexts. BMJ Open. 2021;11(7):e048801. doi:10.1136/bmjopen-2021-048801 34253672 PMC8276299

[27] Volpp K, Delgado MK. Behavioral nudges are used widely to steer clinicians and patients alike. NEJM Catal. 2023;4(6). doi:10.1056/CAT.23.0125

[28] Shelley DR, Ogedegbe G, Anane S, . Testing the use of practice facilitation in a cluster randomized stepped-wedge design trial to improve adherence to cardiovascular disease prevention guidelines: HealthyHearts NYC. Implement Sci. 2016;11(1):88. doi:10.1186/s13012-016-0450-2 27377404 PMC4932668

[29] Michaels L, Anastas T, Waddell EN, Fagnan L, Dorr DA. A randomized trial of high-value change using practice facilitation. J Am Board Fam Med. 2017;30(5):572-582. doi:10.3122/jabfm.2017.05.170013 28923809 PMC6599689

[30] Parchman ML, Noel PH, Culler SD, . A randomized trial of practice facilitation to improve the delivery of chronic illness care in primary care: initial and sustained effects. Implement Sci. 2013;8:93. doi:10.1186/1748-5908-8-93 23965255 PMC3765887

[31] Weiner BJ, Rohweder CL, Scott JE, . Using practice facilitation to increase rates of colorectal cancer screening in community health centers, North Carolina, 2012-2013: feasibility, facilitators, and barriers. Prev Chronic Dis. 2017;14:E66. doi:10.5888/pcd14.160454 28817791 PMC5566800

[32] Persell SD, Liss DT, Walunas TL, . Effects of 2 forms of practice facilitation on cardiovascular prevention in primary care: a practice-randomized, comparative effectiveness trial. Med Care. 2020;58(4):344-351. doi:10.1097/MLR.0000000000001260 31876643

[33] Liang KY, Zeger SL. Longitudinal data analysis of continuous and discrete responses for pre-post designs. Sankhya. 2000;62(1):134-148. doi:10.2307/25053123

[34] Hubbard AE, Ahern J, Fleischer NL, . To GEE or not to GEE: comparing population average and mixed models for estimating the associations between neighborhood risk factors and health. Epidemiology. 2010;21(4):467-474. doi:10.1097/EDE.0b013e3181caeb90 20220526

[35] Hoskins K, Linn KA, Ahmedani BK, . Equitable implementation of S.A.F.E. Firearm: a multi-method pilot study. Prev Med. 2022;165(Pt A):107281. doi:10.1016/j.ypmed.2022.107281 36191653 PMC10013361

[36] Kauermann G, Carroll RJ. A note on the efficiency of sandwich covariance matrix estimation. J Am Stat Assoc. 2001;96(456):1387-1396. doi:10.1198/016214501753382309

[37] Gallis JA, Li F, Turner EL. xtgeebcv: A command for bias-corrected sandwich variance estimation for GEE analyses of cluster randomized trials. Stata J. 2020;20(2):363-381. doi:10.1177/1536867X20931001 35330784 PMC8942127

[38] Goeman JJ, Solari A, Stijnen T. Three-sided hypothesis testing: simultaneous testing of superiority, equivalence and inferiority. Stat Med. 2010;29(20):2117-2125. doi:10.1002/sim.4002 20658478

[39] Thiele H, Kurz T, Feistritzer HJ, ; SOLVE-TAVI Investigators. General versus local anesthesia with conscious sedation in transcatheter aortic valve implantation: the randomized SOLVE-TAVI trial. Circulation. 2020;142(15):1437-1447. doi:10.1161/CIRCULATIONAHA.120.046451 32819145

[40] Sirochman AL, Milovancev M, Townsend K, Grimes JA. Influence of use of a bipolar vessel sealing device on short-term postoperative mortality after splenectomy: 203 dogs (2005-2018). Vet Surg. 2020;49(2):291-303. doi:10.1111/vsu.13367 31837169

[41] Meyer A, Rudant J, Drouin J, Weill A, Carbonnel F, Coste J. Effectiveness and safety of reference infliximab and biosimilar in Crohn disease: a French equivalence study. Ann Intern Med. 2019;170(2):99-107. doi:10.7326/M18-1512 30534946

[42] Penfold RB, Thompson EE, Hilt RJ, . Safer use of antipsychotics in youth (SUAY) pragmatic trial protocol. Contemp Clin Trials. 2020;99:106184. doi:10.1016/j.cct.2020.106184 33091587 PMC7726008

[43] National Center for Health Statistics. NCHS urban-rural classification scheme for counties. Accessed June 1, 2021. https://www.cdc.gov/nchs/data_access/urban_rural.htm

[44] Stipelman CH, Stoddard G, Bata K, Muniyappa B, Trepman E, Smith E. Home gun safety queries in well-child visits. JAMA Pediatr. 2019;173(12):1205-1208. doi:10.1001/jamapediatrics.2019.3845 31657850 PMC6820029

[45] Oddo ER, Kumar N, Andrews AL, Kwon S. Firearm safety screening in the pediatric hospital setting: a quality improvement initiative. Pediatr Qual Saf. 2023;8(5):e689. doi:10.1097/pq9.0000000000000689 37780602 PMC10538933

[46] Kemal S, Lennon T, Simon NJ, . Improving documentation of firearm access during pediatric emergency visits for suicidal ideation. Pediatrics. 2024;153(4):e2023063447. doi:10.1542/peds.2023-063447 38426287

[47] Hogan AH, Gadun A, Borrup K, . Assessing the effect of electronic medical record note template on firearm access screening in high-risk children. Hosp Pediatr. 2022;12(8):e278-e282. doi:10.1542/hpeds.2022-006515 35794213

[48] Naureckas Li C, Sacks CA, Cummings BM, Samuels-Kalow M, Masiakos PT, Flaherty MR. Improving pediatric residents’ screening for access to firearms in high-risk patients presenting to the emergency department. Acad Pediatr. 2021;21(4):710-715. doi:10.1016/j.acap.2021.01.002 33429102

[49] Gastineau KAB, Stegall CL, Lowrey LK, Giourgas BK, Andrews AL. Improving the frequency and documentation of gun safety counseling in a resident primary care clinic. Acad Pediatr. 2021;21(1):117-123. doi:10.1016/j.acap.2020.07.013 32673765

[50] Ritchie M, Dollar K, Miller C, . Using implementation facilitation to improve healthcare (version 3). Veterans Health Administration Behavioral Health Quality Enhancement Research Initiative (QUERI). Accessed October 10, 2023. https://www.queri.research.va.gov/tools/Facilitation-Manual.pdf

[51] Baskerville NB, Liddy C, Hogg W. Systematic review and meta-analysis of practice facilitation within primary care settings. Ann Fam Med. 2012;10(1):63-74. doi:10.1370/afm.1312 22230833 PMC3262473

[52] Fernandez ME, Ten Hoor GA, van Lieshout S, . Implementation mapping: using intervention mapping to develop implementation strategies. Front Public Health. 2019;7:158. doi:10.3389/fpubh.2019.00158 31275915 PMC6592155

[53] Wolk CB, Jager-Hyman S, Marcus SC, . Developing implementation strategies for firearm safety promotion in paediatric primary care for suicide prevention in two large US health systems: a study protocol for a mixed-methods implementation study. BMJ Open. 2017;7(6):e014407. doi:10.1136/bmjopen-2016-014407 28647722 PMC5541509

[54] Waller C, Pandey M, Boggs J, . A qualitative analysis of how two implementation strategies in a hybrid effectiveness-implementation trial supported implementation of a secure firearm storage program. Oral presentation at the 7th Biennial Meeting of the Society for Implementation Research Collaboration; September 27-28, 2024; Denver, CO.

[55] Betz ME, Stanley IH, Buck-Atkinson J, . Firearm owners’ preferences for locking devices: results of a national survey. Ann Intern Med. 2023;176(3):424-427. doi:10.7326/M22-3113 36745884

[56] Buck-Atkinson J, McCarthy M, Stanley IH, . Firearm locking device preferences among firearm owners in the USA: a systematic review. Inj Epidemiol. 2023;10(1):33. doi:10.1186/s40621-023-00436-7 37415242 PMC10326943

