# Supplementary material for: Implementation of a Secure Firearm Storage Program in Pediatric Primary Care: A Cluster Randomized Trial
Source: JAMA Pediatr. 2024 Sep 3;178(11):1104–13. doi: 10.1001/jamapediatrics.2024.3274 (PMC11372656; doi:10.1001/jamapediatrics.2024.3274)
Supplement: Supplement 1. — Trial protocol and statistical analysis plan [file jamapediatr-e243274-s001.pdf]

Study Protocol & Statistical Analysis Plan (SAP)
Adolescent and child Suicide Prevention In Routine clinical Encounters (ASPIRE) study

Official Title: A comparative effectiveness trial of strategies to implement firearm safety promotion as a universal suicide prevention strategy in pediatric primary care

Funding Agency: National Institute of Mental Health (NIMH)

NIH Grant Number: R01 MH123491

University of Pennsylvania Protocol Number: 844327

Principal Investigator: Rinad S. Beidas, PhD

ClinicalTrials.gov Number: NCT04844021

Original Study Protocol: 08 February 2022

Final Study Protocol: 03 January 2023

Original SAP: 09 August 2021

Final SAP: 03 July 2023

Table of Contents

- **IRB protocol** 2
  - **Final approved protocol** 2
  - **Protocol amendments** 23
  - **Initial approved protocol** 25
- **Statistical Analysis Plan** 45
  - **Final Statistical Analysis Plan** 45
  - **SAP amendments** 47
  - **Original Statistical Analysis Plan** 49
  - **Study blinding details** 50
- **References** 51

IRB protocol

Final approved protocol

Approved by the University of Pennsylvania Single IRB: 03 January 2023

Social and Behavioral Sciences Human Research Protocol

PRINCIPAL INVESTIGATOR (Northwestern University):
Rinad Beidas, PhD
PHONE; EMAIL

PRINCIPAL INVESTIGATOR (University of Pennsylvania):
Kristin Linn, PhD
PHONE; EMAIL

PROTOCOL TITLE: A comparative effectiveness trial of strategies to implement firearm safety promotion as a universal suicide prevention strategy in pediatric primary care

INTRODUCTION AND PURPOSE:
The number of young people dying by suicide continues to rise in the U.S.[1,2] The risk for suicide death is much greater when there is an unlocked and loaded firearm in the home.[3] Two lines of evidence indicate that pediatric primary care is an ideal setting for universal prevention to reduce firearm access and related suicide risk. First, most youth visit primary care each year.[4] One study found that 77% of youth who died by suicide had accessed primary care in the prior year, with nearly 40% attending a visit within 4 weeks of death.[5] Second, studies have found that programs deployed in primary care can increase safe firearm storage among parents.[6-9] The most rigorous study tested *Safety Check*, found that parents in the intervention group increased safe firearm storage compared to the control group.[6] These results led the American Academy of Pediatrics[10] and National Academy of Medicine[11] to recommend implementation of evidence-based safe firearm storage programs like *Safety Check,* but the core components of the program have yet to become routine clinical practice. Implementation research is urgently needed to realize the potential of this program,[12-15] especially given that a simulation study found that even a modest increase in safe firearm storage could prevent up to 32% of firearm-related deaths in youth due to both suicide and accidents.[16]

The current proposal builds on a previous NIMH-funded project (IRB number 824449) conducted in the Mental Health Research Network (MHRN), in which we interviewed 70 stakeholders, including pediatric clinicians, health system leaders, firearm safety experts, and parents who own firearms. We queried about needed adaptations to *Safety Check*, implementation barriers, and preferred implementation strategies guided by the Consolidated Framework for Implementation Research (CFIR), a leading implementation science framework.[17-19] Adaptation recommendations included renaming the program (now called *SAFE Firearm*), removing the formal screening step and any documentation of firearm ownership in the electronic health record (EHR), and offering brief counseling on safe storage and cable locks to all families.[17,18] The most frequent implementation strategy suggestion was to integrate the program into the EHR. Studies support the effectiveness and scalability of EHR approaches to make implementation of new practices more salient and part of workflow.[20-22] Specifically, based on principles of behavioral economics, a well-designed EHR order set can 'nudge' clinician behavior.[23] However, our interviews identified implementation barriers that may warrant additional strategies, including clinician comfort with the program and clinic workflow.[19] Facilitation is an evidence-based implementation approach that focuses on building organizational capacity for improvement and directly targets the unique implementation barriers experienced by each clinic.[24-26] We will answer: Is the less costly and scalable EHR-based 'nudge' powerful enough to increase implementation of SAFE Firearm or is more intensive and expensive facilitation needed to overcome implementation barriers?

We will conduct a hybrid type III effectiveness-implementation trial with a longitudinal cluster randomized design to answer questions related to implementation strategy and program effectiveness. We will also apply mixed methods to investigate implementation strategy mechanisms. During the active implementation period, all 30 clinics in the two participating health systems will receive *SAFE Firearm* materials, including brief training in program

delivery, cable locks, and the deployment of an EHR implementation strategy. Half of the clinics will be randomized to only receive the EHR implementation strategy (*Nudge*); the other half will be randomized to receive Nudge plus 1 year of facilitation to target additional clinician and organizational implementation barriers (*Nudge+*). We will collect the primary implementation outcome for 1 year following the active trial year to allow for comparisons between the active and sustainment periods. Results will guide future efforts to promote firearm safety as a universal suicide prevention strategy.

EQUITY SUPPLEMENT PURPOSE:

Prior to launching the full trial, we are piloting the delivery of the *SAFE Firearm* program in 2 clinics in each health system during an approximately 4-5 month period. Pilot procedures will mirror the procedures described in the protocol and use the same parent survey instrument (more details are below). After the pilot, we will conduct this sub-study. Specifically, we will explore any signals of disparities and examine *SAFE Firearm* delivery by children's medical complexity (per ICD-9 and -10 codes) and race/ethnicity. Because we anticipate that clinicians will provide helpful contextualization for the program that will be informative for facilitation for the program and for the broader literature on firearm safety counseling, regardless of whether we detect differences in program delivery, we will interview KPCO and HFH clinicians who participated in the pilot to identify specific delivery barriers, and convene key stakeholders (e.g., facilitation leaders, pediatric leadership) for virtual meetings. We expect that this step will prime facilitation for trial launch. The goal of this sub-study is to learn more about barriers to delivering the *SAFE Firearm* program equitably to all patients, regardless of medical complexity and race/ethnicity, and to optimize facilitation to address disparities.

An important note about what we are requesting the IRB to review as part of this protocol: Leadership within both health systems has agreed to deploy the Evidence-Based Program (i.e. SAFE Firearm) and the Implementation Strategies (i.e. EHR Nudges, Facilitation) within their respective systems. They also are in support of conducting the Research Activities (i.e. quantitative surveys, qualitative interviews, and EHR/Administrative data extraction) to learn more about the implementation of the SAFE Firearm program as well as about EHR Nudge and Facilitation strategies. We have described the SAFE Firearm program as well as the EHR Nudge and Facilitation strategies in this protocol for reference, but we are requesting IRB review and oversight of only the Research Activities. The SAFE Firearm program is evidence-based and the primary research question isn't about effectiveness of this program but rather about implementation. Parents may receive SAFE Firearm during a pediatric primary care appointment and not participate in the research survey and vice versa. Requiring consent from participants to receive the SAFE Firearm program, EHR Nudge, and/or facilitation would make this implementation research study impossible to conduct because it would create undue bias and be overly burdensome on health systems. The program and strategies are low-risk, evidence-based, and prevention oriented, and ultimately the decision to deliver the SAFE Firearm program is left to the individual clinicians for each family. We are looking to answer pragmatic questions about the implementation strategies in a healthcare setting, i.e. is the less costly and scalable EHR-based 'nudge' powerful enough to increase implementation of SAFE Firearm or is more intensive and expensive facilitation needed to overcome implementation barriers? The purpose of this study is not to establish SAFE Firearm as an effective program, because that has already been done, but rather to study which strategy improves clinician adherence to the program in a healthcare setting (as reported by clinicians).

OBJECTIVES:

- <u>Aim 1</u>. Conduct a longitudinal cluster randomized trial to test the comparative effectiveness of two active implementation strategies, *Nudge* (EHR) vs. *Nudge+* (EHR + facilitation).
- <u>Aim 2</u>. To use mixed methods to identify implementation strategy moderators and mediators.
- <u>Aim 3</u>. To examine the effects of SAFE Firearm on patient clinical outcomes.

Primary outcome variables:

- <u>Aim 1</u>. Clinician-reported reach (program delivery as documented in the EHR)
- <u>Aim 2</u>. Staff-reported clinic adaptive reserve
- <u>Aim 3</u>. Parent-reported firearm storage behavior

Secondary/Other outcome variables (Moderators and mediators to be measured):

- <u>Aim 1</u>. Parent-reported clinician fidelity, cable lock distribution, acceptability, cost
- <u>Aim 2</u>.

    - *Moderators*: Clinician burnout, clinician attitudes towards firearms counseling
    - *Mediators:* Clinician motivation
- Aim 3. Youth suicide attempts, deaths, and unintentional firearm injury and mortality

BACKGROUND:

Youth suicide deaths are on the rise in the United States (U.S.). Firearms are a common and lethal method of suicide attempt. Reducing access to lethal means is a promising yet underused suicide prevention strategy.[27] Firearms are a critical target of such efforts [28,29] given that they are present in one in three U.S. homes and these numbers are increasing in the wake of Covid-19.[30] Recent research has found that only 3 in 10 firearm-owning families with children store all guns in their home in the safest manner: locked and unloaded. Approximately 4.6 million U.S. children (7%) live in homes in which at least one firearm is stored unlocked and loaded.[31] Given that the presence of firearms in the home is a robust risk factor for suicide,[32] safe storage of firearms in the home is imperative for reducing youth suicide attempts and death. Simulation research has found that even a modest increase in safe firearm storage could prevent as many as 32% of youth firearm deaths due to suicide and accidents.[16] Thus, efforts to increase implementation of interventions to improve safe storage could save young lives nationally.

The success of lethal means safety interventions in other settings highlights their potential value in primary care as a strategy to save young lives. Means safety interventions have been shown to be effective in specialty behavioral health,[33] emergency departments,[34] and in the community.[35] While these efforts are important, primary care represents a critical opportunity for universal suicide prevention, as it has long served as the de facto mental health services system.[36] For example, whereas only one third of youth receive mental health treatment in the year prior to a suicide attempt,[37,38] four out of five youth who die by suicide visit primary care during that time period.[37] Further, at least half of individuals who die by suicide with a firearm have no known psychiatric history,[39,40] underscoring the importance of universal prevention. With 90% of youth visiting primary care annually[41] and a high rate of primary care visits among youth who die by suicide in the year prior,[42] primary care provides an excellent opportunity for universal firearm safety intervention.

Safety Check is an evidence-based, pediatric primary care program targeting parental safe firearm storage as part of a bundle of violence prevention strategies.[6] The program includes screening for firearms in the home, brief counseling around safe storage, and providing cable locks. A large clinical trial in 137 pediatric practices found that parents receiving *Safety Check* reported double the odds of safe storage (OR = 2.0, $p < .011$) compared to the control group. The intervention group showed a 10% increase in use of cable locks, while there was a 12% decrease in safe storage in the control group ($p < .001$). While these results led major professional organizations to recommend implementation of safe firearm storage programs like *Safety Check*, they have not been routinely implemented.[14] There are a number of potential explanations for slow uptake. One potential reason may be the fit of the program for primary care (i.e., program-setting fit).[17] To investigate whether this might be the case and to increase our understanding of how best to implement *Safety Check* as a universal suicide prevention strategy, we conducted pre-implementation work in two health systems, guided by CFIR.[17] This work was particularly salient given that in the 15 years since the *Safety Check* program was designed, there have been advances in implementation science and an increasingly sensitive national debate about firearms, including an evolving role of professional medical associations in advocating for firearm safety as a public health approach. Our preliminary work allowed us to gather key information about barriers and facilitators to the implementation of *Safety Check* in primary care as a universal suicide prevention strategy within the current national context, including primary care provider (PCP) attitudes about discussing firearm safety with parents and firearm stakeholders' attitudes about PCPs raising this topic.[14,18] Recent work funded through a pilot grant from the FACTS Consortium, an NICHD-funded R24, has allowed us to incorporate stakeholder feedback from this previous work and additional pilot work to adapt *Safety Check* to increase its feasibility and acceptability. This has paved the way for adaptations to the program, now called *SAFE Firearm*, and the development of implementation strategies to be tested in the proposed trial.

Implementation strategies informed by stakeholder input and behavioral economics have the potential to increase use of *SAFE Firearm* in routine pediatric practice. Behavioral economics focuses on the power of context and an individual's limited resources (e.g., time, attention) in shaping decisions and behavior,[43] thus allowing for the identification of common, predictable cognitive heuristics or shortcuts that people use in making decisions.[44,45] These heuristics can be accommodated and harnessed through choice architecture, which involves changing the environment to facilitate the desired evidence-based choice.[46] One of the most powerful ways to use choice architecture to affect behavior is through nudges, which involve making subtle changes to the way that choices are presented.[47] These approaches have been leveraged to change clinician practice in multiple areas of medicine[20,48-50]

and have yielded promising effects when applied to mental health in our NIMH-funded P50 Penn ALACRITY Center (MPIs: Beidas, Mandell, Buttenheim).[46] Nudges deployed through the electronic health record (EHR) have the capacity to prompt and shape medical decision-making in unobtrusive ways that do not disrupt workflow or add more tasks and have the potential to be effective and low-cost.[20,22,51,52] Given that more than 90% of U.S. hospitals use an EHR[53] and that the highest rates of EHR adoption are in primary care,[54] EHR implementation strategies are also scalable. At the same time, our stakeholder research revealed that additional, more intensive implementation strategies may be needed to maximize implementation success, particularly within the context of a sensitive topic such as firearm safety.

The proposed research draws on multiple streams of evidence to maximize impact in the context of an urgent and sensitive topic and incorporates the latest advances in implementation science. Despite the promise, behavioral economics and implementation science approaches have not been systematically integrated and applied to suicide prevention. Merging these fields of study offers an opportunity to test the support needed for implementation of *SAFE Firearm* and will also provide unique insights into implementation of evidence-based practices in primary care more broadly. Testing these strategies in the context of a hybrid effectiveness-implementation trial will also aid in reducing youth suicide deaths. Additionally, despite the proliferation of conceptual frameworks[55,56] and hypothesized determinants of practice within implementation science,[57] little is known about which of the hypothesized determinants are causally related to implementation of evidence-based practices[58,59] because very few trials test mechanisms or the processes responsible for change.[60] Our analysis of implementation strategy mechanisms will be critical to understanding how the strategies work and key to future efforts to optimize the effectiveness and transportability of our approaches. We will also gather information on associated implementation strategy costs to inform national scale-up efforts.

CHARACTERISTICS OF THE STUDY POPULATION:

1. Target Population and Accrual:

- Setting: all participants will be recruited from one of a maximum 41 ***pediatric clinics*** within two Mental Health Research Network (MHRN) systems (14 pediatric clinics within the Henry Ford Health [HFH], formerly Henry Ford Health System [HFH] in Michigan; up to 27 pediatric clinics within Kaiser Permanente Colorado [KPCO] in Colorado). Both systems use the Epic Electronic Health Record (EHR) system.

- Parents of youth seen in pediatric primary care (parents). We will include parents and/or legal guardians (referred to as parents going forward) at participating pediatric clinics who have a child aged 5-17 who attends a well visit. Please see the table below for our target enrollment numbers based on Research Activity and Study Period.
  - For the EHR/Administrative data extraction (i.e., which includes data from the EHR, VDW, and Administrative systems; see ***Data Collection*** section for more details), we anticipate HFH and KPCO to pull data from 74,080 unique clinic encounters each year (based on health system numbers from 2018). As such, we anticipate extracting data from about 37,040 clinic encounters in the 6-month pre-implementation period, 74,080 clinic encounters in the 1-year implementation period, and 74,080 clinic encounters in the 1-year sustainment period. In order to make this number we will pull approximately 100,000 from KPCO and 100,000 from HFH.
  - For the parent surveys, we will recruit eligible participants from the extracted EHR/Administrative data each study period. We estimate a 31.58% response rate, with our main goal of 23,394 surveys during the 1-year implementation period. We also hope to conduct 11,697 surveys in the 6-month pre-implementation period and 23,394 surveys in the 1-year sustainment period.

Study Phase	Pre-implementation (6 mo)	Implementation (1 yr)	Sustainment (1 yr)	Total maximum enrollment
EHR/Admin data	37,040	74,080	74,080	185,200[1]
Survey data (31.58% response rate)[2]	11,697	23,394	23,394	58,485[1]

1This is the total maximum enrollment (i.e. if all data came from unique patients at each period)

[2] The participants who complete the surveys will be identified from the EHR/Admin data above

- Primary Care Providers (PCPs). There are currently 137 pediatric physicians and 14 non-physician PCPs (N = 151) in the two systems. Two surveys will be administered to these PCPs: one during the 6-month pre-implementation period (baseline survey) and one during the 1-year active implementation period (implementation survey). We expect response rates of 50% at each of these two time points, so our target accrual for surveys is ***151 PCPs***. We will also purposively sample 16 of these PCP survey respondents to participate in a one-time qualitative interview.
  - Equity Supplement. We plan to identify up to 12 clinicians who engaged in the pilot at either Henry Ford Health or Kaiser Permanente Colorado for qualitative interviews. The key attendees for our stakeholder meeting will include Dr. Beidas and KPCO and HFH Site PIs (N=3), facilitation leaders (N=6), and pediatric leadership (N=4). Note, we are not conceptualizing the stakeholder meeting as a research activity.

- Health System Leaders (leaders). There are currently 20 health system leaders (i.e., clinic, department chiefs, and health plan directors) in the two systems. We plan to complete 6 health system leader qualitative interviews for Aim 2 (i.e., our target accrual for these interviews is ***6 leaders)***.

- Clinic point people (point people). As part of S.A.F.E. Firearm implementation, we have identified a "point person" at each participating clinic to be the primary point of contact regarding S.A.F.E. Firearm (e.g., they help coordinate lock deliveries, the research team checks in with them to see if the clinic has any questions or issues related to S.A.F.E. Firearm). Clinic point people vary in their roles depending on the clinic – some are clinicians and others hold other roles in the clinic (e.g., administrator, nurse). Clinic point people will be invited to participate in Aim 2 qualitative interviews. Our target accrual for ***point people interviews is 16***.

2. Key Eligibility Criteria:

- Parents (Aims 1 and 3). Parents and/or legal guardians at participating pediatric clinics who have a child aged 5-17 years who attends a well visit are eligible to participate in surveys per Aims 1 and 3. At least one parent must attend that well visit to be eligible. The parent must be at least 18 years old to participate. Lastly, at HFH, parents who are not US residents or do not speak English will not be eligible to participate. There are no other exclusion criteria to optimize ecological validity. A small number of patients may be excluded who previously requested to not be outreached for research studies.

  To avoid erroneously making parents aware of their teen's confidential visit at KPCO, parents of teens whose well-visits are tagged with a confidential diagnostic code (Z02.9) or the administrative type indicator of "Confidential Accounts – Do Not Use Insurance" by the clinician will not be eligible to participate in the survey. (Only youth ages 15-17 can have confidential visits or confidential diagnostic codes in the EHR.) Youth under 18 at HFH always need parental permission for visits. The one exception is teen sexual health visits (which can be confidential), but these visits are not well-visits, and therefore we will not be contacting parents about them. Therefore, this issue of parents not being aware of their children's well-visits cannot happen at HFH.

- PCPs (Aims 1 and 2). Pediatric physicians and non-physician PCPs employed within the participating pediatric clinics are eligible to participate in surveys per Aims 1 and 2. To be eligible for an interview per Aim 2, PCPs must have participated in at least one of the surveys.
  - Equity Supplement. To participate in interviews, individuals must be clinicians who participated in piloting procedures at either Henry Ford Health or Kaiser Colorado.

- Leaders (Aim 2). Health system leaders (i.e., clinic, department chiefs, and health plan directors) employed within the 2 health systems are eligible to participate in an interview per Aim 2.

- Point people (Aim 2). Clinic point people employed within the 2 health systems are eligible to participate in an interview per Aim 2.

3. Subject Recruitment and Screening:

- Parents (Aims 1 and 3). Parents who attend a well visit with a child aged 5-17 years will be eligible to participate in surveys per Aims 1 and 3. See **Procedures** section below for details about recruitment and enrollment.

- <u>PCPs (Aims 1 and 2)</u>. Leadership within both health systems has agreed to deploy these implementation strategies at their respective health systems. Half of the clinics will be randomized to receive only the EHR implementation strategy (*Nudge*), and the other half will be randomized to receive the EHR implementation strategy plus 1 year of facilitation to target additional clinician and organizational implementation barriers (*Nudge+*). All PCPs employed within the 41 pediatric clinics will receive either the *Nudge* or the *Nudge+*, and as such they will all be eligible to complete the baseline and active implementation surveys (per Aims 1 and 2). They will also be eligible to participate in an interview per Aim 2. See **Procedures** section below for details about recruitment and enrollment.
    - <u>Equity Supplemen</u>t. We will partner with each site's (KPCO, HFH) principal investigators (PIs) to recruit interview participants and will send invitations by email.

- <u>Leaders and Clinic Point People (Aim 2)</u>. All leaders and clinic point people will be eligible to participate in an interview per Aim 2. See **Procedures** section below for details about recruitment and enrollment.

4. Early Withdrawal of Subjects:
For Aim 1 (*Nudge* or *Nudge+*), we are requesting waiver of informed consent, so the option to early withdraw from this study is not applicable. For all surveys and interviews administered across aims, the steps to withdraw early from participation will be outlined during the consent process. See the **Consent Process** section below for more details.

5. Vulnerable Populations:
We are not targeting any vulnerable populations as part of this study.

6. Populations vulnerable to undue influence or coercion:
We will not be targeting participants who are likely to be vulnerable to undue influence or coercion.

STUDY DESIGN:
We will conduct a hybrid type III effectiveness-implementation trial with a longitudinal cluster randomized design to answer questions related to implementation strategy and program effectiveness. We will also apply mixed methods to investigate implementation strategy mechanisms. During the active implementation period, all 41 clinics in the two participating health systems will receive *SAFE Firearm* materials, including brief training in program delivery, cable locks, and the deployment of an EHR implementation strategy. Half of the clinics will be randomized to only receive the EHR implementation strategy (*Nudge*); the other half will be randomized to receive Nudge plus 1 year of facilitation to target additional clinician and organizational implementation barriers (*Nudge+*). We will randomize clinics to the active implementation conditions, as cluster randomization is more appropriate for organization-level implementation strategies[134] and because there is high potential for contamination if we were to randomize at the PCP level.[135] To ensure balance across conditions, we will use covariate-constrained randomization.[136] Covariates include health system (HFH, KPCO), clinic characteristics (size of clinic), and clinician characteristics (whether a stakeholder involved in study planning works in the clinic).

Based on historical precedent, we expect minimal staff turnover at the participating clinics during the study. If this does occur, we will follow procedures used in prior clinic-level cluster randomized trials, which involved brief training of new providers as part of their onboarding process at the clinic.

EQUITY SUPPLEMENT STUDY DESIGN:
Prior to launching the full trial, we are piloting the delivery of the *SAFE Firearm* program in 2 clinics in each health system during an approximately 4-5 month period. Pilot procedures will mirror the procedures described in the protocol and use the same parent survey instrument. After the pilot, we will conduct this sub-study. Specifically, we will explore any signals of disparities and examine *SAFE Firearm* delivery by children's medical complexity (per ICD-9 and -10 codes), sex, and race/ethnicity. *SAFE Firearm* delivery will be captured by the post-visit parent survey described in this protocol (below; see Parent Surveys [Aims 1 and 3]), which will be confirming receipt of the program; consistent with the primary outcome measure for the ASPIRE trial (as described in this protocol), clinicians must report "yes" to both program components (i.e., counseling and cable lock offer) to have received the program. Medical complexity will be determined by ICD-9 and -10 codes in the EHR, and race/ethnicity also be from the EHR (as described in the Data Collection section of this protocol). We will fit two separate inverse

probability weighted GEE logistic regression models to analyze the data. We will use a parameter of 10% difference in program delivery for youth in each group of interest to explore further. Regardless of whether we detect differences in program delivery, we will conduct qualitative interviews with up to 12 participants from Henry Ford Health or Kaiser Permanente Colorado to identify barriers to *SAFE Firearm* delivery. We will use the Health Equity Implementation Framework (HEIF) as a theoretical framework for the interviews, analysis, and interpretation of findings. The HEIF is a multilevel framework that assesses health equity determinants to identify factors that promote or hinder implementation of new interventions. The HEIF is well-suited for this inquiry given its explicit attention to the clinical encounter in which the intervention is delivered and emphasis on facilitation as essential process to address implementation problems, specifically disparities. Following analysis of the interview data, the research team will identify strategies to optimize facilitation.

As an extension of the Equity Supplement, we will repeat the above-described statistical analysis to descriptively evaluate approximately 6 months of data (i.e., from approximately March 2022-September 2022) in the same pilot clinics (N=4) to confirm the preliminary findings from the pre-trial pilot study. If we find a 10% difference in program delivery (as indicated above to signify clinical significance) by patients' race/ethnicity and sex, the trial facilitators (local members of the research team at Henry Ford and Kaiser CO) will feed back this information to pilot clinic clinicians, with the goal of increasing clinician awareness of potential disparities in implementation and broadly learning from this feedback process. If we do not find any clinically significant differences in program delivery, the facilitators will notify these clinics of the positive findings. These activities fall under the types of facilitation activities that the trial facilitators are already doing at clinics participating in the trial. For the Equity Supplement, we are simply proposing to do these same activities with the pilot clinics at both sites. We do not expect greater than minimal risks.

Study Duration:
We propose to conduct this project from 08/01/2020 through 06/30/2025, with human subject research activities starting no earlier than 01/01/2021 and data collection ending no later than 12/31/2024. After piloting procedures (planned for approximately April-May 2021 for parent survey pilot, and Fall 2021 and early 2022 [approximately October-February; duration of approximately 4-5 months] for SAFE Firearm program pilot), we plan to start in July 2021 to collect data across all aims during a 6-month pre-implementation period, a 1-year implementation period, and a 1-year sustainment period. We anticipate using the end of the project time to conduct analysis and prepare manuscripts.

Equity Supplement Study Duration:
S.A.F.E. Firearm piloting procedures will occur in Fall 2021 and early 2022 and last approximately 4-5 months. We propose to conduct interviews from January 2022-February 2022 (approximately 6 weeks). We will re-analyze approximately 6 months of pilot clinic data in approximately late October 2022 (once IRB approval is received) and engage in facilitation activities shortly afterward as described above.

METHODS:

1. Study Instruments:

- <u>Parent surveys (Aims 1 and 3)</u>.
    - These surveys will be administered three times, during a 6-month pre-implementation period, a 1-year implementation period, and a 1-year sustainment period. The parent survey will consist of two parts:
    - *Part 1*: One-question fidelity survey. To maximize response rate, we will only ask one question of parents; we are prioritizing the fidelity (Aim 1) questions. Half of parents will receive each fidelity question (regarding the clinician discussing firearm storage with them or being offered a cable lock). The survey will be sent via a text message that the parent can respond directly to with their multiple-choice response. We will use Twilio and REDCap for this text messaging (consistent with how text messaging for REDCap surveys has been done for this and other studies in the health systems; both are approved for use in the HFH and KPCO health systems and are HIPAA compliant). Responses will not be stored by Twilio and will be directly stored in REDCap. We will also recruit parents to participate in the one-question survey via email and/or patient portal message. For both, we will send a survey link for the parents to complete the survey in REDCap. Finally, we will offer participants the option to respond to the one-question survey directly over email. For this method, the survey question will be sent in the body of the email and participants will be instructed to reply via email with the number corresponding with their response.

Language alerting patients not to send confidential or identifying information in their email response will be included with the email survey message.

- *Part 2*: Based on our learnings about response rates from piloting our recruitment procedures, we are pivoting to collect fidelity (secondary Aim 1 outcome; Part 1 survey) to maximize response rate. Then, we will collect other secondary outcomes via the longer survey (Part 2, described below), understanding that the response rate will be lower for this second survey. After recruiting parents for the one-question survey (Part 1), we will invite them to participate in the longer parent survey (Part 2). We anticipate Part 2 parent surveys will take about 1-3 minutes for parents to complete.
  - Aim 1 measures will ask questions about PCP fidelity to the two steps of the *SAFE Firearm* program (whichever question was not answered during Part 1; e.g. did your child's PCP talk to you about firearm safety, did your child's PCP offer you a cable lock?). Aim 1 measures may also include questions about acceptability of the *SAFE Firearm* program and parents' reactions to the firearm discussions with providers.
  - Aim 3 measures will ask questions about firearm storage behavior (e.g. if they took a cable lock, if all firearms are stored with a gun lock on them, if bullets are stored separate from all firearms, etc.).
  - For NIH reporting purposes, we will also collect parent demographics (e.g., gender, race, ethnicity); disclosure of this information will be optional.

- PCP surveys (Aims 1 and 2). We anticipate PCP surveys will take about 15 minutes for PCPs to complete. Two surveys will be administered: one will be completed during the 6-month pre-implementation period (baseline survey) and one will be completed during the 1-year active implementation period. Each of the surveys will include questions related to some or all of the following constructs below:
  - Aim 1 measures will ask questions about acceptability and use of the *SAFE Firearm* program and of the implementation strategies. PCPs will also be offered the opportunity to disclose whether they are firearm owners; we will make it clear that disclosure is entirely voluntary.
  - Aim 2 moderator measures will ask questions about PCP attitudes and experiences (e.g., attitudes towards firearm counseling, burnout).
  - Aim 2 mediator measures will also ask about PCP attitudes and experiences (e.g., clinic-level adaptive reserve and PCP motivation to implement *SAFE Firearm*).
  - Demographic questions

- PCP, Point People, and Leader interviews (Aim 2). We anticipate interviews with PCPs, point people, and leaders will last about 15-30 minutes. These interviews will be administered once, during the end of active implementation through the early part of the sustainment period. Development of the interview guide was guided by the Consolidated Framework for Implementation Research (CFIR); the guide asks questions about other additional mechanisms through which implementation strategies might operate (e.g. clinician knowledge about firearms, community factors outside the clinic).
  - Equity Supplement. We will use a semi-structured interview guide to conduct interviews. The interview guide is based on the HEIF, with domains specific to recipients (patient and provider factors), clinical encounter, and organizational level. The interview guide will include several questions, e.g., "During this pilot period, how did you prioritize *SAFE Firearm* delivery?"; "How do you prioritize preventative counseling in the clinical encounter?"; "How did patients' medical needs impact *SAFE Firearm* delivery?" The guide will be structured to ensure consistent data collection and piloted.

2. Administration of Surveys and/or Process:

- Parent surveys (Aims 1 and 3). Parents will be asked to complete surveys up to 2 weeks following their completed well child visit. Parents will be contacted for the survey based on which contact method(s) are preferred and have been shown to work in previous research at each health system. For Part 1 (one-question survey), parents will be asked the survey question directly via text message and/or email (and they will respond with their multiple-choice answer) and/or will be invited via email and/or patient portal message to access the one-question survey via a REDCap link. For Part 2 (longer parent survey; after the Part 1 survey), parents will receive a message that will invite them to complete a brief survey via KPCO/HFH REDCap, a secure, web-based application for collecting and managing survey data that can be completed via computer or mobile device. Parents will be contacted via phone, email, text message, online patient portal (e.g., My Chart) message, and/or mail, sent by research specialists employed by their respective health system. We will contact parents

using these various methods for Parts 1 and 2 over the course of approximately 3-6 weeks. We will stop contacting parents after they have completed the survey, if they opt-out of subsequent recruitment, or once approximately 4-6 weeks have passed since their well visit. Participants contacted by phone will have the option to complete the survey verbally over the phone and participants contacted by mail will have the option to complete a mailed version of the survey. For Part 1, parents will not be compensated for participating. For Part 2, participants will be eligible for an incentive via lottery for survey completion (e.g., $100 gift card). Note that parents who receive the Part 2 survey via mail to complete and return to us will also be sent a consent form to review along with their completed survey. Participants will be informed that they should not complete the survey until they have read the consent form. Parents who receive the Part 1 survey via text message will be sent a link to the consent form in the introductory text message before the survey question. For people participating in Part 1 via email or patient portal message, a link to the consent form will be included within the invitation message. The Part 1 survey consent form indicates that participants should review it before completing the survey, and completion of the survey indicates their consent to participate.

- <u>PCP surveys (Aims 1 and 2)</u>. PCPs will be asked to complete surveys 2 times: once during the pre-implementation period (baseline survey) and once during the implementation period (implementation survey). They will be contacted via phone and email by the PIs and/or research specialists from their respective health systems and by research specialists on the Penn and/or Northwestern team using the Dillman Tailored Design Method. We have used this method in prior survey research with clinical staff. Surveys will be administered via Penn's REDCap (baseline survey) and Northwestern's REDCap (implementation survey). If allowed by their respective systems, PCPs will receive $20 e-gift cards or an altruistic gift ($20 donation to a suicide prevention charity on their behalf) at each time point they complete a survey.

- <u>PCP, Point People, and Leader interviews (Aims 2)</u>. PCPs, point people, and leaders will be asked to engage in a brief qualitative interview. We will purposively sample 16 PCP survey respondents (equally distributed across health system and randomized arm), to obtain more detailed information about their perspectives on S.A.F.E. Firearm. We will oversample for PCPs who chose to self-report firearm ownership on the survey. Additionally, we will interview 16 point people (stratified by health system, randomization arm, and reach) and 6 leaders (stratified by health system). If allowed by their respective systems, PCPs, point people, and leaders will receive a $25 electronic gift card or health system-branded gift of equivalent value for interview participation. Interviews will be audio-recorded. Data from interviews will be stored in Northwestern REDCap and on HIPAA-compliant servers at Northwestern. Data will be shared with the research teams at KPCO, HFH, and Penn using HIPAA-compliant methods in accordance with the data use agreement between KPCO, HFH, Penn, and Northwestern.

    - <u>Equity Supplement</u>. A trained research coordinator or team member will administer the one-time qualitative interview to participants verbally over the phone, or through videoconferencing software if the participant prefers. The interview will be audio-recorded. Demographics and audio recordings will be entered into and stored on REDCap, and transcribed interview data will be loaded into Nvivo (see Data Management section below for more details). Based on what is allowed at each health system, participants will receive a $50 electronic gift card (or if no gift card is permitted for research study incentives by the health system, we will send a personalized thank you email). We anticipate the interview will take about 25-30 minutes to complete.

      If our analysis of approximately 6 months of data (i.e., from approximately March 2022-September 2022) in the same pilot clinics (N=4) involved in the pre-trial equity sub-study indicates clinically significant differences, trial facilitators at both health systems will conduct facilitation activities (i.e., audit and feedback) with the pilot clinics at both sites.

3. Data Management:

A Data Use Agreement will be established between University of Pennsylvania, Northwestern University, and the two health systems in partnership with Penn's Office of Research Services and the respective offices at the three other sites.

At the University of Pennsylvania, we will use secure, encrypted servers to host the data and conduct the analysis to minimize the risk of breach of data and confidentiality. The Penn Medicine Academic Computing Services

(PMACS) will be the hub for the hardware and database infrastructure that will support the project. The PMACS provides a secure computing environment for a large volume of highly sensitive data, including clinical, genetic, socioeconomic, and financial information. PMACS requires all users of data or applications on PMACS servers to complete a PMACS-hosted cybersecurity awareness course annually, which stresses federal data security policies under data use agreements with the university. The curriculum includes Health Insurance Portability and Accountability Act (HIPAA) training and covers secure data transfer, passwords, computer security habits and knowledge of what constitutes misuse or inappropriate use of the server. We will implement multiple, redundant protective measures to guarantee the privacy and security of the participant data. All investigators and research staff with direct access to the identifiable data will be required to undergo annual responsible conduct of research, cybersecurity, and HIPAA certification in accordance with University of Pennsylvania regulations. Data will be stored, managed, and analyzed on a secure, encrypted server behind the University of Pennsylvania Health System (UPHS) firewall. All study personnel that will use this data are listed on the Penn IRB application and have completed training in HIPAA standards and the Collaborative IRB Training Initiative (CITI) human subjects research. Data access will be password protected. Whenever possible, data will be de-identified for analysis.

> Equity Supplement Data Management:
> All data management will be overseen by Drs. Beidas and Linn. Quantitative data management for the pilot data is consistent with procedures documented here in the protocol. Demographic data from the qualitative interviews will be entered into Research Electronic Data Capture (REDCap), a HIPAA-compliant web-based survey platform which is supported by the Penn Medicine Academic Computing Services (PMACS). REDCap provides secure, web-based applications that are flexible enough to be used for a variety of types of research, provide an intuitive interface for users to enter data and have real time validation rules (with automated data type and range checks) at the time of entry. These systems offer easy data manipulation with audit trails for reporting, monitoring and querying patient records, as well as an automated export mechanism to common statistical packages (SPSS, SAS, Stata, R/S-Plus). REDCap servers are housed in a local data center at the University of Pennsylvania and all web-based information transmission is encrypted. REDCap was developed specifically around HIPAA Security guidelines.

Audio recordings from the interviews will be digitally recorded and stored in REDCap. Audio recordings will be sent to TranscribeMe, a professional transcription service. TranscribeMe services include multiple safeguards designed to protect the privacy and security of personal health information, along with utilizing workers specifically cleared to work with this type of sensitive information. TranscribeMe maintains crowd worker teams that are vetted, trained, and authorized to work on content containing PHI/PII. Data submitted to TranscribeMe is stored on servers located inside secure, dedicated Microsoft Azure data centers, with state-of-the-art physical and online intrusion prevention measures in place. Data is submitted and maintained through a secure file transfer protocol (SFTP) platform that has been set up specifically for HIPAA compliance. The service limits the amount of internal staff that has access to customer data within this SFTP only to essential personnel. Transcripts will be loaded into Nvivo qualitative data analysis software for management and analysis. Qualitative analysis is described in the Statistical Analysis section below.

A number of procedures will be utilized to ensure confidentiality of participant data. First, all qualitative interview participants will be assigned a random ID number. This ID number will be used on all data collected from participants. The names that correlate to those ID numbers will be kept separate, i.e. identifiable data will be stored in one file and de-identified research data will be kept in a separate file. Given COVID-19 and the geographic spread of our participants (e.g., Colorado, Michigan), this study is designed to be deployed remotely and virtually. The key linking ID numbers to participant names will only be kept in REDCap. Only the research team outlined in this application will have access to the participant's identifiable data. Electronic records (e.g., digital audio files) will be stored in REDCap as well as on a PMACS and/or Northwestern HIPAA-compliant server. All requests to use the data will be reviewed by Dr. Beidas. Any data files provided to other individuals will be de-identified and contain only the random ID numbers. Participants will be notified of the above procedures during informed consent.

4. Management of Information for Multi-Site Research where a Penn Investigator is the Lead Investigator of a Multi-Site Study:
Dr. Kristin Linn (Penn) is the lead investigator at Penn of this multi-site study. All data collected from the two health systems will be securely transferred to Penn and Northwestern, where the Penn and Northwestern teams will work collaboratively to manage and analyze the data. Data collected at Northwestern will also be securely

transferred to Penn for management and analysis. All reporting will be in line with the Penn IRB's reporting requirement of unanticipated problems involving risks to participants or others. The two health systems (KPCO, HFH) and Northwestern have requested and received from their IRBs approval to cede to the Penn IRB which serves as the Single IRB for this project. Dr. Rinad Beidas, the original lead investigator at Penn, has moved to Northwestern University, so Penn IRB is also being asked to serve as the sIRB of this project on behalf of Northwestern. We have outlined below each institution involved in this project and their respective responsibilities:

- *University of Pennsylvania (Penn)*. The project team at Penn will provide scientific and administrative oversight of the proposed project and all project-related activities coordinated by the investigative teams at each site. They will lead the training in facilitation at both sites and provide ongoing support to facilitators at each site. The Penn site will oversee the data analysis and interpretation of findings.
- *Northwestern University* (NU): The project team at NU will support the Penn site at providing scientific and administrative oversight of the proposed project and all project-related activities coordinated by the investigative teams at HFH and KPCO. They will also lead some quantitative and qualitative data collection (e.g., implementation clinician survey; clinician, point people, and leader qualitative interviews), management, and analysis. They will provide ongoing support as needed across all sites.
- *Henry Ford Health (HFH)*. The project team at HFH will oversee the following activities at the participating clinics within their health system: the dissemination of *SAFE Firearm* program materials to clinics, brief clinician training, the build of the Epic SmartLists, and facilitation provided to clinics randomized to the Nudge+ condition. They will also coordinate the collection of quantitative data from parent and clinician participants and ensure safe data storage using REDCap.
- *Kaiser Permanente Colorado (KPCO)*. The project team at KPCO will oversee the following activities at the participating clinics within their health system: the dissemination of *SAFE Firearm* program materials to clinics, brief clinician training, the build of the Epic SmartLists, and facilitation provided to clinics randomized to the Nudge+ condition. They will also coordinate the collection of quantitative data from parent participants and ensure safe data storage using KPCO's REDCap. They will coordinate with Penn and Northwestern University for quantitative data collection (surveys) from PCPs which will be stored in Penn's REDCap and qualitative (interview) data collection from PCPs and health system leaders.

5. Subject Follow-up:
If it is decided that we will follow-up with participants, we will submit a modification and outline our reconsenting plan as necessary.

STUDY PROCEDURES:

1. Detailed Description:
Program and Implementation Strategies. Leadership within both health systems has agreed to deploy implementation strategies at their respective health systems, including the *SAFE Firearm* program, *Nudge* implementation strategy (EHR SmartList), and *Nudge+* implementation strategy (EHR SmartList plus facilitation) and thus our assessment is that they are ***not human subject research activities***. As a reminder, half of the clinics will be randomized to receive the *Nudge* implementation strategy and the other half will receive the *Nudge+* implementation strategy. We have provided a brief description of the program and two implementation strategies below for context.

- *SAFE Firearm program*. *SAFE Firearm* is an adapted version of *Safety Check*, which included: (a) screening for firearms; (b) brief counseling based on a motivational interviewing approach; and (c) offering cable locks. Our adapted program maintains the mechanisms of the original *Safety Check* program (i.e., counseling and offering a cable lock) but incorporates stakeholder feedback and principles from behavioral economics to further enhance the program. *SAFE Firearm* will include: (a) brief PCP counseling provided to all parents (without screening or documentation of ownership in the EHR) using motivational interviewing about safe firearm storage; and b) offering cable locks to all parents.
- *Nudge*. Clinics randomized to the *Nudge* implementation strategy will receive the EHR SmartList only. During the study's preparation phase, the project teams at HFH and KPCO will work with their respective pediatric clinic leadership and Epic information technology specialists to refine the design and functioning of our EHR SmartList, and to prototype and pilot it to ensure it is consistent with current workflow to be as unobtrusive as possible, consistent with typical practices in the respective health systems. SmartLists are pre-defined lists of

choices that users can select using their mouse or keyboard and are particularly helpful for documenting values that a PCP is required to use repeatedly, thus saving time and keyboard strokes. We will add a SmartList to the standard Well Child Visit workflow to serve as a reminder and allow for tracking of *SAFE Firearm* implementation. For example, the PCP may be required to select a value from a drop-down list to indicate whether they provided the SAFE Firearm program to the family or offered a cable lock. This Smart List will remain turned "on" during the implementation period through the sustainment period. Clinics will be responsible for making their own decisions about storage and distribution of firearm locks.

- *Nudge+*. Clinics randomized to the *Nudge+* implementation strategy will receive the *Nudge* implementation strategy (as described above) as well as facilitation. Facilitation is external support delivered by health system employees not employed within the clinic site. It will be offered for 12 months to each clinic, in keeping with other implementation trials. We will use a train-the-trainer model to train facilitators at HFH and KPCO to ensure they achieve facilitator core competencies with an eye toward implementation of *SAFE Firearm*. This may also involve sending key facilitators to a training around how to successfully implement facilitation. The role of the facilitator is to engage with study clinics, to assist each clinic in setting change and performance goals around the implementation of *SAFE Firearm*, and to troubleshoot implementation barriers. Our approach to facilitation was informed by established facilitation manuals (i.e., Veteran Health Affairs QUERI facilitation manual and AHRQ practice facilitation manual) and includes six stages. First, facilitators will engage in a pre-implementation readiness assessment for each clinic to identify potential implementation barriers and to develop relationships with stakeholders. Second, facilitators will hold kick-off meetings that signal the formal launch of the implementation strategy activities that include identifying where in the workflow *SAFE Firearm* can be implemented. This includes when *SAFE Firearm* will be delivered during the well-child visit, who in the clinic will be responsible for storing the cable locks, where the locks will be stored, and other workflow-related matters. Third, in the first 3 months of the active implementation period, facilitators will work with clinics around goal setting and metrics related to *SAFE Firearm*. During this period, the facilitator will conduct onsite/virtual visits to engage with practice leadership and PCPs once a month. In this phase, facilitators will also start to develop a sustainment plan in collaboration with stakeholders. Fourth, in months 3-9, the facilitators will engage in active implementation efforts based upon barriers assessed in the pre-implementation readiness phase as well as barriers that emerge as PCPs and clinics begin implementing. This includes established implementation strategies such as Plan-Do-Study-Act cycles and audit and feedback. All activities will be tracked via established logs to ensure the ability to understand which strategies are delivered and for implementation fidelity purposes. Fifth, in months 9-12, facilitators will engage in continued efforts to maintain gains and begin to enact the sustainment plan developed in the kick-off meeting. Sixth, in month 12, facilitation activities will complete and the clinics will transition to the formal maintenance period. Throughout the course of the active implementation period, facilitators will offer expert consultation (i.e., 2 webinars and technical assistance via email and phone as needed); and regular peer-to-peer calls supported by facilitators where clinics can share their experience.

**Timeline**	**Year 1**				**Year 2**				**Year 3**				**Year 4**				**Year 5**			
**Quarter**	**1**	**2**	**3**	**4**	**1**	**2**	**3**	**4**	**1**	**2**	**3**	**4**	**1**	**2**	**3**	**4**	**1**	**2**	**3**	**4**
Study planning	●	●	●	●																
PCP surveys (Baseline)					●	●														
PCP surveys (Implementation)									●											
Facilitation							●	●	●	●										
EHR Nudge							●	●	●	●	●	●	●	●						
Parent surveys (Aims 1 and 3)					●	●	●	●	●	●	●	●	●	●						
Lock Distribution & Cost Data (Aim 1)					●	●	●	●	●	●	●	●	●	●						
EHR/Admin Data collected (Aims 1 & 3)					●	●	●	●	●	●	●	●	●	●						
PCP, Point People, and Leader interviews (Aim 2)										●	●	●	●	●						
Blue refers to the pre-implementation period; green refers to the active implementation period; gray refers to the sustainment period																				

The above Timeline outlines the procedures and project periods. As described in the **Target Population and Accrual** section above, participants will include parents, PCPs, and leaders within 41 pediatric clinics across two health systems (HFH and KPCO). Parents will be asked to complete surveys 1-week following their completed well child visit to collect fidelity and acceptability (both Aim 1) and firearm storage behavior (Aim 3). PCPs will be asked to complete surveys at two time points (Aims 1 and 2): pre-implementation (baseline survey) and during active implementation (implementation survey). Logs will be collected from clinic staff to collect clinic-level data related to cable lock distribution and cost (Aim 1) and EHR data will be collected to measure reach (Aim 1) and youth suicide attempts, deaths, and unintentional firearm injury and mortality (Aim 3). We will conduct qualitative interviews during the end of active implementation period and the early part of the sustainment period with a subset of PCPs and point people and leaders (Aim 2).

Equity Supplement Procedures: The Penn interview team will work with the ASPIRE Site PIs at Henry Ford Heath System and Kaiser Permanente Colorado to identify potential participants who engaged in piloting procedures. The site PIs and/or Penn team will reach out via email with an invitation to participate. If clinicians are interested in participating, the interview team will schedule a date and time that is convenient to conduct a qualitative interview by phone or videoconferencing if the participant prefers. During the call but before the interview begins, the interview team will obtain consent (see Informed Consent section below for more details). The interview will not take place if the participant does not provide informed consent. The interview will be audio-recorded. Based on what is allowed at each health system, participants will receive a $50 electronic gift card (or if no gift card is permitted for research study incentives by the health system, we will send a personalized thank you email). After analyzing the interviews, we will use existing, ongoing meetings with clinical stakeholders to gather feedback on our findings. We will present our findings, which will allow stakeholders to select specific strategies (e.g., collaborative problem-solving) within facilitation to target disparities in SAFE Firearm delivery. We expect that this step will prime facilitation for trial launch and increase the likelihood of equitable outcomes.

2. Data Collection:

- Quantitative Survey Data and Qualitative Interview Data. The research teams at HFH and KPCO will work with their respective health systems to obtain necessary contact information to complete data collection. KPCO and HFH will outreach their respective health system parents for quantitative (survey) data collection by phone, email, text message, electronic patient portal (e.g., My Chart), and/or mail to recruit them to participate in quantitative surveys with outreach frequency consistent with their health system policy. The research team at Penn and Northwestern will work with the research teams at HFH and KPCO to contact PCPs, point people, and Leaders at both health systems by phone or email to invite them to participate in quantitative data collection (surveys) and qualitative interviews. During all recruitment activities with all potential participants, the research teams will make clear that participation in the research project is 100% voluntary and not mandatory. We will also ensure that leaders are aware that they cannot mandate that employees of their health system or that patients participate and that any involvement (or decision not to participate) in the proposed work will not have an adverse impact on their employment or care. All language used during the consenting process will thoroughly describe the procedures to be followed in the study. All language used during the consent process, surveys, and interview guides will be submitted to the oversight IRB prior to use. Surveys will collect information on clinician demographics. See **Administration of Surveys and/or Process** section above for additional details.

- Cost Data. Cost will be measured using a pragmatic method to capture all resources needed to deploy *Nudge* and *Nudge+* so that decision makers can have the information they need to take this approach to scale within their respective systems. Cost data will be collected from all clinics who are receiving the *SAFE Firearm* program (which, as a reminder, is not a human subject research activity and will be implemented across all clinics) using Excel templates collected from clinics on a monthly basis. These cost data will not include any identifiable information about any research participants and thus we believe the collection of this cost data is ***not human subject research***.

- EHR/Administrative Data (which includes data from the EHR, VDW, and Administrative Data). By virtue of being a part of the Mental Health Research Network (MHRN), both health systems deposit EHR and claims data into a virtual data warehouse (VDW). The VDW overcomes privacy concerns using a distributed data-model with standard variable definitions across systems, enabling research on large datasets without the need to share identifiable health information. We will collect the number of parent-youth ads who receive the program

from the VDW (i.e., reach). As part of this project, we will also utilize child medical records, claims, and administrative data extracted from the local VDW and EHR, as well as other existing health records (e.g., State Mortality records) and administrative databases. Although we expect our groups to be balanced with respect to patient and clinician characteristics, we may also extract the below data elements as relevant covariates at multiple time points over the course of the project. If we would like to collect data beyond the five years of this grant, we will communicate our plan to the IRB prior to collection.

- Healthcare Utilization: Inpatient, ER, and outpatient visits, procedures (identified using CPT codes) and diagnoses (identified using ICD codes) including but not limited to psychiatric diagnoses, diagnoses indicating probable or possible suicide attempt, injury or poisoning, and other medical diagnoses. Dates of utilization, procedure and diagnoses will also be collected. Any 42 CFR Part 2 covered data will be collected and used in compliance with those requirements
- Pharmacy Data: Ordered and filled prescriptions, including dates
- Patient Reported Outcomes Data: Patient reported outcome measures recorded in the EHR (e.g., PHQ, GAD-7)
- Enrollment: types of insurance coverage, dates of enrollment and unenrollment
- Demographics: age, sex, race/ethnicity, census block variables (e.g., income, educational attainment), patient language
- State or National Mortality Data: date and cause of death
- Well-visit firearm safe storage and cable lock documentation

3. Genetic Testing:
n/a

4. Use of Deception:
n/a

5. Statistical Analysis:

- Aim 1 Analysis. Our power analyses are based upon the primary implementation outcome of reach, consistent with best practices for hybrid type III trials.[61] All other implementation outcomes are secondary. Our sample sizes for the project are determined by the fixed number of parent-youth dyads, PCPs, and pediatric clinics within our two health systems. Data are clustered within health system, clinic, and PCP. We expect that approximately 48,475 parents of youth will be eligible to participate each year, and expect to include data from 151 PCPs, within 41 clinics. Therefore, we anticipate obtaining EHR reach data for 48,475 patients during the one-year active implementation period. We base our power calculations on our study design and prior research from our group demonstrating that current PCP use of the *SAFE Firearm* program components are 28% for brief counseling on safe firearm storage and 2% for offering cable locks.[14] We used the PASS power calculation program,[62] assuming an estimate of an ICC of .07 for explained variance among both PCPs and clinics,[63] and conclude that we will have 80% power to detect small effects of the less intensive implementation condition (*Nudge*) on reach (Cohen's d = 0.25 and 0.29, respectively).[64] Alpha is set at .05 (two-tailed). A small to moderate effect is reasonable to expect based on prior research demonstrating that EHR nudges using active choice approaches produce effects in this range.[51] Because the clinics will be our unit of randomization, it was not necessary to account for the health system ICC in the power analysis. We will account for any differences between health systems by weighting health system the strongest in our covariate constrained randomization. Also, we will conduct a sensitivity analysis that controls for health system as a fixed effect in our analyses and will explore whether intervention effectiveness varies significantly by health system. Our approach is consistent with similar work that has been published in leading medical journals (i.e., Navathe et al., 2019, *JAMA, Sharma et al., 2019, JAMA Oncology*). In Aim 1, the primary dependent variable is reach. For each observation period (active implementation, sustainment) and for each implementation condition (*Nudge, Nudge+*), we will describe the proportion of clinicians who reported having delivered the program with EHR reach. We will calculate EHR reach using three binary outcomes: received counseling (yes/no), offered lock (yes/no), both (yes/no). We will then conduct separate models. In the first set of models, we will examine whether the odds of receiving components of *SAFE Firearm* increased more in the *Nudge+* group compared with the *Nudge* group between the active implementation and sustainment periods. The fixed effects in the model will include implementation condition (*Nudge, Nudge+*), time (active implementation, sustainment), and their interaction; the magnitude and statistical significance of the parameter estimate for the interaction term will be of primary interest. Given the nested data (i.e., parent-youth dyads nested within PCPs; PCPs nested within clinics; clinics nested within

health systems), analyses will rely on 3-level logistic mixed effects regression models for reach as a dichotomous implementation outcome.[65,66] Linear mixed effects regression will be used to model continuous secondary outcomes (e.g., acceptability). For all models, our independent variable of interest will be a binary indicator of implementation condition assigned at the clinic level. All analyses will be conducted using MPlus to appropriately estimate multilevel regression models.[67] Although we expect our groups to be balanced with respect to patient and PCP characteristics, we will consider including the following variables, extracted from the VDW, as relevant covariates: health system (HFH, KPCO); youth demographics (age, gender, race and ethnicity, patient language); PCP demographics (specialty/degree, gender, year graduated from medical school); and public census data (socioeconomic indicators for patients based on geocoded patient addresses). In sensitivity analyses, we will examine each set of models separately by health system to account for structural differences in how care is delivered within the two systems. Sex and race/ethnicity, drawn from the EHR and survey, will be added to all models as fixed effects to account for the potential role they may play in implementation strategy and intervention effectiveness. We will also investigate whether sex moderates the effects of the implementation strategies. Given the potential high non-response to the parent survey following the visit, we will use inverse probability weighting to adjust for parental survey non-response (Skinner, 2011). Specifically, inverse probability weighting allows us to use patient, clinician, and clinic information from the EHR about both respondents and non-respondents to assign larger weights to survey responses from participants who are under-represented in the sample. For example, if we find that parents who are ethnic and/or racial minorities have a lower response rate than parents who are not ethnic and/or racial minorities, inverse probability weighting would give more weight in the analysis to survey responses from parents who are ethnic/racial minorities.

- Aim 2 Analysis. *Quantitative analysis.* For our 2-2-1 mediation analyses, we will have approximately 76 PCPs and 20 clinics per study arm. Using the PASS power calculation program,[62] we anticipate these sample sizes will yield sufficient power to detect a moderate indirect effect (Cohen's $d = .44$) through the clinic-level mediator (i.e., clinic adaptive reserve). We assume there will be a large effect of the strategy on the mediator and of the mediator on the outcome based on previous research.[68] This is a conservative estimate because the calculation does not account for the additional power that will be gained due to the two measurement time-points for each PCP. We will conduct exploratory analyses to investigate motivation as a mediator. Mediation will be tested using the product of coefficients approach for multilevel mediation analysis,[69-71] which we have used in previous studies.[72] In this approach, the total effect of the implementation strategy is parsed into direct and indirect effects associated with the mediator. We will test each mechanism in a separate 2-2-1 mediation model. Path "a" represents the effect of the implementation strategy (*Nudge, Nudge+*) at the clinic level on the clinic-level mediator (i.e., clinic adaptive reserve). Path "b" represents the relationship between the clinic-level mediator (i.e., adaptive reserve) and the three binary reach outcomes (counseling, cable lock, both). We will calculate change in reach for each of the two time-points using the 6 month period preceding each survey administration. An unbiased estimate of the mediated effect is derived via the product of the "a" and "b" paths (i.e., a*b) from these analyses.[70,71,73] We will test the statistical significance of the mediated effects using the joint significance test.[74] We will use Monte Carlo simulation methods to derive confidence intervals.[74-77] We will repeat this model for our exploratory mediator, motivation. Moderators of the strategies' effects will be tested separately by adding terms for each moderator and its interaction with the implementation strategy to the Aim 1 models only during the active implementation period. These models will estimate the conditional relationships between the implementation strategies and implementation outcomes across different values of the putative moderators. *Qualitative analysis and mixed methods.* Text answers from open-ended questions with parents from Aim 1, and digitally recorded and transcribed interviews with PCPs, point people, and leaders on the mechanisms of the implementation strategies, will be loaded into NVivo qualitative data analysis software. Analysis will be led by the research team guided by an integrated approach,[78] which outlines a rigorous, systematic method for analyzing qualitative data using an inductive and deductive process of iterative coding to identify recurrent themes, categories, and relationships. We will ensure at least 80% interrater reliability amongst our coders (we will have at least two). The structure of our mixed methods approach is sequential (quantitative data is collected before qualitative data and quantitative data is weighed more strongly than qualitative; QUAN>qual). The function is of complementarity (to elaborate upon the quantitative findings to understand the *how* of implementation), and the process is connecting (having the qualitative data set build upon the quantitative data set).[79] To integrate the quantitative and qualitative results, we will follow guidelines for best practices in mixed methods[80] as well as our previous experiences with mixed methods implementation research.[81-84]

- Aim 3 Analysis. We do not include a power analysis because in a hybrid type III effectiveness-implementation trial, power is based on implementation outcomes.[61] We note that we will not be powered to detect reductions in youth suicide attempts, deaths, and unintentional firearm injury and mortality given very low prevalence. However, we will conduct exploratory analyses using the same modeling techniques described in Aim 1 to assess the impact of the implementation conditions on parent-reported firearm storage behavior, youth suicide attempts, deaths, and unintentional firearm injury.

- Equity supplement Analysis: For analysis of the pilot data, we will fit two separate inverse probability weighted GEE logistic regression models to analyze the data. We will use a parameter of 10% difference in program delivery for youth in either group of interest to explore further. For analysis of the clinician interviews, we will use rapid qualitative analytic techniques, specifically structured summaries and matrix displays. We will begin analysis by systematically condensing the data (i.e., transcripts, field notes) to create an inventory of the most contextually meaningful data segments and quotations for each data collection episode. We will develop a structured template with key domains (i.e., clinical encounter, patient factors) to standardize the capture of interview content and increase the accessibility of the data. After all interviews are transformed onto structured templates, we will then transfer the summary points into a matrix, which will expedite recognition of similarities, differences, and trends across participants. We will use the matrix to develop barriers for program delivery. We will repeat the statistical analysis to descriptively evaluate approximately 6 months of data (i.e., from approximately March 2022-September 2022) in the same pilot clinics (N=4) to confirm the preliminary findings from the pre-trial pilot study.

RISK/BENEFIT ASSESSMENT:

1. Risks:

There are minimal risks to participants in this trial. The *SAFE Firearm* program is being implemented at both health systems. The program could increase the time PCPs spend with parents discussing firearm storage. However, shared-decision making is a high priority and evidence-based practice for pediatric primary care and increased time on this topic is likely valuable. In prior studies, nudges in the EHR were associated with minimal burden on PCPs. There is a risk of breach of data and confidentiality, however we described the precautions in place to securely manage this data in the **Data Management** section of this protocol.

Equity Supplement Risks: Same as described above. Potential harms to participants are minimal and there are no known physical, financial, or legal risks to participating in the study. However, interview participants will be asked about their perspectives of the implementation of *SAFE Firearm* in their health system. Participants may feel temporarily uncomfortable while participating in the interview. Dr. Beidas is a licensed clinical psychologist with extensive experience conducting qualitative research in community settings, and she will be available to speak with any participants who feel unduly distressed and make appropriate referrals as needed. An additional potential risk is the potential for loss of confidentiality. Participants may disclose personal sensitive information during interviews. All information shared during interviews will be kept private and confidential except in the case that disclosure is required by law. An exception to confidentiality is if a participant were to report child abuse or neglect, or if they report significant suicidal or homicidal ideation or intent to the research team. Any information about child abuse or intent to harm self or others will be reported to authorities, as required by law. No identifiable information that participants provide will be shared with any individuals outside of the study procedures outlined in this protocol. During the consent process, potential participants will be told about the alternative to participation (i.e. to not participate), and that they can withdraw at any time without penalty and can request their data be destroyed by submitting a written request to the research team. Potential participants will also be told that their decision to participate will not affect services or employment. This information is also outlined in the consent form.

2. Benefits:

Parents, PCPs, point people, and leaders will receive no direct benefits from participating in this study. Participants who enroll in this trial will benefit from the knowledge that they are contributing in an important way to potentially furthering scientific knowledge about reducing firearm access and related suicide risk. Both health systems included in our study indicated that they would adopt this approach if we can demonstrate its effectiveness in this trial, suggesting the sustainability of the proposed work. Furthermore, the evidence and insights generated can be taken to scale in the MHRN which includes 14 healthcare systems which are closely integrated. The knowledge gained on

how to implement a program like *SAFE Firearm* could be applied to other pediatric primary care settings and implemented at other health systems.

Equity Supplement Benefits: Participants may find satisfaction in sharing their perspectives with our research team as their feedback will be used to inform our facilitation implementation strategy. Their feedback may help the research team better understand perspectives about the barriers to implementation. The ratio of risks to benefit is reasonable given the importance of the information to be gleaned by this research.

3. Subject Privacy:

Privacy will be given utmost consideration and is highly valued in the proposed research. No research activities involve any direct interaction with subjects that would pose risk to their privacy.

Equity Supplement Subject Privacy: Privacy will be given utmost consideration and is highly valued in the proposed research. Participants will complete the interview over the phone or by videoconferencing if the participant prefers, and so can elect where they take the phone call; the interview will be in a private location where no one can overhear the content.

4. Subject Confidentiality:

Confidentiality refers to the subject's understanding of, and agreement to, the ways in which identifiable information will be stored and shared.

How will confidentiality of data be maintained? Check all that apply.

- ☒ Paper-based records will be kept in a secure location and only be accessible to personnel involved in the study.
- ☒ Computer-based files will only be made available to personnel involved in the study through the use of access privileges and passwords.
- ☒ Prior to access to any study-related information, personnel will be required to sign statements agreeing to protect the security and confidentiality of identifiable information.
- ☒ Whenever feasible, identifiers will be removed from study-related information.
- ☒ A Certificate of Confidentiality will be obtained, because the research could place the subject at risk of criminal or civil liability or cause damage to the subject's financial standing, employability, or liability.
- ☒ A waiver of documentation of consent is being requested, because the only link between the subject and the study would be the consent document and the primary risk is a breach of confidentiality. (This is not an option for FDA-regulated research.)
- ☒ Precautions are in place to ensure the data is secure by using passwords and encryption, because the research involves web-based surveys.
- ☒ Audio and/or video recordings will be transcribed and then destroyed to eliminate audible identification of subjects.
- ☐ Other (specify):

To protect participant confidentiality, only the research team outlined in this application will have access to review identified research records. Confidentiality will be protected to the fullest extent allowable under the law. See the **Data Management** section for more details.

For data that needs to be transmitted, it will be done through a Penn-approved secure encrypted file transfer solution as is described in Penn IRB's Guidance on Electronic Data Protection Requirements for Research Involving the Use of PHI. Records will not be released without the participant's consent unless required by law (e.g., imminent risk of harm to self suspected) or court order. When results of the research are presented at scientific meetings or published, no identifying information will be included.

All identifiable data, including the master list linking identifiers to the ID number and recordings, will be destroyed in 2030, five years after the award period ends.

5. Protected Health Information

Full details regarding data sharing and data management regarding PHI and PII is outlined in the data sharing and management appendix.

6. Compensation:

- Parent surveys (Aims 1 and 3). Parent participants for Part 1 of the parent survey will not receive compensation. Parent participants for Part 2 will be eligible for an incentive (i.e., $100 e-gift card) via lottery for survey completion.
- PCP surveys (Aims 1 and 2). If allowed by their respective system, PCP participants will receive $20 e-gift cards or we will make an altruistic gift ($20 donation to a suicide prevention charity on their behalf) each time they complete a survey.
- PCP, point people, and leader interviews (Aim 2). If allowed by their respective system, PCP, point people, and leader participants will receive a $25 e-gift card or health system-branded gift of equivalent value for participating in an interview.
    - Equity supplement: Based on what is allowed at each health system, participants will receive a $50 electronic gift card (or if no gift card is permitted for research study incentives by the health system, we will send a personalized thank you email).

7. Data and Safety Monitoring:

Diligent data and safety monitoring will be conducted by the PI and research team at Penn, Northwestern, HFH, and KPCO throughout the conduct of this study. This monitoring plan includes tracking participant safety and demographics, monitoring the safety of data, and monitoring and appropriately reporting adverse event activity. The PI and appropriate co-investigators will review data collected to ensure that no study findings warrant immediate intervention. We believe this research poses no greater than *minimal risk* and have proposed a monitoring plan that reflects this risk level.

The Northwestern PI (Beidas) will be responsible for oversight of potential adverse events. An adverse event (AE) is any untoward medical occurrence in a subject during participation in the clinical study. We anticipate two potential types of AEs that could be directly related to study participation. These include distress experienced with regard to research participation and breach of confidentiality and privacy. With regard to the former, we have protocols in place that include conferring with the PI (who is a licensed clinical psychologist) or a staff psychologist. With regard to the latter, we have appropriate safeguards to reduce risk of breach of confidentiality and privacy. As we are not legally mandated by any child access prevention law to disclose or report firearm storage practices, we do not anticipate AEs related to participants sharing information about their firearm storage practices with our research team. Additionally, we will defer to the respective health systems' procedures and policies related to any suicidal thoughts and/or behaviors identified during the course of this project. We appreciate the sensitive topic of discussion, and are committed to taking the appropriate steps if any unanticipated problems are identified during the course of this project. Otherwise, any risks related to additional potential AEs are not expected because the program, implementation strategies, and assessments pose minimal risk to subjects.

All members of the research team who will be involved in the design and conduct of the study must receive education in human research subjects protection from a training program that is approved by a properly constituted independent Ethics Committee or Institutional Review Board. The PI will be responsible for ensuring project faculty and staff have the equipment and training required to protect privacy and confidentiality and will monitor and document that these individuals are properly certified. If new senior/key personnel and staff become involved in the research, documentation that they have received the required education will be included in the annual progress reports.

The Penn IRB will serve as the IRB of record for any external ethics review boards or IRBs applicable to researchers from other institutions who may have access to human research subjects identified data (i.e. HFH, KPCO, Northwestern).

DSMB. Additionally, we have convened a three-member DSMB from faculty outside of the four institutions involved in this project (Penn, Northwestern, HFH, KPCO). DSMB composition includes the following: (1) a health services researcher with expertise in health systems and collection of data through EHR, (2) a biostatistician with expertise in longitudinal analysis, field trials, and clinical trial data, (3) an implementation research expert and firearm researcher. The DSMB will be an independent group of experts charged with reviewing study data for data

quality and integrity, adherence to the protocol, participant safety, and study conduct and progress. They will also make determinations regarding study continuations, modifications, and suspensions/terminations. DSMB members will be independent from any professional or financial conflict of interest with the research project and/or study investigators. The DSMB will meet at least once yearly via phone conference calls for the duration of the project. The DSMB will elect a Chair to moderate the meetings. At the initial meeting, the DSMB will review and approve all study protocols before study initiation to ensure participant safety. Protocols will include formal procedures for reporting and tracking all adverse reactions to the NIH and IRBs; tracking progress in the study; and identifying any need for premature termination of the protocol. At subsequent meetings, the DSMB will be provided with summary study progress reports and adverse events. The DSMB will provide a summary report following each meeting. We will not require the DSMB to conduct interim analyses of data prior to the end of the study.

8. Investigator's Risk/Benefit Assessment:
This study presents minimal risk that is balanced by the potential benefits of the research to society. This assessment is consistent for the equity supplement.

INFORMED CONSENT:

1. Consent Process:
We are requesting a waiver of written documentation of consent (see below) from the Penn IRB for participants who agree to participate in the surveys (parents, PCPs) and/or interviews (PCPs, point people, leaders). Because surveys will be completed via REDCap or phone and interviews will be conducted remotely, we will not have an opportunity to collect a signed copy of the consent form. We will not collect signed consent forms from participants who complete the parent survey via mail; these participants will receive a consent form to review before completing the survey. In the consent form and the introductory letter that they receive in the mail with the survey, they will be instructed that their completion and returning of the survey will be considered their consent to participate in the research study.

For the REDCap, mailed, and phone surveys, the required elements of informed consent will be described to the participant and, if they agree to participate, participants will consent by agreeing and proceeding to the electronic survey, completing the survey and returning it to the research team by mail, or providing verbal consent for surveys completed over the phone. Only participants who agree will be able to proceed to the online/verbal survey. Participants who complete the mailed survey will be explicitly instructed in both the mailed letter that accompanies the survey and the consent form that their completion of the survey is considered their consent. Additionally, the letter specifies that they should not complete the survey before reviewing the consent form and deciding to participate. For participants who complete the survey over the phone, a member of our research team will conduct informed consent remotely. We will review the required elements of informed consent and answer any questions the potential participant might have. Potential participants will be encouraged to ask questions about the project. Participants will be provided with a copy of the IRB approved consent document for their records. If they agree to participate, the research team will document the consent process in REDCap.

For the interviews, a member of our research team will conduct informed consent remotely. We will review the required elements of informed consent and answer any questions the potential participant might have. Potential participants will be encouraged to ask questions about the project. Participants will be provided with a copy of the IRB approved consent document for their records. If they agree to participate, the interview team will document the consent process in REDCap.

All potential participants will be informed that participation is voluntary and provided with the information that they need to make an informed choice. We will provide contact information to all potential survey respondents in case they have any questions before, during, or after completing the survey. Potential interview participants will be asked if they have any questions and if they agree to participate before any research questions are asked. All participants will be informed that that their decision to participate or not will not impact their relationship (i.e., services or employment) with their health system.

For the equity supplement, we are also requesting a ***waiver of written documentation of consent*** from the IRB for participants who agree to participate in the qualitative interview. Due to the Covid-19 pandemic and the geographic spread of participants (e.g., Colorado, Michigan), we have decided to conduct these interviews by phone or

videoconference to minimize risk posed to participants and to our research team. Because these meetings will be conducted by phone or videoconference, we will not have an opportunity to collect a signed copy of the consent form. A member of our interview team will conduct informed consent for qualitative interview participants over the phone or videoconference. We will review the required elements of informed consent and answer any questions the potential participant might have. Potential participants will be encouraged to ask questions about the project. Participants will be offered a copy of the IRB approved consent form for their records. If they agree to participate, the interview team will document the consent process in REDCap. All potential qualitative interview participants will be asked to confirm that they understand the information provided, understand that participation is voluntary, and feel able to make an informed choice. All participants will be assured during the informed consent process that their decision to participate or not will not impact their relationship (i.e., services or employment) with their health system.

2. Waiver of Informed Consent:

- Waiver of Informed Consent. The trial is pragmatic in nature, assessing the impact of implementation strategies (i.e. *Nudge* vs. *Nudge+*) delivered to PCPs through minor adjustments to existing workflow delivered through the EHR. Changes to workflow are by necessity systematically applied to all PCPs within the practices. Further, these changes to workflow are being implemented as part of leadership commitment to deploying the SAFE Firearm program and EHR Nudges and Facilitation with data collection to allow for evaluation (i.e., *SAFE Firearm* will be considered standard of practice). Because PCPs will still be free to engage with parents as they see fit and the implementation strategies represent minimal risk to PCPs and parents, we have requested **a waiver of informed consent** for allowing EHR/Administrative data to be collected for Aims 1 and 3. This approach will also aid implementation integrity and generalizability by avoiding behavior changes related to observation (i.e., the Hawthorne effect). The PI has worked closely with the Nudge Unit in the past on similar pragmatic trials that have received this waiver of informed consent, and our study team has designed the study with their trials in mind. As such, we believe there are several reasons to justify why a waiver of informed consent is requested (and has been granted for similar studies at Penn). First, it is not feasible to consent every provider and parent. Second, if consent was obtained from providers and parents, they would know they were being studied and this could change their behavior. This could potentially disrupt the design of the study and make interpretation of the findings challenging. Third, clinicians are not forced to implement the *SAFE Firearm* program (which, as a reminder, is an evidence-based practice that is being implemented in both health systems) and parents are not forced to engage in a conversation with their provider and they are not forced to take a cable lock. In all aims, providers can choose to use their best clinical judgement when it comes to the *SAFE Firearm* program. This research involves no more than minimal risk to the participants and will not adversely impact the rights and welfare of participants. As described above, the waiver is essential to research as this research could not be practicably carried out without this waiver.

- Waiver of Documentation of Consent for Online/Mailed/Phone Surveys. All surveys completed by parents (Aims 1 and 3) and PCPs (Aims 1 and 2) will be completed via phone, mail, or electronic platforms, and thus we are requesting waiver of written documentation of consent from the IRB. The required elements of informed consent will be described to the participant and, if they agree to participate, participants will consent by agreeing and proceeding to the electronic/phone survey. Mailed survey participants will be instructed to review the consent form before completing the survey. Only participants who agree will be able to proceed to the online/phone survey. Mailed survey participants will be instructed not to complete the survey until they have reviewed the consent form and decide to participate. For phone surveys, the required elements of consent will be described to the potential participant at the beginning of the survey, before any research questions are asked. If the participant verbally agrees to be complete the survey, the research staff who is conducting the interview will document the consent process. This research activity presents no more than minimal risk of harm to subjects and involves no procedures for which written consent is normally required outside of the research context.

- Partial Waiver of Consent for Part 1 Parent Survey. For the one-question Part 1 parent survey, we will share a link to the consent form in the introductory message sent before the survey question text message or in the survey invitation sent via email or patient portal message. All the required elements of consent will be described to the participant. In the informed consent form, they will be instructed to review the consent form before completing the survey. The consent form will also indicate that their completion of the survey indicates their consent to participate in the research. This research activity presents no more than minimal risk of harm to

subjects and involves no procedures for which written consent is normally required outside of the research context.

- Waiver of Documentation of Consent for Interviews. All interviews will be completed remotely and audio-recorded. The required elements of consent will be described to the potential participant at the beginning of the interview, before any research questions are asked. If the interview participant verbally agrees to be interviewed, the research staff who is conducting the interview will document the consent process. This research activity presents no more than minimal risk of harm to subjects and involves no procedures for which written consent is normally required outside of the research context.

- For the equity supplement, we also request a ***waiver of written documentation of informed consent*** (as described above) for the qualitative interview since the research presents no more than minimal risk of harm to subjects and involves no procedures for which written consent is normally required outside of the research context. The required elements of informed consent will be described over the phone or videoconference before any study activities occur.

RESOURCES NECESSARY FOR HUMAN RESEARCH PROTECTION:
Adequate facilities are available at Penn, Northwestern, HFH, and KPCO. The members of the research team are outlined in the application and include the Penn and Northwestern PIs, research investigators, full time research staff, and part time undergraduate research assistants. The entire team will be overseen by the PIs (Beidas, Linn). All personnel will complete required training before being granted access to any identifying information. This includes training on confidentiality through the CITI course. All Penn personnel will also sign confidentiality statements. These confidentiality statements will specify the procedures for reporting unintentional breaches in confidentiality to the PIs. The confidentiality statements also specify that violations of participants' confidentiality, either unintentional or deliberate, may result in termination of hire. The PIs will conduct training with all research personnel regarding data, limits of confidentiality, maintaining confidentiality, and proper study procedures. Personnel from HFH,KPCO, and Northwestern sign similar confidentiality statements and undergo similar research training within their respective systems.

Protocol amendments

**Submission date**	**Change(s)**	**Details and rationale**
08 March 2022	Changing primary study outcome from parent-reported receipt of *S.A.F.E. Firearm* program (i.e., fidelity) to clinician-reported delivery of *S.A.F.E. Firearm* in the electronic health record (i.e., reach) and other minor protocol revisions ahead of trial launch.	The protocol was updated in this modification to reflect the change in primary outcome. A detailed description of the rationale for the change in primary study outcome is described below in the Statistical Analysis Plan Summary of changes.<br><br>Additionally, the protocol was updated in this modification to reflect final decisions and details for the study prior to trial launch:<br>- Final number of clinics participating in the trial (N = 30) after removal of clinics that no longer met eligibility criteria ahead of trial launch<br>- Covariates included in covariate-constrained randomization of study clinics<br>- Finalized measurement plan and measures to be included in a survey that collected secondary outcomes data from clinicians at participating study clinics<br>The Data Safety and Monitoring Board approved of the primary outcome change and the full study protocol on 02 March 2022. The study's National Institute of Mental Health Program Officer also approved of the primary outcome change on 02 March 2022. These approvals were received and this modification to the protocol was submitted (8 March 2022) to the University of Pennsylvania Single Institutional Review Board prior to the trial's launch on 14 March 2022.
22 April 2022	Update protocol details related to administrative supplement award activities and analyses.	This modification pertained to the administrative supplement to the award funding the main trial (R01 MH123491-02S1, PI Beidas). The administrative supplement procedures were included in the main trial's IRB protocol. No changes pertaining to the main trial protocol were made as part of this modification.
01 August 2022	Adding Northwestern University as a study site and updating the University of Pennsylvania Site PI listed in the study protocol.	The study's Principal Investigator, Rinad S Beidas, PhD, left the University of Pennsylvania and transitioned to a new position at Northwestern University on 01 September 2022. Thus, the University of Pennsylvania Site Principal Investigator was updated in the study protocol to study biostatistician Kristin A Linn, PhD. Northwestern University was also added as a relying site, as Dr. Beidas and her team continued to lead the study from Northwestern University. Finally, the protocol was updated to reflect that Dr. Beidas' team would be (a) recruiting participants for a second survey that collected secondary outcomes data from clinicians at participating study clinics and (b) conducting qualitative interviews with study constituents.
19 October 2022	Updating health system name in study protocol.	One of the participating health systems changed its name; the protocol was updated with this new name.
	Updating data sharing and management details after the PI transitioned to Northwestern University.	Data management protocols were updated to note that study data would be securely shared by the research team at the University of Pennsylvania to the research team at Northwestern University for data management and analysis, consistent with the terms detailed in an updated Data Transfer and Use Agreement that was executed between Northwestern

		University, the University of Pennsylvania, and the two participating health systems.
	Update protocol details related to administrative supplement award activities and analyses.	This modification pertained to the administrative supplement to the award funding the main trial (R01 MH123491-02S1, PI Beidas). The administrative supplement procedures were included in the main trial's IRB protocol. No changes pertaining to the main trial protocol were made as part of this modification.
13 December 2022	Updating protocol with finalized plans for qualitative interviews.	**This is the final approved study protocol; no further changes were made for the duration of the trial. The full document is above.**<br><br>Qualitative interviews were conducted with clinicians, clinic change agents, and health system leaders after the active trial year ended. These interviews asked constituents about their experiences of and perspectives on *S.A.F.E. Firearm* and the implementation strategies, as well as needs for sustaining *S.A.F.E. Firearm* in the clinics and health systems long-term. The study protocol was updated with the finalized sampling and interview plan in preparation for launching the interviews (which began in May 2023).

Initial approved protocol

Approved by the University of Pennsylvania Single IRB: 08 February 2022

Social and Behavioral Sciences Human Research Protocol

PRINCIPAL INVESTIGATOR:
Rinad Beidas, PhD
ADDRESS
PHONE; EMAIL ADDRESS

PROTOCOL TITLE: A comparative effectiveness trial of strategies to implement firearm safety promotion as a universal suicide prevention strategy in pediatric primary care

INTRODUCTION AND PURPOSE:
The number of young people dying by suicide continues to rise in the U.S.[1,2] The risk for suicide death is much greater when there is an unlocked and loaded firearm in the home.[3] Two lines of evidence indicate that pediatric primary care is an ideal setting for universal prevention to reduce firearm access and related suicide risk. First, most youth visit primary care each year.[4] One study found that 77% of youth who died by suicide had accessed primary care in the prior year, with nearly 40% attending a visit within 4 weeks of death.[5] Second, studies have found that programs deployed in primary care can increase safe firearm storage among parents.[6-9] The most rigorous study tested *Safety Check*, found that parents in the intervention group increased safe firearm storage compared to the control group.[6] These results led the American Academy of Pediatrics[10] and National Academy of Medicine[11] to recommend implementation of evidence-based safe firearm storage programs like *Safety Check,* but the core components of the program have yet to become routine clinical practice. Implementation research is urgently needed to realize the potential of this program,[12-15] especially given that a simulation study found that even a modest increase in safe firearm storage could prevent up to 32% of firearm-related deaths in youth due to both suicide and accidents.[16]

The current proposal builds on a previous NIMH-funded project (IRB number 824449) conducted in the Mental Health Research Network (MHRN), in which we interviewed 70 stakeholders, including pediatric clinicians, health system leaders, firearm safety experts, and parents who own firearms. We queried about needed adaptations to *Safety Check*, implementation barriers, and preferred implementation strategies guided by the Consolidated Framework for Implementation Research (CFIR), a leading implementation science framework.[17-19] Adaptation recommendations included renaming the program (now called *SAFE Firearm*), removing the formal screening step and any documentation of firearm ownership in the electronic health record (EHR), and offering brief counseling on safe storage and cable locks to all families.[17,18] The most frequent implementation strategy suggestion was to integrate the program into the EHR. Studies support the effectiveness and scalability of EHR approaches to make implementation of new practices more salient and part of workflow.[20-22] Specifically, based on principles of behavioral economics, a well-designed EHR order set can ‘nudge’ clinician behavior.[23] However, our interviews identified implementation barriers that may warrant additional strategies, including clinician comfort with the program and clinic workflow.[19] Facilitation is an evidence-based implementation approach that focuses on building organizational capacity for improvement and directly targets the unique implementation barriers experienced by each clinic.[24-26] We will answer: Is the less costly and scalable EHR-based ‘nudge’ powerful enough to increase implementation of SAFE Firearm or is more intensive and expensive facilitation needed to overcome implementation barriers?

We will conduct a hybrid type III effectiveness-implementation trial with a longitudinal cluster randomized design to answer questions related to implementation strategy and program effectiveness. We will also apply mixed methods to investigate implementation strategy mechanisms. During the active implementation period, all 32 clinics in the two participating health systems will receive *SAFE Firearm* materials, including brief training in program delivery, cable locks, and the deployment of an EHR implementation strategy. Half of the clinics will be randomized to only receive the EHR implementation strategy (*Nudge*); the other half will be randomized to receive Nudge plus 1 year of facilitation to target additional clinician and organizational implementation barriers (*Nudge+*). We will collect the primary implementation outcome for 6 months before exposure to condition and for 1 year following to

allow for comparisons between (a) the active and pre-implementation periods and (b) the active and sustainment periods. Results will guide future efforts to promote firearm safety as a universal suicide prevention strategy.

EQUITY SUPPLEMENT PURPOSE:

Prior to launching the full trial, we are piloting the delivery of the *SAFE Firearm* program in 2 clinics in each health system during a 1-month period. Pilot procedures will mirror the procedures described in the protocol and use the same parent survey instrument (more details are below). After the pilot, we will conduct this sub-study. Specifically, we will explore any signals of disparities and examine *SAFE Firearm* delivery by children's medical complexity (per ICD-9 and -10 codes) and race/ethnicity. Because we anticipate that clinicians will provide helpful contextualization for the program that will be informative for facilitation for the program and for the broader literature on firearm safety counseling, regardless of whether we detect differences in program delivery, we will interview KPCO and HFHS clinicians who participated in the pilot to identify specific delivery barriers, and convene key stakeholders (e.g., facilitation leaders, pediatric leadership) for virtual meetings. We expect that this step will prime facilitation for trial launch. The goal of this sub-study is to learn more about barriers to delivering the *SAFE Firearm* program equitably to all patients, regardless of medical complexity and race/ethnicity, and to optimize facilitation to address disparities.

An important note about what we are requesting the IRB to review as part of this protocol: Leadership within both health systems has agreed to deploy the Evidence-Based Program (i.e. SAFE Firearm) and the Implementation Strategies (i.e. EHR Nudges, Facilitation) within their respective systems. They also are in support of conducting the Research Activities (i.e. quantitative surveys, qualitative interviews, and EHR/Administrative data extraction) to learn more about the implementation of the SAFE Firearm program as well as about EHR Nudge and Facilitation strategies. We have described the SAFE Firearm program as well as the EHR Nudge and Facilitation strategies in this protocol for reference, but we are requesting IRB review and oversight of only the Research Activities. The SAFE Firearm program is evidence-based and the primary research question isn't about effectiveness of this program but rather about implementation. Parents may receive SAFE Firearm during a pediatric primary care appointment and not participate in the research survey and vice versa. Requiring consent from participants to receive the SAFE Firearm program, EHR Nudge, and/or facilitation would make this implementation research study impossible to conduct because it would create undue bias and be overly burdensome on health systems. The program and strategies are low-risk, evidence-based, and prevention oriented, and ultimately the decision to deliver the SAFE Firearm program is left to the individual clinicians for each family. We are looking to answer pragmatic questions about the implementation strategies in a healthcare setting, i.e. is the less costly and scalable EHR-based 'nudge' powerful enough to increase implementation of SAFE Firearm or is more intensive and expensive facilitation needed to overcome implementation barriers? The purpose of this study is not to establish SAFE Firearm as an effective program, because that has already been done, but rather to study which strategy improves clinician fidelity to the program in a healthcare setting (as reported by parents).

OBJECTIVES:

- Aim 1. Conduct a longitudinal cluster randomized trial to test the comparative effectiveness of two active implementation strategies, *Nudge* (EHR) vs. *Nudge+* (EHR + facilitation).
- Aim 2. To use mixed methods to identify implementation strategy moderators and mediators.
- Aim 3. To examine the effects of SAFE Firearm on patient clinical outcomes.

Primary outcome variables:

- Aim 1. Parent-reported PCP fidelity
- Aim 2. Staff-reported clinic adaptive reserve
- Aim 3. Parent-reported firearm storage behavior

Secondary/Other outcome variables (Moderators and mediators to be measured):

- Aim 1. Reach, cable lock distribution, acceptability, cost
- Aim 2.
  - *Moderators*: Clinic implementation climate and leadership, clinician attitudes to suicide prevention
  - *Mediators:* Clinician motivation, clinic adaptive reserve
- Aim 3. Youth suicide attempts, deaths, and unintentional firearm injury and mortality

BACKGROUND:
Youth suicide deaths are on the rise in the United States (U.S.). Firearms are a common and lethal method of suicide attempt. Reducing access to lethal means is a promising yet underused suicide prevention strategy.[27] Firearms are a critical target of such efforts [28,29] given that they are present in one in three U.S. homes and these numbers are increasing in the wake of Covid-19.[30] Recent research has found that only 3 in 10 firearm-owning families with children store all guns in their home in the safest manner: locked and unloaded. Approximately 4.6 million U.S. children (7%) live in homes in which at least one firearm is stored unlocked and loaded.[31] Given that the presence of firearms in the home is a robust risk factor for suicide,[32] safe storage of firearms in the home is imperative for reducing youth suicide attempts and death. Simulation research has found that even a modest increase in safe firearm storage could prevent as many as 32% of youth firearm deaths due to suicide and accidents.[16] Thus, efforts to increase implementation of interventions to improve safe storage could save young lives nationally.

The success of lethal means safety interventions in other settings highlights their potential value in primary care as a strategy to save young lives. Means safety interventions have been shown to be effective in specialty behavioral health,[33] emergency departments,[34] and in the community.[35] While these efforts are important, primary care represents a critical opportunity for universal suicide prevention, as it has long served as the de facto mental health services system.[36] For example, whereas only one third of youth receive mental health treatment in the year prior to a suicide attempt,[37,38] four out of five youth who die by suicide visit primary care during that time period.[37] Further, at least half of individuals who die by suicide with a firearm have no known psychiatric history,[39,40] underscoring the importance of universal prevention. With 90% of youth visiting primary care annually[41] and a high rate of primary care visits among youth who die by suicide in the year prior,[42] primary care provides an excellent opportunity for universal firearm safety intervention.

Safety Check is an evidence-based, pediatric primary care program targeting parental safe firearm storage as part of a bundle of violence prevention strategies.[6] The program includes screening for firearms in the home, brief counseling around safe storage, and providing cable locks. A large clinical trial in 137 pediatric practices found that parents receiving *Safety Check* reported double the odds of safe storage (OR = 2.0, $p < .011$) compared to the control group. The intervention group showed a 10% increase in use of cable locks, while there was a 12% decrease in safe storage in the control group ($p < .001$). While these results led major professional organizations to recommend implementation of safe firearm storage programs like *Safety Check*, they have not been routinely implemented.[14] There are a number of potential explanations for slow uptake. One potential reason may be the fit of the program for primary care (i.e., program-setting fit).[17] To investigate whether this might be the case and to increase our understanding of how best to implement *Safety Check* as a universal suicide prevention strategy, we conducted pre-implementation work in two health systems, guided by CFIR.[17] This work was particularly salient given that in the 15 years since the *Safety Check* program was designed, there have been advances in implementation science and an increasingly sensitive national debate about firearms, including an evolving role of professional medical associations in advocating for firearm safety as a public health approach. Our preliminary work allowed us to gather key information about barriers and facilitators to the implementation of *Safety Check* in primary care as a universal suicide prevention strategy within the current national context, including primary care provider (PCP) attitudes about discussing firearm safety with parents and firearm stakeholders' attitudes about PCPs raising this topic.[14,18] Recent work funded through a pilot grant from the FACTS Consortium, an NICHD-funded R24, has allowed us to incorporate stakeholder feedback from this previous work and additional pilot work to adapt *Safety Check* to increase its feasibility and acceptability. This has paved the way for adaptations to the program, now called *SAFE Firearm*, and the development of implementation strategies to be tested in the proposed trial.

Implementation strategies informed by stakeholder input and behavioral economics have the potential to increase use of *SAFE Firearm* in routine pediatric practice. Behavioral economics focuses on the power of context and an individual's limited resources (e.g., time, attention) in shaping decisions and behavior,[43] thus allowing for the identification of common, predictable cognitive heuristics or shortcuts that people use in making decisions.[44,45] These heuristics can be accommodated and harnessed through choice architecture, which involves changing the environment to facilitate the desired evidence-based choice.[46] One of the most powerful ways to use choice architecture to affect behavior is through nudges, which involve making subtle changes to the way that choices are presented.[47] These approaches have been leveraged to change clinician practice in multiple areas of medicine[20,48-50] and have yielded promising effects when applied to mental health in our NIMH-funded P50 Penn ALACRITY Center (MPIs: Beidas, Mandell, Buttenheim).[46] Nudges deployed through the electronic health record (EHR) have the capacity to prompt and shape medical decision-making in unobtrusive ways that do not disrupt workflow or add

more tasks and have the potential to be effective and low-cost.[20,22,51,52] Given that more than 90% of U.S. hospitals use an EHR[53] and that the highest rates of EHR adoption are in primary care,[54] EHR implementation strategies are also scalable. At the same time, our stakeholder research revealed that additional, more intensive implementation strategies may be needed to maximize implementation success, particularly within the context of a sensitive topic such as firearm safety.

The proposed research draws on multiple streams of evidence to maximize impact in the context of an urgent and sensitive topic and incorporates the latest advances in implementation science. Despite the promise, behavioral economics and implementation science approaches have not been systematically integrated and applied to suicide prevention. Merging these fields of study offers an opportunity to test the support needed for implementation of *SAFE Firearm* and will also provide unique insights into implementation of evidence-based practices in primary care more broadly. Testing these strategies in the context of a hybrid effectiveness-implementation trial will also aid in reducing youth suicide deaths. Additionally, despite the proliferation of conceptual frameworks[55,56] and hypothesized determinants of practice within implementation science,[57] little is known about which of the hypothesized determinants are causally related to implementation of evidence-based practices[58,59] because very few trials test mechanisms or the processes responsible for change.[60] Our analysis of implementation strategy mechanisms will be critical to understanding how the strategies work and key to future efforts to optimize the effectiveness and transportability of our approaches. We will also gather information on associated implementation strategy costs to inform national scale-up efforts.

CHARACTERISTICS OF THE STUDY POPULATION:

1. Target Population and Accrual:

- Setting: all participants will be recruited from one of a maximum 41 ***pediatric clinics*** within two Mental Health Research Network (MHRN) systems (14 pediatric clinics within the Henry Ford Health System [HFHS] in Michigan; up to 27 pediatric clinics within Kaiser Permanente Colorado [KPCO] in Colorado). Both systems use the Epic Electronic Health Record (EHR) system.

- Parents of youth seen in pediatric primary care (parents). We will include parents and/or legal guardians (referred to as parents going forward) at participating pediatric clinics who have a child aged 5-17 who attends a well visit. Please see the table below for our target enrollment numbers based on Research Activity and Study Period.
  - For the EHR/Administrative data extraction (i.e., which includes data from the EHR, VDW, and Administrative systems; see ***Data Collection*** section for more details), we anticipate HFHS and KPCO to pull data from 74,080 unique clinic encounters each year (based on health system numbers from 2018). As such, we anticipate extracting data from about 37,040 clinic encounters in the 6-month pre-implementation period, 74,080 clinic encounters in the 1-year implementation period, and 74,080 clinic encounters in the 1-year sustainment period. In order to make this number we will pull approximately 100,000 from KPCO and 100,000 from HFHS.
  - For the parent surveys, we will recruit eligible participants from the extracted EHR/Administrative data each study period. We estimate a 31.58% response rate, with our main goal of 23,394 surveys during the 1-year implementation period. We also hope to conduct 11,697 surveys in the 6-month pre-implementation period and 23,394 surveys in the 1-year sustainment period.

Study Phase	Pre-implementation (6 mo)	Implementation (1 yr)	Sustainment (1 yr)	Total maximum enrollment
EHR/Admin data	37,040	74,080	74,080	185,200[1]
Survey data (31.58% response rate)[2]	11,697	23,394	23,394	58,485[1]

1This is the total maximum enrollment (i.e. if all data came from unique patients at each period)
[2] The participants who complete the surveys will be identified from the EHR/Admin data above

- Primary Care Providers (PCPs). There are currently 137 pediatric physicians and 14 non-physician PCPs (N = 151) in the two systems. Two surveys will be administered to these PCPs: one during the 6-month pre-implementation period (baseline survey) and one during the 1-year active implementation period (implementation survey). We expect response rates of 50% at each of these two time points, so our target

accrual for surveys is ***151 PCPs***. We will also purposively sample 24 of these PCP survey respondents to participate in a one-time qualitative interview.

- Equity Supplement. We plan to identify up to 12 clinicians who engaged in the pilot at either Henry Ford Health System or Kaiser Permanente Colorado for qualitative interviews. The key attendees for our stakeholder meeting will include ASPIRE PI Beidas and Site PIs (N=3), facilitation leaders (N=6), and pediatric leadership (N=4). Note, we are not conceptualizing the stakeholder meeting as a research activity.

- Health System Leaders (leaders). There are currently 20 health system leaders (i.e., clinic, department chiefs, and health plan directors) in the two systems. We expect response rates of 70% for our health system leader qualitative interviews per Aim 2, so our target accrual for interviews is ***14 leaders***.

2. Key Eligibility Criteria:

- Parents (Aims 1 and 3). Parents and/or legal guardians at participating pediatric clinics who have a child aged 5-17 years who attends a well visit are eligible to participate in surveys per Aims 1 and 3. At least one parent must attend that well visit to be eligible. The parent must be at least 18 years old to participate. Lastly, at HFHS, parents who are not US residents or do not speak English will not be eligible to participate. There are no other exclusion criteria to optimize ecological validity. A small number of patients may be excluded who previously requested to not be outreached for research studies.

  To avoid erroneously making parents aware of their teen's confidential visit at KPCO, parents of teens whose well-visits are tagged with a confidential diagnostic code (Z02.9) or the administrative type indicator of "Confidential Accounts – Do Not Use Insurance" by the clinician will not be eligible to participate in the survey. (Only youth ages 15-17 can have confidential visits or confidential diagnostic codes in the EHR.) Youth under 18 at HFHS always need parental permission for visits. The one exception is teen sexual health visits (which can be confidential), but these visits are not well-visits, and therefore we will not be contacting parents about them. Therefore, this issue of parents not being aware of their children's well-visits cannot happen at HFHS.

- PCPs (Aims 1 and 2). Pediatric physicians and non-physician PCPs employed within the participating pediatric clinics are eligible to participate in surveys per Aims 1 and 2. To be eligible for an interview per Aim 2, PCPs must have participated in at least one of the surveys.
  - Equity Supplement. To participate in interviews, individuals must be clinicians who participated in piloting procedures at either Henry Ford Health System or Kaiser Colorado.

- Leaders (Aim 2). Health system leaders (i.e., clinic, department chiefs, and health plan directors) employed within the 2 health systems are eligible to participate in an interview per Aim 2.

3. Subject Recruitment and Screening:

- Parents (Aims 1 and 3). Parents who attend a well visit with a child aged 5-17 years will be eligible to participate in surveys per Aims 1 and 3. See **Procedures** section below for details about recruitment and enrollment.

- PCPs (Aims 1 and 2). Leadership within both health systems has agreed to deploy these implementation strategies at their respective health systems. Half of the clinics will be randomized to receive only the EHR implementation strategy (*Nudge*), and the other half will be randomized to receive the EHR implementation strategy plus 1 year of facilitation to target additional clinician and organizational implementation barriers (*Nudge+*). All PCPs employed within the 41 pediatric clinics will receive either the *Nudge* or the *Nudge+*, and as such they will all be eligible to complete the baseline and active implementation surveys (per Aims 1 and 2). They will also be eligible to participate in an interview per Aim 2. See **Procedures** section below for details about recruitment and enrollment.
  - Equity Supplement. We will partner with each site's principal investigators (PIs) to recruit interview participants and will send invitations by email.

- Leaders (Aim 2). All leaders will be eligible to participate in an interview per Aim 2. See **Procedures** section below for details about recruitment and enrollment.

4. Early Withdrawal of Subjects:
For Aim 1 (*Nudge* or *Nudge+*), we are requesting waiver of informed consent, so the option to early withdraw from this study is not applicable. For all surveys and interviews administered across aims, the steps to withdraw early from participation will be outlined during the consent process. See the **Consent Process** section below for more details.

5. Vulnerable Populations:
We are not targeting any vulnerable populations as part of this study.

6. Populations vulnerable to undue influence or coercion:
We will not be targeting participants who are likely to be vulnerable to undue influence or coercion.

STUDY DESIGN:
We will conduct a hybrid type III effectiveness-implementation trial with a longitudinal cluster randomized design to answer questions related to implementation strategy and program effectiveness. We will also apply mixed methods to investigate implementation strategy mechanisms. During the active implementation period, all 41 clinics in the two participating health systems will receive *SAFE Firearm* materials, including brief training in program delivery, cable locks, and the deployment of an EHR implementation strategy. Half of the clinics will be randomized to only receive the EHR implementation strategy (*Nudge*); the other half will be randomized to receive Nudge plus 1 year of facilitation to target additional clinician and organizational implementation barriers (*Nudge+*). We will randomize clinics to the active implementation conditions, as cluster randomization is more appropriate for organization-level implementation strategies[134] and because there is high potential for contamination if we were to randomize at the PCP level.[135] To ensure balance across conditions, we will use covariate-constrained randomization.[136] Covariates, weighted in order of importance, include health system (HFHS, KPCO), clinic characteristics (implementation climate, firearm activities already taking place, location [urban vs. non-urban]), and clinician characteristics (burnout).

Based on historical precedent, we expect minimal staff turnover at the participating clinics during the study. If this does occur, we will follow procedures used in prior clinic-level cluster randomized trials, which involved brief training of new providers as part of their onboarding process at the clinic.

EQUITY SUPPLEMENT STUDY DESIGN:
Prior to launching the full trial, we are piloting the delivery of the *SAFE Firearm* program in 2 clinics in each health system during a 1-month period. Pilot procedures will mirror the procedures described in the protocol and use the same parent survey instrument. After the pilot, we will conduct this sub-study. Specifically, we will explore any signals of disparities and examine *SAFE Firearm* delivery by children's medical complexity (per ICD-9 and -10 codes) and race/ethnicity. *SAFE Firearm* delivery will be captured by the post-visit parent survey described in this protocol (below; see Parent Surveys [Aims 1 and 3]), which will be confirming receipt of the program; consistent with the primary outcome measure for the ASPIRE trial (as described in this protocol), parents must report "yes" to both program components (i.e., counseling and cable lock offer) to have received the program. Medical complexity will be determined by ICD-9 and -10 codes in the EHR, and race/ethnicity also be from the EHR (as described in the Data Collection section of this protocol). We will fit two separate inverse probability weighted GEE logistic regression models to analyze the data. We will use a parameter of 10% difference in program delivery for youth in either group of interest to explore further. Regardless of whether we detect differences in program delivery, we will conduct qualitative interviews with up to 12 participants from Henry Ford Health System or Kaiser Permanente Colorado to identify barriers to *SAFE Firearm* delivery. We will use the Health Equity Implementation Framework (HEIF) as a theoretical framework for the interviews, analysis, and interpretation of findings. The HEIF is a multilevel framework that assesses health equity determinants to identify factors that promote or hinder implementation of new interventions. The HEIF is well-suited for this inquiry given its explicit attention to the clinical encounter in which the intervention is delivered and emphasis on facilitation as essential process to address implementation problems, specifically disparities. Following analysis of the interview data, we will convene key stakeholders (e.g., facilitation leaders, pediatric leadership) for virtual meetings to optimize facilitation to address disparities. We will use existing, ongoing meetings with clinical stakeholders to gather feedback. Note that we are not conceptualizing stakeholder meeting as a research activity.

Study Duration:

We propose to conduct this project from 08/01/2020 through 06/30/2025, with human subject research activities starting no earlier than 01/01/2021 and data collection ending no later than 12/31/2024. After piloting procedures (planned for approximately April-May 2021 for parent survey pilot, and Fall 2021 [approximately October; duration of 1 month] for SAFE Firearm program pilot), we plan to start in July 2021 to collect data across all aims during a 6-month pre-implementation period, a 1-year implementation period, and a 1-year sustainment period. We anticipate using the end of the project time to conduct analysis and prepare manuscripts.

Equity Supplement Study Duration:

S.A.F.E. Firearm piloting procedures will occur in Fall 2021 and last 1-month. We propose to conduct interviews from January 2022-February 2022 (approximately 6 weeks) and convene stakeholders during existing, ongoing virtual meetings in approximately February 2022.

METHODS:

1. Study Instruments:

- Parent surveys (Aims 1 and 3).
    - These surveys will be administered three times, during a 6-month pre-implementation period, a 1-year implementation period, and a 1-year sustainment period. The parent survey will consist of two parts:
    - *Part 1*: One-question fidelity survey. To maximize response rate, we will only ask one question of parents; we are prioritizing the fidelity (Aim 1) questions. Half of parents will receive each fidelity question (regarding the clinician discussing firearm storage with them or being offered a cable lock). The survey will be sent via a text message that the parent can respond directly to with their multiple-choice response. We will use Twilio and REDCap for this text messaging (consistent with how text messaging for REDCap surveys has been done for this and other studies in the health systems; both are approved for use in the HFHS and KPCO health systems and are HIPAA compliant). Responses will not be stored by Twilio and will be directly stored in REDCap. We will also recruit parents to participate in the one-question survey via email and/or patient portal message. For both, we will send a survey link for the parents to complete the survey in REDCap. Finally, we will offer participants the option to respond to the one-question survey directly over email. For this method, the survey question will be sent in the body of the email and participants will be instructed to reply via email with the number corresponding with their response. Language alerting patients not to send confidential or identifying information in their email response will be included with the email survey message.
    - *Part 2*: Based on our learnings about response rates from piloting our recruitment procedures, we are pivoting to collect our primary outcome (Part 1 survey) to maximize response rate. Then, we will collect other secondary outcomes via the longer survey (Part 2, described below), understanding that the response rate will be lower for this second survey. After recruiting parents for the one-question survey (Part 1), we will invite them to participate in the longer parent survey (Part 2). We anticipate Part 2 parent surveys will take about 1-3 minutes for parents to complete.
        - Aim 1 measures will ask questions about PCP fidelity to the two steps of the *SAFE Firearm* program (whichever question was not answered during Part 1; e.g. did your child's PCP talk to you about firearm safety, did your child's PCP offer you a cable lock?). Aim 1 measures may also include questions about acceptability of the *SAFE Firearm* program and parents' reactions to the firearm discussions with providers.
        - Aim 3 measures will ask questions about firearm storage behavior (e.g. if they took a cable lock, if all firearms are stored with a gun lock on them, if bullets are stored separate from all firearms, etc.).
        - For NIH reporting purposes, we will also collect parent demographics (e.g., gender, race, ethnicity); disclosure of this information will be optional.

- PCP surveys (Aims 1 and 2). We anticipate PCP surveys will take about 15 minutes for PCPs to complete. Two surveys will be administered: one will be completed during the 6-month pre-implementation period (baseline survey) and one will be completed during the 1-year active implementation period. Each of the surveys will include questions related to some or all of the following constructs below:
    - Aim 1 measures will ask questions about acceptability and use of the *SAFE Firearm* program and of the implementation strategies. PCPs will also be offered the opportunity to disclose whether they are firearm owners; we will make it clear that disclosure is entirely voluntary.

   - Aim 2 moderator measures will ask questions about PCP attitudes and experiences (e.g., attitudes towards firearm counseling, burnout).
   - Aim 2 mediator measures will also ask about PCP attitudes and experiences (e.g., clinic-level adaptive reserve and PCP motivation to implement *SAFE Firearm*).
   - Demographic questions

- PCP and Leader interviews (Aim 2). We anticipate interviews with PCPs and leaders will last about 30 minutes. These interviews will be administered once, during the sustainment period. Development of the interview guides will be guided by the Consolidated Framework for Implementation Research (CFIR) and will ask questions about other additional mechanisms through which implementation strategies might operate (e.g. clinician self-efficacy, psychological safety).
   - Equity Supplement. We will use a semi-structured interview guide to conduct interviews. The interview guide is based on the HEIF, with domains specific to recipients (patient and provider factors), clinical encounter, and organizational level. The interview guide will include several questions, e.g., “During this pilot period, how did you prioritize *SAFE Firearm* delivery?”; “How do you prioritize preventative counseling in the clinical encounter?”; “How did patients’ medical needs impact *SAFE Firearm* delivery?” The guide will be structured to ensure consistent data collection and piloted.

2. Administration of Surveys and/or Process:

- Parent surveys (Aims 1 and 3). Parents will be asked to complete surveys up to 2 weeks following their completed well child visit. Parents will be contacted for the survey based on which contact method(s) are preferred and have been shown to work in previous research at each health system. For Part 1 (one-question survey), parents will be asked the survey question directly via text message and/or email (and they will respond with their multiple-choice answer) and/or will be invited via email and/or patient portal message to access the one-question survey via a REDCap link. For Part 2 (longer parent survey; after the Part 1 survey), parents will receive a message that will invite them to complete a brief survey via KPCO/HFHS REDCap, a secure, web-based application for collecting and managing survey data that can be completed via computer or mobile device. Parents will be contacted via phone, email, text message, online patient portal (e.g., My Chart) message, and/or mail, sent by research specialists employed by their respective health system. We will contact parents using these various methods for Parts 1 and 2 over the course of approximately 3-6 weeks. We will stop contacting parents after they have completed the survey, if they opt-out of subsequent recruitment, or once approximately 4-6 weeks have passed since their well visit. Participants contacted by phone will have the option to complete the survey verbally over the phone and participants contacted by mail will have the option to complete a mailed version of the survey. For Part 1, parents will not be compensated for participating. For Part 2, participants will be eligible for an incentive via lottery for survey completion (e.g., $100 gift card). Note that parents who receive the Part 2 survey via mail to complete and return to us will also be sent a consent form to review along with their completed survey. Participants will be informed that they should not complete the survey until they have read the consent form. Parents who receive the Part 1 survey via text message will be sent a link to the consent form in the introductory text message before the survey question. For people participating in Part 1 via email or patient portal message, a link to the consent form will be included within the invitation message. The Part 1 survey consent form indicates that participants should review it before completing the survey, and completion of the survey indicates their consent to participate.

- PCP surveys (Aims 1 and 2). PCPs will be asked to complete surveys 2 times: once during the pre-implementation period (baseline survey) and once during the implementation period (implementation survey). They will be contacted via phone and email by the PIs and/or research specialists from their respective health systems and by research specialists on the Penn team using the Dillman Tailored Design Method. We have used this method in prior survey research with clinical staff. Surveys will be administered via Penn’s REDCap. If allowed by their respective systems, PCPs will receive $20 e-gift cards or an altruistic gift ($20 donation to a suicide prevention charity on their behalf) at each time point they complete a survey.

- PCP and Leader interviews (Aims 2). PCPs and leaders will also be asked to engage in a brief qualitative interview. We will purposively sample PCP survey respondents (equally distributed across health system and randomized arm), to obtain more detailed information from those demonstrating high and low *SAFE Firearm* fidelity. We will oversample for PCPs who chose to self-report firearm ownership on the survey. Additionally,

we will interview all leaders who agree to participate. If allowed by their respective systems, PCPs and leaders will receive $25 for interview participation. Data management will be decided between HFHS/KPCO/Penn once a management plan has been finalized.

- Equity Supplement. A trained research coordinator or team member will administer the one-time qualitative interview to participants verbally over the phone, or through videoconferencing software if the participant prefers. The interview will be audio-recorded. Demographics and audio recordings will be entered into and stored on REDCap, and transcribed interview data will be loaded into Nvivo (see Data Management section below for more details). Based on what is allowed at each health system, participants will receive a $50 electronic gift card (or if no gift card is permitted for research study incentives by the health system, we will send a personalized thank you email). We anticipate the interview will take about 25-30 minutes to complete.

The virtual stakeholder meetings will not be audio-recorded. The purpose of these meetings is to present our findings and identify opportunities to optimize facilitation prior to trial launch. Note that we will conduct these activities during a portion of existing, ongoing meetings with stakeholders. As currently conceptualized, the stakeholder meetings are not a research activity as no data will be collected for research purposes – the stakeholder feedback is meant to simply inform the design of the facilitation strategy. If the research team decides they want to collect any research data during these stakeholder meetings, we will submit a modification to the IRB outlining our research procedures and any applicable documents prior to implementing.

3. Data Management:
A Data Use Agreement will be established between University of Pennsylvania and the two health systems in partnership with Penn's Office of Research Services and the respective offices at the two health systems.

At the University of Pennsylvania, we will use secure, encrypted servers to host the data and conduct the analysis to minimize the risk of breach of data and confidentiality. The Penn Medicine Academic Computing Services (PMACS) will be the hub for the hardware and database infrastructure that will support the project. The PMACS provides a secure computing environment for a large volume of highly sensitive data, including clinical, genetic, socioeconomic, and financial information. PMACS requires all users of data or applications on PMACS servers to complete a PMACS-hosted cybersecurity awareness course annually, which stresses federal data security policies under data use agreements with the university. The curriculum includes Health Insurance Portability and Accountability Act (HIPAA) training and covers secure data transfer, passwords, computer security habits and knowledge of what constitutes misuse or inappropriate use of the server. We will implement multiple, redundant protective measures to guarantee the privacy and security of the participant data. All investigators and research staff with direct access to the identifiable data will be required to undergo annual responsible conduct of research, cybersecurity, and HIPAA certification in accordance with University of Pennsylvania regulations. Data will be stored, managed, and analyzed on a secure, encrypted server behind the University of Pennsylvania Health System (UPHS) firewall. All study personnel that will use this data are listed on the Penn IRB application and have completed training in HIPAA standards and the Collaborative IRB Training Initiative (CITI) human subjects research. Data access will be password protected. Whenever possible, data will be de-identified for analysis.

Equity Supplement Data Management:
All data management will be overseen by Dr. Beidas. Quantitative data management for the pilot data is consistent with procedures documented here in the protocol. Demographic data from the qualitative interviews will be entered into Research Electronic Data Capture (REDCap), a HIPAA-compliant web-based survey platform which is supported by the Penn Medicine Academic Computing Services (PMACS). REDCap provides secure, web-based applications that are flexible enough to be used for a variety of types of research, provide an intuitive interface for users to enter data and have real time validation rules (with automated data type and range checks) at the time of entry. These systems offer easy data manipulation with audit trails for reporting, monitoring and querying patient records, as well as an automated export mechanism to common statistical packages (SPSS, SAS, Stata, R/S-Plus). REDCap servers are housed in a local data center at the University of Pennsylvania and all web-based information transmission is encrypted. REDCap was developed specifically around HIPAA Security guidelines.

Audio recordings from the interviews will be digitally recorded and stored in REDCap. Audio recordings will be sent to TranscribeMe, a professional transcription service. TranscribeMe services include multiple safeguards

designed to protect the privacy and security of personal health information, along with utilizing workers specifically cleared to work with this type of sensitive information. TranscribeMe maintains crowd worker teams that are vetted, trained, and authorized to work on content containing PHI/PII. Data submitted to TranscribeMe is stored on servers located inside secure, dedicated Microsoft Azure data centers, with state-of-the-art physical and online intrusion prevention measures in place. Data is submitted and maintained through a secure file transfer protocol (SFTP) platform that has been set up specifically for HIPAA compliance. The service limits the amount of internal staff that has access to customer data within this SFTP only to essential personnel. Transcripts will be loaded into Nvivo qualitative data analysis software for management and analysis. Qualitative analysis is described in the Statistical Analysis section below.

A number of procedures will be utilized to ensure confidentiality of participant data. First, all qualitative interview participants will be assigned a random ID number. This ID number will be used on all data collected from participants. The names that correlate to those ID numbers will be kept separate, i.e. identifiable data will be stored in one file and de-identified research data will be kept in a separate file. Given COVID-19 and the geographic spread of our participants (e.g., Colorado, Michigan), this study is designed to be deployed remotely and virtually. The key linking ID numbers to participant names will only be kept in REDCap. Only the research team outlined in this application will have access to the participant's identifiable data. Electronic records (e.g., digital audio files) will be stored in REDCap as well as on a PMACS HIPAA-compliant server. All requests to use the data will be reviewed by Dr. Beidas. Any data files provided to other individuals will be de-identified and contain only the random ID numbers. Participants will be notified of the above procedures during informed consent.

4. Management of Information for Multi-Site Research where a Penn Investigator is the Lead Investigator of a Multi-Site Study:

Dr. Rinad Beidas (Penn) is the lead investigator of this multi-site study. All data will be collected from the two health systems, securely transferred to Penn, where it will be managed and analyzed. All reporting will be in line with the Penn IRB's reporting requirement of unanticipated problems involving risks to participants or others. The two health system have requested and received from their IRBs approval to cede to the Penn IRB which serves as the Single IRB for this project, and an IRB Authorization Agreement will be established once this protocol is reviewed and approved by the Penn IRB. We have outlined below each institution involved in this project and their respective responsibilities:

- *University of Pennsylvania (Penn)*. The project team at Penn will provide scientific and administrative oversight of the proposed project and all project-related activities coordinated by the investigative teams at each site. They will lead the training in facilitation at both sites and provide ongoing support to facilitators at each site. They will also lead the qualitative data collection, management and analysis. The Penn site will oversee the data analysis and interpretation of findings.
- *Henry Ford Health System (HFHS)*. The project team at HFHS will oversee the following activities at the participating clinics within their health system: the dissemination of *SAFE Firearm* program materials to clinics, brief clinician training, the build of the Epic SmartLists, and facilitation provided to clinics randomized to the Nudge+ condition. They will also coordinate the collection of all quantitative data from parent and clinician participants and ensure safe data storage using REDCap.
- *Kaiser Permanente Colorado (KPCO)*. The project team at KPCO will oversee the following activities at the participating clinics within their health system: the dissemination of *SAFE Firearm* program materials to clinics, brief clinician training, the build of the Epic SmartLists, and facilitation provided to clinics randomized to the Nudge+ condition. They will also coordinate the collection of all quantitative data from parent participants and ensure safe data storage using KPCO's REDCap. They will coordinate with Penn for quantitative data collection (surveys) from PCPs which will be stored in Penn's REDCap and qualitative (interview) data collection from PCPs and health system leaders.

5. Subject Follow-up:

If it is decided that we will follow-up with participants, we will submit a modification and outline our reconsenting plan as necessary.

STUDY PROCEDURES:

1. Detailed Description:

Program and Implementation Strategies. Leadership within both health systems has agreed to deploy implementation strategies at their respective health systems, including the *SAFE Firearm* program, *Nudge* implementation strategy (EHR SmartList), and *Nudge+* implementation strategy (EHR SmartList plus facilitation) and thus our assessment is that they are ***not human subject research activities***. As a reminder, half of the clinics will be randomized to receive the *Nudge* implementation strategy and the other half will receive the *Nudge+* implementation strategy. We have provided a brief description of the program and two implementation strategies below for context.

- *SAFE Firearm program*. *SAFE Firearm* is an adapted version of *Safety Check*, which included: (a) screening for firearms; (b) brief counseling based on a motivational interviewing approach; and (c) offering cable locks. Our adapted program maintains the mechanisms of the original *Safety Check* program (i.e., counseling and offering a cable lock) but incorporates stakeholder feedback and principles from behavioral economics to further enhance the program. *SAFE Firearm* will include: (a) brief PCP counseling provided to all parents (without screening or documentation of ownership in the EHR) using motivational interviewing about safe firearm storage; and b) offering cable locks to all parents.
- *Nudge*. Clinics randomized to the *Nudge* implementation strategy will receive the EHR SmartList only. During the study's preparation phase, the project teams at HFHS and KPCO will work with their respective pediatric clinic leadership and Epic information technology specialists to refine the design and functioning of our EHR SmartList, and to prototype and pilot it to ensure it is consistent with current workflow to be as unobtrusive as possible, consistent with typical practices in the respective health systems. SmartLists are pre-defined lists of choices that users can select using their mouse or keyboard and are particularly helpful for documenting values that a PCP is required to use repeatedly, thus saving time and keyboard strokes. We will add a SmartList to the standard Well Child Visit workflow to serve as a reminder and allow for tracking of *SAFE Firearm* implementation. For example, the PCP may be required to select a value from a drop-down list to indicate whether they provided the SAFE Firearm program to the family or offered a cable lock. This Smart List will remain turned "on" during the implementation period through the sustainment period. Clinics will be responsible for making their own decisions about storage and distribution of firearm locks.
- *Nudge+*. Clinics randomized to the *Nudge+* implementation strategy will receive the *Nudge* implementation strategy (as described above) as well as facilitation. Facilitation is external support delivered by health system employees not employed within the clinic site. It will be offered for 12 months to each clinic, in keeping with other implementation trials. We will use a train-the-trainer model to train facilitators at HFHS and KPCO to ensure they achieve facilitator core competencies with an eye toward implementation of *SAFE Firearm*. This may also involve sending key facilitators to a training around how to successfully implement facilitation. The role of the facilitator is to engage with study clinics, to assist each clinic in setting change and performance goals around the implementation of *SAFE Firearm*, and to troubleshoot implementation barriers. Our approach to facilitation was informed by established facilitation manuals (i.e., Veteran Health Affairs QUERI facilitation manual and AHRQ practice facilitation manual) and includes six stages. First, facilitators will engage in a pre-implementation readiness assessment for each clinic to identify potential implementation barriers and to develop relationships with stakeholders. Second, facilitators will hold kick-off meetings that signal the formal launch of the implementation strategy activities that include identifying where in the workflow *SAFE Firearm* can be implemented. This includes when *SAFE Firearm* will be delivered during the well-child visit, who in the clinic will be responsible for storing the cable locks, where the locks will be stored, and other workflow-related matters. Third, in the first 3 months of the active implementation period, facilitators will work with clinics around goal setting and metrics related to *SAFE Firearm*. During this period, the facilitator will conduct onsite/virtual visits to engage with practice leadership and PCPs once a month. In this phase, facilitators will also start to develop a sustainment plan in collaboration with stakeholders. Fourth, in months 3-9, the facilitators will engage in active implementation efforts based upon barriers assessed in the pre-implementation readiness phase as well as barriers that emerge as PCPs and clinics begin implementing. This includes established implementation strategies such as Plan-Do-Study-Act cycles and audit and feedback. All activities will be tracked via established logs to ensure the ability to understand which strategies are delivered and for implementation fidelity purposes. Fifth, in months 9-12, facilitators will engage in continued efforts to maintain gains and begin to enact the sustainment plan developed in the kick-off meeting. Sixth, in month 12, facilitation activities will complete and the clinics will transition to the formal maintenance period. Throughout the course of the active implementation period, facilitators will offer expert consultation (i.e., 2 webinars and technical assistance via email and phone as needed); and regular peer-to-peer calls supported by facilitators where clinics can share their experience.

**Timeline**	**Year 1**				**Year 2**				**Year 3**				**Year 4**				**Year 5**			
**Quarter**	**1**	**2**	**3**	**4**	**1**	**2**	**3**	**4**	**1**	**2**	**3**	**4**	**1**	**2**	**3**	**4**	**1**	**2**	**3**	**4**
Study planning	●	●	●	●																
PCP surveys (Baseline)					●	●														
PCP surveys (Implementation)									●											
Facilitation							●	●	●	●										
EHR Nudge							●	●	●	●	●	●	●	●						
Parent surveys (Aims 1 and 3)					●	●	●	●	●	●	●	●	●	●						
Lock Distribution & Cost Data (Aim 1)					●	●	●	●	●	●	●	●	●	●						
EHR/Admin Data collected (Aims 1 & 3)					●	●	●	●	●	●	●	●	●	●						
PCP and Leader interviews (Aim 2)											●	●	●	●						
Blue refers to the pre-implementation period; green refers to the active implementation period; gray refers to the sustainment period																				

The above Timeline outlines the procedures and project periods. As described in the **Target Population and Accrual** section above, participants will include parents, PCPs, and leaders within 41 pediatric clinics across two health systems (HFHS and KPCO). Parents will be asked to complete surveys 1-week following their completed well child visit to collect fidelity and acceptability (both Aim 1) and firearm storage behavior (Aim 3). PCPs will be asked to complete surveys at two time points (Aims 1 and 2): pre-implementation (baseline survey) and during active implementation (implementation survey). Logs will be collected from clinic staff to collect clinic-level data related to cable lock distribution and cost (Aim 1) and EHR data will be collected to measure reach (Aim 1) and youth suicide attempts, deaths, and unintentional firearm injury and mortality (Aim 3). We will conduct qualitative interviews during the sustainment period with a subset of PCPs and leaders (Aim 2).

<u>Equity Supplement Procedures</u>: The Penn interview team will work with the ASPIRE Site PIs at Henry Ford Heath System and Kaiser Permanente Colorado to identify potential participants who engaged in piloting procedures. The site PIs and/or Penn team will reach out via email with an invitation to participate. If clinicians are interested in participating, the interview team will schedule a date and time that is convenient to conduct a qualitative interview by phone or videoconferencing if the participant prefers. During the call but before the interview begins, the interview team will obtain consent (see Informed Consent section below for more details). The interview will not take place if the participant does not provide informed consent. The interview will be audio-recorded. Based on what is allowed at each health system, participants will receive a $50 electronic gift card (or if no gift card is permitted for research study incentives by the health system, we will send a personalized thank you email). After analyzing the interviews, we will use existing, ongoing meetings with clinical stakeholders to gather feedback on our findings. We will present our findings, which will allow stakeholders to select specific strategies (e.g., collaborative problem-solving) within facilitation to target disparities in SAFE Firearm delivery. We expect that this step will prime facilitation for trial launch and increase the likelihood of equitable outcomes.

2. Data Collection:

- <u>Quantitative Survey Data and Qualitative Interview Data</u>. The research teams at HFHS and KPCO will work with their respective health systems to obtain necessary contact information to complete data collection. KPCO and HFHS will outreach their respective health system parents for quantitative (survey) data collection by phone, email, text message, electronic patient portal (e.g., My Chart), and/or mail to recruit them to participate in quantitative surveys with outreach frequency consistent with their health system policy. The research team at Penn will work with the research teams at HFHS and KPCO to contact PCPs and Leaders at both health systems by phone or email to invite them to participate in quantitative data collection (surveys) and qualitative interviews. During all recruitment activities with all potential participants, the research teams will make clear that participation in the research project is 100% voluntary and not mandatory. We will also ensure that leaders are aware that they cannot mandate that employees of their health system or that patients participate and that any involvement (or decision not to participate) in the proposed work will not have an adverse impact on their employment or care. All language used during the consenting process will thoroughly describe the procedures to be followed in the study. All language used during the consent process, surveys, and interview guides will be

submitted to the oversight IRB prior to use. Surveys will collect information on clinician demographics and clinician specialty. See **Administration of Surveys and/or Process** section above for additional details.

- Cost Data. Cost will be measured using a pragmatic method to capture all resources needed to deploy *Nudge* and *Nudge+* so that decision makers can have the information they need to take this approach to scale within their respective systems. Cost data will be collected from all clinics who are receiving the *SAFE Firearm* program (which, as a reminder, is not a human subject research activity and will be implemented across all clinics) using Excel templates collected from clinics on a monthly basis. These cost data will not include any identifiable information about any research participants and thus we believe the collection of this cost data is ***not human subject research***.

- EHR/Administrative Data (which includes data from the EHR, VDW, and Administrative Data). By virtue of being a part of the Mental Health Research Network (MHRN), both health systems deposit EHR and claims data into a virtual data warehouse (VDW). The VDW overcomes privacy concerns using a distributed data-model with standard variable definitions across systems, enabling research on large datasets without the need to share identifiable health information. We will collect the number of parent-youth dyads who receive the program from the VDW (i.e., reach). As part of this project, we will also utilize child medical records, claims, and administrative data extracted from the local VDW and EHR, as well as other existing health records (e.g., State Mortality records) and administrative databases. Although we expect our groups to be balanced with respect to patient and clinician characteristics, we may also extract the below data elements as relevant covariates at multiple time points over the course of the project. If we would like to collect data beyond the five years of this grant, we will communicate our plan to the IRB prior to collection.
  - Healthcare Utilization: Inpatient, ER, and outpatient visits, procedures (identified using CPT codes) and diagnoses (identified using ICD codes) including but not limited to psychiatric diagnoses, diagnoses indicating probable or possible suicide attempt, injury or poisoning, and other medical diagnoses. Dates of utilization, procedure and diagnoses will also be collected. Any 42 CFR Part 2 covered data will be collected and used in compliance with those requirements
  - Pharmacy Data: Ordered and filled prescriptions, including dates
  - Patient Reported Outcomes Data: Patient reported outcome measures recorded in the EHR (e.g., PHQ, GAD-7)
  - Enrollment: types of insurance coverage, dates of enrollment and unenrollment
  - Demographics: age, sex, race/ethnicity, census block variables (e.g., income, educational attainment), patient language
  - State or National Mortality Data: date and cause of death
  - Well-visit firearm safe storage and cable lock documentation

3. Genetic Testing:
n/a

4. Use of Deception:
n/a

5. Statistical Analysis:

- Aim 1 Analysis. Our power analyses are based upon the primary implementation outcome of fidelity, consistent with best practices for hybrid type III trials.[61] All other implementation outcomes are secondary. Our sample sizes for the project are determined by the fixed number of parent-youth dyads, PCPs, and pediatric clinics within our two health systems. Data are clustered within health system, clinic, and PCP. We expect that a maximum of 38,989 parents of youth will be eligible to participate each year, and expect to include data from 151 PCPs, within 41 clinics. Based on a 31.58% response rate to parent surveys, we anticipate obtaining parent-reported PCP fidelity ratings from 23,394 parents during the one year active implementation period. We base our power calculations on our study design and prior research from our group demonstrating that current PCP use of the *SAFE Firearm* program components are 28% for brief counseling on safe firearm storage and 2% for offering cable locks.[14] We used the PASS power calculation program,[62] assuming an estimate of an ICC of .07 for explained variance among both PCPs and clinics,[63] and conclude that we will have 80% power to detect small effects of the less intensive implementation condition (*Nudge*) on fidelity (Cohen's d = 0.25 and 0.29, respectively).[64] Alpha is set at .05 (two-tailed). A small to moderate effect is reasonable to expect based on prior

research demonstrating that EHR nudges using active choice approaches produce effects in this range.[51] Because the clinics will be our unit of randomization, it was not necessary to account for the health system ICC in the power analysis. We will account for any differences between health systems by weighting health system the strongest in our covariate constrained randomization. Also, we will conduct a sensitivity analysis that controls for health system as a fixed effect in our analyses and will explore whether intervention effectiveness varies significantly by health system. Our approach is consistent with similar work that has been published in leading medical journals (i.e., Navathe et al., 2019, *JAMA*, *Sharma et al., 2019, JAMA Oncology*). In Aim 1, the primary dependent variable is fidelity. For each observation period (pre-implementation, active implementation, sustainment) and for each implementation condition (*Nudge, Nudge+*), we will describe the proportion of parents who reported having received the program with fidelity. We will calculate fidelity using three binary outcomes: received counseling (yes/no), offered lock (yes/no), both (yes/no). We will then conduct separate models. In the first set of models, we will examine whether the odds of receiving components of *SAFE Firearm* increased more between the pre-implementation and active implementation periods in the *Nudge+* group compared with the *Nudge* group. In the second set of models, we will repeat the analysis using the active implementation and sustainment periods. The fixed effects in the model will include implementation condition (*Nudge, Nudge+*), time (pre-implementation, active implementation, sustainment), and their interaction; the magnitude and statistical significance of the parameter estimate for the interaction term will be of primary interest. Given the nested data (i.e., parent-youth dyads nested within PCPs; PCPs nested within clinics; clinics nested within health systems), analyses will rely on 3-level logistic mixed effects regression models for fidelity as a dichotomous implementation outcome.[65,66] Linear mixed effects regression will be used to model continuous secondary outcomes (e.g., acceptability). For all models, our independent variable of interest will be a binary indicator of implementation condition assigned at the clinic level. All analyses will be conducted using MPlus to appropriately estimate multilevel regression models.[67] Although we expect our groups to be balanced with respect to patient and PCP characteristics, we will consider including the following variables, extracted from the VDW, as relevant covariates: health system (HFHS, KPCO); youth demographics (age, gender, race and ethnicity, patient language); PCP demographics (specialty/degree, gender, year graduated from medical school); and public census data (socioeconomic indicators for patients based on geocoded patient addresses). In sensitivity analyses, we will examine each set of models separately by health system to account for structural differences in how care is delivered within the two systems. Sex and race/ethnicity, drawn from the EHR and survey, will be added to all models as fixed effects to account for the potential role they may play in implementation strategy and intervention effectiveness. We will also investigate whether sex moderates the effects of the implementation strategies. Given the potential high non-response to the parent survey following the visit, we will use inverse probability weighting to adjust for parental survey non-response (Skinner, 2011). Specifically, inverse probability weighting allows us to use patient, clinician, and clinic information from the EHR about both respondents and non-respondents to assign larger weights to survey responses from participants who are under-represented in the sample. For example, if we find that parents who are ethnic and/or racial minorities have a lower response rate than parents who are not ethnic and/or racial minorities, inverse probability weighting would give more weight in the analysis to survey responses from parents who are ethnic/racial minorities.

- <u>Aim 2 Analysis</u>. *Quantitative analysis.* For our 2-2-1 mediation analyses, we will have approximately 76 PCPs and 20 clinics per study arm. Using the PASS power calculation program,[62] we anticipate these sample sizes will yield sufficient power to detect a moderate indirect effect (Cohen's d = .44) through the clinic-level mediator (i.e., clinic adaptive reserve). We assume there will be a large effect of the strategy on the mediator and of the mediator on the outcome based on previous research.[68] This is a conservative estimate because the calculation does not account for the additional power that will be gained due to the three measurement time-points for each PCP. We will conduct exploratory analyses to investigate intrinsic and extrinsic motivation as a mediator. Mediation will be tested using the product of coefficients approach for multilevel mediation analysis,[69-71] which we have used in previous studies.[72] In this approach, the total effect of the implementation strategy is parsed into direct and indirect effects associated with the mediator. We will test each mechanism in a separate 2-2-1 mediation model. Path "a" represents the effect of the implementation strategy (*Nudge, Nudge+*) at the clinic level on the clinic-level mediator (i.e., clinic adaptive reserve). Path "b" represents the relationship between the clinic-level mediator (i.e., adaptive reserve) and the three binary fidelity outcomes (counseling, cable lock, both). We will calculate change in fidelity for each of the three time-points using the 6 month period preceding each survey administration. An unbiased estimate of the mediated effect is derived via the product of the "a" and "b" paths (i.e., a*b) from these analyses.[70,71,73] We will test the statistical significance of the

mediated effects using the joint significance test.[74] We will use Monte Carlo simulation methods to derive confidence intervals.[74-77] We will repeat this model for our exploratory mediator, motivation. Moderators of the strategies' effects will be tested separately by adding terms for each moderator and its interaction with the implementation strategy to the Aim 1 models only during the active implementation period. These models will estimate the conditional relationships between the implementation strategies and implementation outcomes across different values of the putative moderators. *Qualitative analysis and mixed methods*. Text answers from open-ended questions with parents from Aim 1, and digitally recorded and transcribed interviews with PCPs and leaders on the mechanisms of the implementation strategies, will be loaded into NVivo qualitative data analysis software. Analysis will be led by the research team guided by an integrated approach,[78] which outlines a rigorous, systematic method for analyzing qualitative data using an inductive and deductive process of iterative coding to identify recurrent themes, categories, and relationships. We will ensure at least 80% interrater reliability amongst our coders (we will have at least two). The structure of our mixed methods approach is sequential (quantitative data is collected before qualitative data and quantitative data is weighed more strongly than qualitative; QUAN>qual). The function is of complementarity (to elaborate upon the quantitative findings to understand the *how* of implementation), and the process is connecting (having the qualitative data set build upon the quantitative data set).[79] To integrate the quantitative and qualitative results, we will follow guidelines for best practices in mixed methods[80] as well as our previous experiences with mixed methods implementation research.[81-84]

- <u>Aim 3 Analysis.</u> We do not include a power analysis because in a hybrid type III effectiveness-implementation trial, power is based on implementation outcomes.[61] We note that we will not be powered to detect reductions in youth suicide attempts, deaths, and unintentional firearm injury and mortality given very low prevalence. However, we will conduct exploratory analyses using the same modeling techniques described in Aim 1 to assess the impact of the implementation conditions on parent-reported firearm storage behavior, youth suicide attempts, deaths, and unintentional firearm injury.

- <u>Equity supplement Analysis</u>: For analysis of the pilot data, we will fit two separate inverse probability weighted GEE logistic regression models to analyze the data. We will use a parameter of 10% difference in program delivery for youth in either group of interest to explore further. For analysis of the clinician interviews, we will use rapid qualitative analytic techniques, specifically structured summaries and matrix displays. We will begin analysis by systematically condensing the data (i.e., transcripts, field notes) to create an inventory of the most contextually meaningful data segments and quotations for each data collection episode. We will develop a structured template with key domains (i.e., clinical encounter, patient factors) to standardize the capture of interview content and increase the accessibility of the data. After all interviews are transformed onto structured templates, we will then transfer the summary points into a matrix, which will expedite recognition of similarities, differences, and trends across participants. We will use the matrix to develop barriers for program delivery.

RISK/BENEFIT ASSESSMENT:

1. Risks:

There are minimal risks to participants in this trial. The *SAFE Firearm* program is being implemented at both health systems. The program could increase the time PCPs spend with parents discussing firearm storage. However, shared-decision making is a high priority and evidence-based practice for pediatric primary care and increased time on this topic is likely valuable. In prior studies, nudges in the EHR were associated with minimal burden on PCPs. There is a risk of breach of data and confidentiality, however we described the precautions in place to securely manage this data in the **Data Management** section of this protocol.

Equity Supplement Risks: Same as described above. Potential harms to participants are minimal and there are no known physical, financial, or legal risks to participating in the study. However, interview participants will be asked about their perspectives of the implementation of *SAFE Firearm* in their health system. Participants may feel temporarily uncomfortable while participating in the interview. Dr. Beidas is a licensed clinical psychologist with extensive experience conducting qualitative research in community settings, and she will be available to speak with any participants who feel unduly distressed and make appropriate referrals as needed. An additional potential risk is the potential for loss of confidentiality. Participants may disclose personal sensitive information during interviews. All information shared during interviews will be kept private and confidential except in the case that disclosure is

required by law. An exception to confidentiality is if a participant were to report child abuse or neglect, or if they report significant suicidal or homicidal ideation or intent to the research team. Any information about child abuse or intent to harm self or others will be reported to authorities, as required by law. No identifiable information that participants provide will be shared with any individuals outside of the study procedures outlined in this protocol. During the consent process, potential participants will be told about the alternative to participation (i.e. to not participate), and that they can withdraw at any time without penalty and can request their data be destroyed by submitting a written request to the research team. Potential participants will also be told that their decision to participate will not affect services or employment. This information is also outlined in the consent form.

2. Benefits:

Parents, PCPs, and leaders will receive no direct benefits from participating in this study. Participants who enroll in this trial will benefit from the knowledge that they are contributing in an important way to potentially furthering scientific knowledge about reducing firearm access and related suicide risk. Both health systems included in our study indicated that they would adopt this approach if we can demonstrate its effectiveness in this trial, suggesting the sustainability of the proposed work. Furthermore, the evidence and insights generated can be taken to scale in the MHRN which includes 14 healthcare systems which are closely integrated. The knowledge gained on how to implement a program like *SAFE Firearm* could be applied to other pediatric primary care settings and implemented at other health systems.

Equity Supplement Benefits: Participants may find satisfaction in sharing their perspectives with our research team as their feedback will be used to inform our facilitation implementation strategy. Their feedback may help the research team better understand perspectives about the barriers to implementation. The ratio of risks to benefit is reasonable given the importance of the information to be gleaned by this research.

3. Subject Privacy:

Privacy will be given utmost consideration and is highly valued in the proposed research. No research activities involve any direct interaction with subjects that would pose risk to their privacy.

Equity Supplement Subject Privacy: Privacy will be given utmost consideration and is highly valued in the proposed research. Participants will complete the interview over the phone or by videoconferencing if the participant prefers, and so can elect where they take the phone call; the interview will be in a private location where no one can overhear the content.

4. Subject Confidentiality:

Confidentiality refers to the subject’s understanding of, and agreement to, the ways in which identifiable information will be stored and shared.

How will confidentiality of data be maintained? Check all that apply.

☒ Paper-based records will be kept in a secure location and only be accessible to personnel involved in the study.

☒ Computer-based files will only be made available to personnel involved in the study through the use of access privileges and passwords.

☒ Prior to access to any study-related information, personnel will be required to sign statements agreeing to protect the security and confidentiality of identifiable information.

☒ Whenever feasible, identifiers will be removed from study-related information.

☒ A Certificate of Confidentiality will be obtained, because the research could place the subject at risk of criminal or civil liability or cause damage to the subject’s financial standing, employability, or liability.

☒ A waiver of documentation of consent is being requested, because the only link between the subject and the study would be the consent document and the primary risk is a breach of confidentiality. (This is not an option for FDA-regulated research.)

☒ Precautions are in place to ensure the data is secure by using passwords and encryption, because the research involves web-based surveys.

☒ Audio and/or video recordings will be transcribed and then destroyed to eliminate audible identification of subjects.

☐ Other (specify):

To protect participant confidentiality, only the research team outlined in this application will have access to review identified research records. Confidentiality will be protected to the fullest extent allowable under the law. See the **Data Management** section for more details.

For data that needs to be transmitted, it will be done through a Penn-approved secure encrypted file transfer solution as is described in Penn IRB's Guidance on Electronic Data Protection Requirements for Research Involving the Use of PHI. Records will not be released without the participant's consent unless required by law (e.g., imminent risk of harm to self suspected) or court order. When results of the research are presented at scientific meetings or published, no identifying information will be included.

All identifiable data, including the master list linking identifiers to the ID number and recordings, will be destroyed in 2030, five years after the award period ends.

5. Protected Health Information

Full details regarding data sharing and data management regarding PHI and PII is outlined in the data sharing and management appendix.

6. Compensation:

- Parent surveys (Aims 1 and 3). Parent participants for Part 1 of the parent survey will not receive compensation. Parent participants for Part 2 will be eligible for an incentive (i.e., $100 e-gift card) via lottery for survey completion.
- PCP surveys (Aims 1 and 2). If allowed by their respective system, PCP participants will receive $20 e-gift cards or we will make an altruistic gift ($20 donation to a suicide prevention charity on their behalf) each time they complete a survey.
- PCP and leader interviews (Aim 2). If allowed by their respective system, PCP and leader participants will receive a $25 e-gift card for participating in an interview.
    - Equity supplement: Based on what is allowed at each health system, participants will receive a $50 electronic gift card (or if no gift card is permitted for research study incentives by the health system, we will send a personalized thank you email)..

7. Data and Safety Monitoring:

Diligent data and safety monitoring will be conducted by the PI and research team at Penn, HFHS, and KPCO throughout the conduct of this study. This monitoring plan includes tracking participant safety and demographics, monitoring the safety of data, and monitoring and appropriately reporting adverse event activity. The PI and appropriate co-investigators will review data collected to ensure that no study findings warrant immediate intervention. We believe this research poses no greater than *minimal risk* and have proposed a monitoring plan that reflects this risk level.

The PI will be responsible for oversight of potential adverse events. An adverse event (AE) is any untoward medical occurrence in a subject during participation in the clinical study. We anticipate two potential types of AEs that could be directly related to study participation. These include distress experienced with regard to research participation and breach of confidentiality and privacy. With regard to the former, we have protocols in place that include conferring with the PI (who is a licensed clinical psychologist) or a staff psychologist. With regard to the latter, we have appropriate safeguards to reduce risk of breach of confidentiality and privacy. As we are not legally mandated by any child access prevention law to disclose or report firearm storage practices, we do not anticipate AEs related to participants sharing information about their firearm storage practices with our research team. Additionally, we will defer to the respective health systems' procedures and policies related to any suicidal thoughts and/or behaviors identified during the course of this project. We appreciate the sensitive topic of discussion, and are committed to taking the appropriate steps if any unanticipated problems are identified during the course of this project. Otherwise, any risks related to additional potential AEs are not expected because the program, implementation strategies, and assessments pose minimal risk to subjects.

All members of the research team who will be involved in the design and conduct of the study must receive education in human research subjects protection from a training program that is approved by a properly constituted independent Ethics Committee or Institutional Review Board. The PI will be responsible for ensuring project faculty

and staff have the equipment and training required to protect privacy and confidentiality and will monitor and document that these individuals are properly certified. If new senior/key personnel and staff become involved in the research, documentation that they have received the required education will be included in the annual progress reports.

The Penn IRB will serve as the IRB of record for any external ethics review boards or IRBs applicable to researchers from other institutions who may have access to human research subjects identified data (i.e. HFHS, KPCO).

DSMB. Additionally, we have convened a three-member DSMB from faculty outside of the three institutions involved in this project (Penn, HFHS, KPCO). DSMB composition includes the following: (1) a health services researcher with expertise in health systems and collection of data through EHR, (2) a biostatistician with expertise in longitudinal analysis, field trials, and clinical trial data, (3) an implementation research expert and firearm researcher. The DSMB will be an independent group of experts charged with reviewing study data for data quality and integrity, adherence to the protocol, participant safety, and study conduct and progress. They will also make determinations regarding study continuations, modifications, and suspensions/terminations. DSMB members will be independent from any professional or financial conflict of interest with the research project and/or study investigators. The DSMB will meet at least once yearly via phone conference calls for the duration of the project. The DSMB will elect a Chair to moderate the meetings. At the initial meeting, the DSMB will review and approve all study protocols before study initiation to ensure participant safety. Protocols will include formal procedures for reporting and tracking all adverse reactions to the NIH and IRBs; tracking progress in the study; and identifying any need for premature termination of the protocol. At subsequent meetings, the DSMB will be provided with summary study progress reports and adverse events. The DSMB will provide a summary report following each meeting. We will not require the DSMB to conduct interim analyses of data prior to the end of the study.

8. Investigator’s Risk/Benefit Assessment:
This study presents minimal risk that is balanced by the potential benefits of the research to society. This assessment is consistent for the equity supplement.

INFORMED CONSENT:

1. Consent Process:
We are requesting a waiver of written documentation of consent (see below) from the Penn IRB for participants who agree to participate in the surveys (parents, PCPs) and/or interviews (PCPs, leaders). Because surveys will be completed via REDCap or phone and interviews will be conducted remotely, we will not have an opportunity to collect a signed copy of the consent form. We will not collect signed consent forms from participants who complete the parent survey via mail; these participants will receive a consent form to review before completing the survey. In the consent form and the introductory letter that they receive in the mail with the survey, they will be instructed that their completion and returning of the survey will be considered their consent to participate in the research study.

For the REDCap, mailed, and phone surveys, the required elements of informed consent will be described to the participant and, if they agree to participate, participants will consent by agreeing and proceeding to the electronic survey, completing the survey and returning it to the research team by mail, or providing verbal consent for surveys completed over the phone. Only participants who agree will be able to proceed to the online/verbal survey. Participants who complete the mailed survey will be explicitly instructed in both the mailed letter that accompanies the survey and the consent form that their completion of the survey is considered their consent. Additionally, the letter specifies that they should not complete the survey before reviewing the consent form and deciding to participate. For participants who complete the survey over the phone, a member of our research team will conduct informed consent remotely. We will review the required elements of informed consent and answer any questions the potential participant might have. Potential participants will be encouraged to ask questions about the project. Participants will be provided with a copy of the IRB approved consent document for their records. If they agree to participate, the research team will document the consent process in REDCap.

For the interviews, a member of our research team will conduct informed consent remotely. We will review the required elements of informed consent and answer any questions the potential participant might have. Potential participants will be encouraged to ask questions about the project. Participants will be provided with a copy of the

IRB approved consent document for their records. If they agree to participate, the interview team will document the consent process in REDCap.
All potential participants will be informed that participation is voluntary and provided with the information that they need to make an informed choice. We will provide contact information to all potential survey respondents in case they have any questions before, during, or after completing the survey. Potential interview participants will be asked if they have any questions and if they agree to participate before any research questions are asked. All participants will be informed that that their decision to participate or not will not impact their relationship (i.e., services or employment) with their health system.

For the equity supplement, we are also requesting a ***waiver of written documentation of consent*** from the IRB for participants who agree to participate in the qualitative interview. Due to the Covid-19 pandemic and the geographic spread of participants (e.g., Colorado, Michigan), we have decided to conduct these interviews by phone or videoconference to minimize risk posed to participants and to our research team. Because these meetings will be conducted by phone or videoconference, we will not have an opportunity to collect a signed copy of the consent form. A member of our interview team will conduct informed consent for qualitative interview participants over the phone or videoconference. We will review the required elements of informed consent and answer any questions the potential participant might have. Potential participants will be encouraged to ask questions about the project. Participants will be offered a copy of the IRB approved consent form for their records. If they agree to participate, the interview team will document the consent process in REDCap. All potential qualitative interview participants will be asked to confirm that they understand the information provided, understand that participation is voluntary, and feel able to make an informed choice. All participants will be assured during the informed consent process that their decision to participate or not will not impact their relationship (i.e., services or employment) with their health system.

2. Waiver of Informed Consent:

- Waiver of Informed Consent. The trial is pragmatic in nature, assessing the impact of implementation strategies (i.e. *Nudge* vs. *Nudge+*) delivered to PCPs through minor adjustments to existing workflow delivered through the EHR. Changes to workflow are by necessity systematically applied to all PCPs within the practices. Further, these changes to workflow are being implemented as part of leadership commitment to deploying the SAFE Firearm program and EHR Nudges and Facilitation with data collection to allow for evaluation (i.e., *SAFE Firearm* will be considered standard of practice). Because PCPs will still be free to engage with parents as they see fit and the implementation strategies represent minimal risk to PCPs and parents, we have requested **a waiver of informed consent** for allowing EHR/Administrative data to be collected for Aims 1 and 3. This approach will also aid implementation integrity and generalizability by avoiding behavior changes related to observation (i.e., the Hawthorne effect). The PI has worked closely with the Nudge Unit in the past on similar pragmatic trials that have received this waiver of informed consent, and our study team has designed the study with their trials in mind. As such, we believe there are several reasons to justify why a waiver of informed consent is requested (and has been granted for similar studies at Penn). First, it is not feasible to consent every provider and parent. Second, if consent was obtained from providers and parents, they would know they were being studied and this could change their behavior. This could potentially disrupt the design of the study and make interpretation of the findings challenging. Third, clinicians are not forced to implement the *SAFE Firearm* program (which, as a reminder, is an evidence-based practice that is being implemented in both health systems) and parents are not forced to engage in a conversation with their provider and they are not forced to take a cable lock. In all aims, providers can choose to use their best clinical judgement when it comes to the *SAFE Firearm* program. This research involves no more than minimal risk to the participants and will not adversely impact the rights and welfare of participants. As described above, the waiver is essential to research as this research could not be practicably carried out without this waiver.

- Waiver of Documentation of Consent for Online/Mailed/Phone Surveys. All surveys completed by parents (Aims 1 and 3) and PCPs (Aims 1 and 2) will be completed via phone, mail, or electronic platforms, and thus we are requesting waiver of written documentation of consent from the IRB. The required elements of informed consent will be described to the participant and, if they agree to participate, participants will consent by agreeing and proceeding to the electronic/phone survey. Mailed survey participants will be instructed to review the consent form before completing the survey. Only participants who agree will be able to proceed to the online/phone survey. Mailed survey participants will be instructed not to complete the survey until they have reviewed the consent form and decide to participate. For phone surveys, the required elements of consent will

be described to the potential participant at the beginning of the survey, before any research questions are asked. If the participant verbally agrees to be complete the survey, the research staff who is conducting the interview will document the consent process. This research activity presents no more than minimal risk of harm to subjects and involves no procedures for which written consent is normally required outside of the research context.

- Partial Waiver of Consent for Part 1 Parent Survey. For the one-question Part 1 parent survey, we will share a link to the consent form in the introductory message sent before the survey question text message or in the survey invitation sent via email or patient portal message. All the required elements of consent will be described to the participant. In the informed consent form, they will be instructed to review the consent form before completing the survey. The consent form will also indicate that their completion of the survey indicates their consent to participate in the research. This research activity presents no more than minimal risk of harm to subjects and involves no procedures for which written consent is normally required outside of the research context.

- Waiver of Documentation of Consent for Interviews. All interviews will be completed remotely and audio-recorded. The required elements of consent will be described to the potential participant at the beginning of the interview, before any research questions are asked. If the interview participant verbally agrees to be interviewed, the research staff who is conducting the interview will document the consent process. This research activity presents no more than minimal risk of harm to subjects and involves no procedures for which written consent is normally required outside of the research context.

- For the equity supplement, we also request a ***waiver of written documentation of informed consent*** (as described above) for the qualitative interview since the research presents no more than minimal risk of harm to subjects and involves no procedures for which written consent is normally required outside of the research context. The required elements of informed consent will be described over the phone or videoconference before any study activities occur.

RESOURCES NECESSARY FOR HUMAN RESEARCH PROTECTION:
Adequate facilities are available at Penn, HFHS, and KPCO. The members of the research team are outlined in the application and include the PI, research investigators, full time research staff, and part time undergraduate research assistants. The entire team will be overseen by the PI. All personnel will complete required training before being granted access to any identifying information. This includes training on confidentiality through the CITI course. All Penn personnel will also sign confidentiality statements. These confidentiality statements will specify the procedures for reporting unintentional breaches in confidentiality to the PI. The confidentiality statements also specify that violations of participants' confidentiality, either unintentional or deliberate, may result in termination of hire. The PI will conduct training with all research personnel regarding data, limits of confidentiality, maintaining confidentiality, and proper study procedures. Personnel from HFHS and KPCO sign similar confidentiality statements and undergo similar research training within their respective systems.

Statistical Analysis Plan

Final Statistical Analysis Plan

Date of approval by study team: 03 July 2023 (Prior to unblinding and data analyses; primary outcome data analyses commenced on 25 July 2023)

ASPIRE Statistical Analysis Plan for Primary Comparison of Nudge vs Nudge+

Primary outcome: Reach, defined as the composite (multiplicative) of two binary outcomes:

1) discussion of safe firearm storage (Yes/No)
2) offered a cable lock (Yes/No)

Therefore, the primary outcome will be coded as a 1 (successful program delivery) if both components were documented as "Yes" in the EHR; otherwise, the primary outcome will take value 0. The primary outcome is measured at the patient-visit level. If the same patient has multiple visits data from each visit will be included in the analysis. If multiple children in the same family, all visits for all children will be included.

As a secondary analysis, we will analyze 1) and 2) individually. We hypothesize that the analysis of component 2 (cable lock offers) will provide results similar to the results for the composite Reach outcome since fewer clinicians will endorse the cable lock than discussion and it is rare for clinicians to offer the lock without also doing the discussion.

Missingness in the primary outcome: Due to the nature of the EHR systems at the two sites, there is a non-negligible amount of missingness in the two binary outcomes that make up the composite Reach variable. More specifically, there is a large amount of missingness in the "offered a cable lock" outcome at one site, since clinicians were able to exit the EHR smartlist after documenting the first outcome about safe storage discussion. Unfortunately, missingness in one of the two outcomes induces missingness in the composite Reach outcome. In the primary analysis, we will treat any missing value as a "No." As a result, there will be no missing values in the Reach outcome for the primary analysis.

Statistical analysis plan:

Here, we describe the statistical analysis plan for the primary analysis of Reach.

Check balance across arms:

We will first calculate standardized mean differences (SMD) of both individual-level (sex, race, etc.) and clinic-level (size, presence of stakeholder, etc.) variables. Variables with a SMD greater than 0.2 will be included as covariates in the outcome models. The SMD for a given variable will be calculated as:

$$SMD = \frac{\bar{Y}_{2.} - \bar{Y}_{1.}}{\hat{\sigma}_{Pooled}}$$

where $\bar{Y}_{2.}$ is the sample average of Nudge+ cluster means; $\bar{Y}_{2.}$ is the sample average of Nudge cluster means; and $\hat{\sigma}_{Pooled}$ is the standard deviation of all N=30 cluster means.

Primary outcome model:

Missing values in the Reach outcome will be imputed as having not delivered the S.A.F.E. Firearm program. Given the definition of Reach will be completely observed (no missingness), we plan to model the patient-level binary Reach outcome using a logistic generalized estimating equation (GEE) with an exchangeable correlation at the clinic level. This model provides an estimate of the marginal effect of Nudge+ versus Nudge, i.e., the expected difference

in the population if all clinics had received Nudge+ compared to giving all clinics Nudge only. In addition to the clinic-level indicator of randomized treatment assignment (corresponding to the parameter of interest), we will include the following set of covariates: sex (patient-level), health system (clinic-level), clinic size measured by number of well-child visits in the year prior to the trial launch (clinic-level), and clinician partner status (clinic-level). We chose to include patient-level sex based on pilot findings that the rate of program delivery differs significantly for male versus female patients and by patient race. The three clinic-level covariates will be included because they were used in the covariate constrained randomization and are therefore anticipated to be highly related to Reach.

Because of the moderately small number of clinics in the trial, we will apply the Kauermann and Carroll small cluster correction to avoid underestimation of variance (e.g., using EMPIRICAL=ROOT statement with PROC GLIMMIX in SAS). Furthermore, if effort and availability of reliable software allow, we will use a custom nested exchangeable correlation instead of an exchangeable correlation clustering at the clinic level. The nested exchangeable structure will contain two parameters: one for a common correlation within clinician and the other for the correlation between different clinicians practicing at the same clinic. If available, the nested exchangeable correlation structure be more efficient if it more closely resembles the true correlation structure in the data.

Hypothesis testing:

We have proposed to use a three-sided hypothesis test using a Type I error of 0.05 to simultaneously test whether 1) Nudge+ and Nudge are equivalently effective, and 2) Nudge+ is superior to Nudge. This hypothesis testing framework required our statistical team to elicit an equivalence margin from the study investigators. To prioritize interpretability and actionability of our main study findings, we elicited the margin on the marginal probability scale. We determined that a margin of 10 percentage points would be reasonable to declare the two interventions equivalent. In other words, if we determine with statistical confidence that the rate of Reach in Nudge+ is more than 10 percentage points higher than the rate in Nudge, then Nudge+ will be deemed superior. A three-sided testing framework allows us to test equivalence, inferiority, and superiority without adjusting the Type I error for multiple tests. To implement this framework and perform these tests on the marginal probability scale, we will construct a series of Wald tests for the risk difference along a grid of null parameter values. The risk difference and its standard error (SE) will be obtained using PROC GLIMMIX's MARGINS statement. The SE will reflect the small cluster correction to the degrees of freedom (DF) by the EMPIRICAL statement (we will check that that the degrees of freedom match). For a given null value, μ_s, we will compute the t-statistic:

$$t_s = \frac{EST - \mu_s}{SE}$$

where EST denotes the estimated risk difference. We will compare this t-statistic to the critical value of the t-distribution with appropriate DF to obtain a p-value and reject the null hypothesis that the risk difference is equal to μ_s. We will repeat this process for a fine grid of μ_s values, $s \in \{1, \dots, S\}$, and form a confidence interval for the true risk difference. If the confidence interval is completely contained within the equivalence margin, Nudge and Nudge+ will be concluded to be equivalently effective. If the lower limit of the confidence interval is greater than the upper limit of the equivalence margin, Nudge+ will be deemed superior to Nudge.

SAP amendments

1. Details were added to further operationalize the analysis plan from what was originally specified in the study's protocol paper (Beidas et al., 2021).

2. The primary outcome of the study was changed from parent-reported receipt of *S.A.F.E. Firearm* (i.e., fidelity) to clinician-reported delivery of *S.A.F.E. Firearm* in the electronic health record (i.e., reach).
   - Both the study's National Institute of Mental Health Program Officer and Data Safety and Monitoring Board approved of this change on 02 March 2022, ahead of the trial's launch on 14 March 2022.
   - A detailed description of the rationale for this change is below as submitted to funding agency, National Institute of Mental Health.

Summary. Insights from behavioral economics, or how individuals' decisions and behaviors are shaped by finite cognitive resources (e.g., time, attention) and mental heuristics, have been underused in efforts to increase the use of evidence-based practices in implementation science. Using the example of firearm safety promotion in pediatric primary care, which addresses an evidence-to-practice gap in universal suicide prevention, we aim to determine: is a less costly and more scalable behavioral economic-informed implementation strategy (i.e., "Nudge") powerful enough to change clinician behavior or is a more intensive and expensive facilitation strategy needed to overcome implementation barriers? The Adolescent and child Suicide Prevention in Routine clinical Encounters (ASPIRE) hybrid type III effectiveness-implementation trial uses a longitudinal cluster randomized design. We will test the comparative effectiveness of two implementation strategies to support clinicians' use of an evidence-based firearm safety practice, *S.A.F.E. Firearm*, in 32 pediatric practices across two health systems. All pediatric practices in the two health systems will receive *S.A.F.E. Firearm* materials, including training and cable locks. Half of the practices (k = 16) will be randomized to receive Nudge; the other half (k = 16) will be randomized to receive Nudge plus 1 year of facilitation to target additional practice and clinician implementation barriers (Nudge+). The primary implementation outcome is parent-reported clinician fidelity to the *S.A.F.E Firearm* program. Secondary implementation outcomes include reach and cost. To understand how the implementation strategies work, the primary mechanism to be tested is practice adaptive reserve, a self-report practice-level measure that includes relationship infrastructure, facilitative leadership, sense-making, teamwork, work environment, and culture of learning. The ASPIRE trial will integrate implementation science and behavioral economic approaches to advance our understanding of methods for implementing evidence-based firearm safety promotion practices in pediatric primary care. The study answers a question at the heart of many practice change efforts: which strategies are sufficient to support change, and why? Results of the trial will offer valuable insights into how best to implement evidence-based practices that address sensitive health matters in pediatric primary care.

Description of problem. Parental response to our brief survey, the method through which we collect our primary implementation outcome of fidelity, has been lower than anticipated in the pilot and pre-implementation period. Our health system partners report that overall survey response in the health systems is much lower across all projects since COVID-19. The major methodological threats of a low response rate include: (a) concerns about representativeness; (b) lower power; and (c) the need to impute missing data for most of the sample. Given these concerns, our DSMB methodologist, Danny Almirall, PhD, recommended that we switch our primary outcome to reach as measured by physician electronic medical record documentation.

Steps taken. We began piloting the parent survey in May 2021 to maximize parental response rate. Our initial plan, as proposed in the grant submission, was to engage in a combination of email, text message, and mailed letters (i.e., methods found to be effective in previous work in these health systems). From May 2021 to February 2022, we conducted 13 pilots to maximize response rate, systematically changing how we approached collecting this data to ascertain the most effective approach (e.g., changing the order of outreach methods; adding messaging through patient portals, adding phone calls). Using a range of approaches, we obtained a 23% response rate, with a strong signal that phone calls are the most effective manner to obtain parental responses.

Given our desire to keep fidelity as our primary outcome, we tried two additional approaches to increase response rate. First, we piloted sending a one-question survey via email and/or text to reduce the friction and time commitment associated with responding. This resulted in a 10% response rate and is likely due to common carriers blocking text messages that include ‘firearm,’ even though we tried many strategies to circumvent these blocks. Furthermore, it resulted in loss of valuable data since only 1 question could be asked.

Second, we tried an intensive outreach approach that included 2+ phone calls to a smaller subset of randomly selected patients (n = 100) per health system every 2 weeks. This approach allowed us to reach a 37% response rate, which is the highest response rate we have been able to obtain.

The intensive outreach approach is the most effective one. Unfortunately, the sites are not resourced from a staff perspective to engage in this approach. Making phone-calls to 100 parents every 2 weeks per site requires hundreds of phone calls and places substantial strain on our health system partners with their current staffing model. We have explored budget reallocations but we will not be able to reallocate sufficient additional resources to the sites to support the additional staff needed for this resource intensive approach, given initial budgetary cuts and unanticipated increases to the cost of gun locks due to supply chain issues.

Proposed change. We propose to change the primary outcome in the ASPIRE trial from fidelity, measured via parental report, to reach, measured via physician documentation in the EHR. Our health systems have built integrated templates in the electronic medical record for the physicians as part of the trial that enable seamless documentation and efficient data extraction. We will continue to collect fidelity as a secondary outcome from a subset of participants.

Advantages of this approach. First, we will have a complete dataset with no missing data, thus strengthening the rigor of our conclusions. Second, while we acknowledge reach will likely be an overestimate of what is occurring, the bias will impact the two arms in the same way (see table below). Third, we will still collect fidelity from a subset of participants. Our partners are currently calculating the number of patients per two weeks that they can outreach to using the intensive outreach approach. We will work with our biostatistician, Dr. Kristin Linn, to potentially adjust for misclassification bias in our reach estimates by using the smaller, gold-standard validation sample of fidelity data.

**Comparison of reach and fidelity (October 21-Feb 22)**		
	**EHR – Reach**	**Survey – Fidelity**
**Counseling**	56.3%	47.9%
**Locks**	46.4%	31.8%
**Both**	45.3%	30.0%

Original Statistical Analysis Plan

Date of approval by study team: 09 August 2021 (Prior to trial launch on 14 March 2022)

The original statistical analysis plan is described in the study's protocol paper:

Beidas RS, Ahmedani BK, Linn KA, et al. Study protocol for a type III hybrid effectiveness-implementation trial of strategies to implement firearm safety promotion as a universal suicide prevention strategy in pediatric primary care. Implement Sci 2021;16.

Study blinding details

The study's lead biostatistician (KAL) generated the randomization scheme and assigned clinics to study arm. The research team at each health system (Site PI JMB and study staff LQ and LW for Colorado site; Site PI BKA and study staff CP for Michigan site) enrolled clinics.

Lead biostatistician KAL generated the randomization scheme and allocated clinics (by name) to study condition. The clinics were assigned a random ID number and study arms were also assigned a random ID number by author CJ. Study arm and clinic names were not used when discussing study activities with the blinded individuals throughout the trial. The study's Principal Investigator and this manuscript's first author (RSB), the biostatistician team (KAL, SCM), and the study's data analyst (KB) were blinded to clinic assignment at the time of data analysis. The ID numbers were listed in lieu of clinic and study arm names in the datasets used for analysis. The blinded individuals were not unblinded to clinic names and study arm until after analyses had been completed and manuscript writing commenced.

References

1. Ruch DA, Sheftall AH, Schlagbaum P, Rausch J, Campo JV, Bridge JA. Trends in suicide among youth aged 10 to 19 years in the United States, 1975 to 2016. *JAMA Netw Open*. 2019;2(5):e193886-e193886. doi:10.1001/jamanetworkopen.2019.3886
2. National Center for Injury Prevention and Control. 10 leading causes of death by age group, United States – 2017. https://www.cdc.gov/injury/images/lc-charts/leading_causes_of_death_by_age_group_2017_1100w850h.jpg. Accessed September 30, 2019.
3. Kposowa A, Hamilton D, Wang K. Impact of firearm availability and gun regulation on state suicide rates. *Suicide Life Threat Behav*. 2016;46(6):678-696. doi:10.1111/sltb.12243
4. American Academy of Pediatrics. Profile of pediatric visits: annualized estimates 2000-2004. https://www.aap.org/en-us/Documents/practicet_Profile_Pediatric_Visits.pdf. Accessed January 31, 2019.
5. Ahmedani BK, Simon GE, Stewart C, et al. Health care contacts in the year before suicide death. *J Gen Intern Med*. 2014;29(6):870-877. doi:10.1007/s11606-014-2767-3
6. Barkin SL, Finch SA, Ip EH, et al. Is office-based counseling about media use, timeouts, and firearm storage effective? Results from a cluster-randomized, controlled trial. *Pediatrics*. 2008;122(1):e15-e25. doi:10.1542/peds.2007-2611
7. Carbone PS, Clemens CJ, Ball TM. Effectiveness of gun-safety counseling and a gun lock giveaway in a Hispanic community. *Arch Pediatr Adolesc Med*. 2005;159(11):1049-1054. doi:10.1001/archpedi.159.11.1049
8. Grossman DC, Cummings P, Koepsell TD, et al. Firearm safety counseling in primary care pediatrics: a randomized, controlled trial. *Pediatrics*. 2000;106(1):22-26. doi:10.1542/peds.106.1.22
9. Stevens MM, Olson AL, Gaffney CA, Tosteson TD, Mott LA, Starr P. A pediatric, practice-based, randomized trial of drinking and smoking prevention and bicycle helmet, gun, and seatbelt safety promotion. *Pediatrics*. 2002;109(3):490-497. doi:10.1542/peds.109.3.490
10. Dowd MD, Sege RD. Firearm-related injuries affecting the pediatric population. *Pediatrics*. 2012;130(5):e1416-e1423. doi:10.1542/peds.2012-2481
11. Leshner AI, Altevogt BM, Lee AF, McCoy MA, Kelley PW. Priorities for research to reduce the threat of firearm-related violence. Institute of Medicine National Research Council of the National Academies. https://www.nap.edu/catalog/18319/priorities-for-research-to-reduce-the-threat-of-firearm-related-violence. Accessed September 30, 2019.
12. Wintemute GJ, Betz ME, Ranney ML. Yes, you can: physicians, patients, and firearms. *Ann Intern Med*. 2016;165(3):205-213. doi:10.7326/m15-2905
13. Cunningham RM, Carter PM, Ranney ML, et al. Prevention of firearm injuries among children and adolescents: consensus-driven research agenda from the Firearm Safety Among Children and Teens (FACTS) consortium. *JAMA Pediatrics*. 2019;173(8):780-789. doi:10.1001/jamapediatrics.2019.1494
14. Beidas RS, Jager-Hyman S, Becker-Haimes E, et al. Acceptability and use of evidence-based practices for firearm storage in pediatric primary care. *Acad Pediatr*. 2019;19(6):670-676. doi:10.1016/j.acap.2018.11.007
15. Ngo QM, Sigel E, Moon A, et al. State of the science: a scoping review of primary prevention of firearm injuries among children and adolescents. *J Behav Med*. 2019;42(4):811-829. doi:10.1007/s10865-019-00043-2
16. Monuteaux MC, Azrael D, Miller M. Association of increased safe household firearm storage with firearm suicide and unintentional death among US youths. *JAMA Pediatrics*. 2019;173(7):657-662. doi:10.1001/jamapediatrics.2019.1078
17. Damschroder LJ, Aron DC, Keith RE, Kirsh SR, Alexander JA, Lowery JC. Fostering implementation of health services research findings into practice: a consolidated framework for advancing implementation science. *Implement Sci*. 2009;4. doi:10.1186/1748-5908-4-50
18. Jager-Hyman S, Wolk CB, Ahmedani BK, et al. Perspectives from firearm stakeholders on firearm safety promotion in pediatric primary care as a suicide prevention strategy: a qualitative study. *J Behav Med*. 2019;42(4):691-701. doi:10.1007/s10865-019-00074-9
19. Wolk CB, Van Pelt AE, Jager-Hyman S, et al. Stakeholder perspectives on implementing a firearm safety intervention in pediatric primary care as a universal suicide prevention strategy: a qualitative study. *JAMA Netw Open*. 2018;1(7):e185309-e185309. doi:10.1001/jamanetworkopen.2018.5309
20. Patel MS, Day SC, Halpern SD, et al. Generic medication prescription rates after health system-wide redesign of default options within the electronic health record. *JAMA Intern Med*. 2016;176(6):847-848. doi:10.1001/jamainternmed.2016.1691

21. Sharma S, Guttmann D, Small DS, et al. Effect of introducing a default order in the electronic medical record on unnecessary daily imaging during palliative radiotherapy for adults with cancer: a stepped-wedge cluster randomized clinical trial. *JAMA Oncology*. 2019;5(8):1220-1222. doi:10.1001/jamaoncol.2019.1432
22. Meeker D, Linder JA, Fox CR, et al. Effect of behavioral interventions on inappropriate antibiotic prescribing among primary care practices: a randomized clinical trial. *J Am Med Assoc*. 2016;315(6):562-570. doi:10.1001/jama.2016.0275
23. Patel MS, Asch DA, Volpp KG. Nudge units to improve the delivery of health care. NEJM Catalyst. https://catalyst.nejm.org/nudge-unit-improve-health-care-delivery/. Accessed September 30, 2019.
24. Baskerville NB, Liddy C, Hogg W. Systematic review and meta-analysis of practice facilitation within primary care settings. *Ann Fam Med*. 2012;10(1):63-74. doi:10.1370/afm.1312
25. Ritchie MJ, Dollar KM, Miller CJ, et al. Using implementation facilitation to improve care in the Veterans Health Administration (version 2). Veterans Health Administration Quality Enhancement Research Initiative (QUERI) for Team-Based Behavioral Health. https://www.queri.research.va.gov/tools/implementation/Facilitation-Manual.pdf. Accessed September 30, 2019.
26. Agency for Healthcare Research and Quality. The practice facilitation handbook: training modules for new facilitators and their trainers. https://www.ahrq.gov/sites/default/files/publications/files/practicefacilitationhandbook.pdf. Accessed September 30, 2019.
27. Glenn CR, Franklin JC, Nock MK. Evidence-based psychosocial treatments for self-injurious thoughts and behaviors in youth. *J Clin Child Adolesc Psychol*. 2015;44(1):1-29. doi:10.1080/15374416.2014.945211
28. Centers for Disease Control and Prevention. Compressed mortality file 1999-2010 – CDC WONDER Online Database. https://wonder.cdc.gov/controller/datarequest/D140. Accessed September 30, 2019.
29. Spicer RS, Miller TR. Suicide acts in 8 states: incidence and case fatality rates by demographics and method. *Am J Public Health*. 2000;90(12):1885-1891. doi:10.2105/ajph.90.12.1885
30. Miller M, Azrael D, Barber C. Suicide mortality in the United States: the importance of attending to method in understanding population-level disparities in the burden of suicide. *Annu Rev Public Health*. 2012;33:393-408. doi:10.1146/annurev-publhealth-031811-124636
31. Azrael D, Cohen J, Salhi C, Miller M. Firearm storage in gun-owning households with children: results of a 2015 national survey. *J Urban Health*. 2018;95(3):295-304. doi:10.1007/s11524-018-0261-7
32. Anglemyer A, Horvath T, Rutherford G. The accessibility of firearms and risk for suicide and homicide victimization among household members: a systematic review and meta-analysis. *Ann Intern Med*. 2014;160(2):101-110. doi:10.7326/m13-1301
33. Johnson RM, Frank EM, Ciocca M, Barber CW. Training mental healthcare providers to reduce at-risk patients' access to lethal means of suicide: evaluation of the CALM project. *Arch Suicide Res*. 2011;15(3):259-264. doi:10.1080/13811118.2011.589727
34. Kruesi MJ, Grossman J, Pennington JM, Woodward PJ, Duda D, Hirsch JG. Suicide and violence prevention: parent education in the emergency department. *J Am Acad Child Adolesc Psychiatry*. 1999;38(3):250-255. doi:10.1097/00004583-199903000-00010
35. Coyne-Beasley T, Schoenbach VJ, Johnson RM. Love our kids, lock your guns: a community-based firearm safety counseling and gun lock distribution program. *Arch Pediatr Adolesc Med*. 2001;155(6):659-664. doi:10.1001/archpedi.155.6.659
36. Regier DA, Goldberg ID, Taube CA. The de facto US mental health services system: a public health perspective. *Arch Gen Psychiatry*. 1978;35(6):685-693. doi:10.1001/archpsyc.1978.01770300027002
37. Luoma JB, Martin CE, Pearson JL. Contact with mental health and primary care providers before suicide: a review of the evidence. *Am J Psychiatry*. 2002;159(6):909-916. doi:10.1176/appi.ajp.159.6.909
38. Husky MM, Olfson M, He JP, Nock MK, Swanson SA, Merikangas KR. Twelve-month suicidal symptoms and use of services among adolescents: results from the National Comorbidity Survey. *Psychiatr Serv*. 2012;63(10):989-996. doi:10.1176/appi.ps.201200058
39. Boggs JM, Simon GE, Ahmedani BK, Peterson E, Hubley S, Beck A. The association of firearm suicide with mental illness, substance use conditions, and previous suicide attempts. *Ann Intern Med*. 2017;167(4):287-288. doi:10.7326/L17-0111
40. Sheftall AH, Asti L, Horowitz LM, et al. Suicide in elementary school-aged children and early adolescents. *Pediatrics*. 2016;138(4):e20160436. doi:10.1542/peds.2016-0436
41. Klein JD, McNulty M, Flatau CN. Adolescents' access to care: teenagers' self-reported use of services and perceived access to confidential care. *Arch Pediatr Adolesc Med*. 1998;152(7):676-682. doi:10.1001/archpedi.152.7.676

42. Ahmedani BK, Westphal J, Autio K, et al. Variation in patterns of health care before suicide: a population case-control study. *Prev Med*. 2019;127. doi:10.1016/j.ypmed.2019.105796
43. Fiske ST, Taylor SE. *Social Cognition: From Brains to Culture*. 2nd ed. Sage; 2013.
44. Tversky A, Kahneman D. The framing of decisions and the psychology of choice. *Science*. 1981;211(4481):453-458. doi:10.1126/science.7455683
45. Kahneman D, Tversky A. Prospect theory: an analysis of decision under risk. *Econometrica*. 1979;47(2):263-292. doi:10.2307/1914185
46. Beidas RS, Volpp KG, Buttenheim AN, et al. Transforming mental health delivery through behavioral economics and implementation science: protocol for three exploratory projects. *JMIR Res Protoc*. 2019;8(2):e12121. doi:10.2196/12121
47. Saghai Y. Salvaging the concept of nudge. *J Med Ethics*. 2013;39(8):487-493. doi:10.1136/medethics-2012-100727
48. Doshi JA, Lim R, Li P, et al. A synchronized prescription refill program improved medication adherence. *Health Aff (Millwood)*. 2016;35(8):1504-1512. doi:10.1377/hlthaff.2015.1456
49. Patel MS, Kurtzman GW, Kannan S, et al. Effect of an automated patient dashboard using active choice and peer comparison performance feedback to physicians on statin prescribing: the prescribe cluster randomized clinical trial. *JAMA Netw Open*. 2018;1(3):e180818-e180818. doi:10.1001/jamanetworkopen.2018.0818
50. Patel MS, Volpp KG, Asch DA. Nudge units to improve the delivery of health care. *N Engl J Med*. 2018;378(3):214-216. doi:10.1056/NEJMp1712984
51. Patel MS, Volpp KG, Small DS, et al. Using active choice within the electronic health record to increase influenza vaccination rates. *J Gen Intern Med*. 2017;32(7):790-795. doi:10.1007/s11606-017-4046-6
52. Patel MS, Volpp KG. Leveraging insights from behavioral economics to increase the value of health-care service provision. *J Gen Intern Med*. 2012;27(11):1544-1547. doi:10.1007/s11606-012-2050-4
53. Hsiao CJ, Hing E. Use and characteristics of electronic health record systems among office-based physician practices: United States, 2001-2012. National Center for Health Statistics. https://www.cdc.gov/nchs/data/databriefs/db111.pdf. Accessed September 30, 2019.
54. Monegain B. More than 80 percent of docs use EHRs. *Healthcare IT News*. September 18, 2015. Accessed September 30, 2019. https://www.healthcareitnews.com/news/more-80-percent-docs-use-ehrs
55. Tabak RG, Khoong EC, Chambers DA, Brownson RC. Bridging research and practice: models for dissemination and implementation research. *Am J Prev Med*. 2012;43(3):337-350. doi:10.1016/j.amepre.2012.05.024
56. Strifler L, Cardoso R, McGowan J, et al. Scoping review identifies significant number of knowledge translation theories, models, and frameworks with limited use. *J Clin Epidemiol*. 2018;100:92-102. doi:10.1016/j.jclinepi.2018.04.008
57. Krause J, Van Lieshout J, Klomp R, et al. Identifying determinants of care for tailoring implementation in chronic diseases: an evaluation of different methods. *Implement Sci*. 2014;9. doi:10.1186/s13012-014-0102-3
58. Lewis CC, Klasnja P, Powell BJ, et al. From classification to causality: advancing understanding of mechanisms of change in implementation science. *Front Public Health*. 2018;6. doi:10.3389/fpubh.2018.00136
59. Williams NJ, Beidas RS. Annual research review: the state of implementation science in child psychology and psychiatry: a review and suggestions to advance the field. *J Child Psychol Psychiatry*. 2019;60(4):430-450. doi:10.1111/jcpp.12960
60. Kazdin AE. Mediators and mechanisms of change in psychotherapy research. *Annu Rev Clin Psychol*. 2007;3:1-27. doi:10.1146/annurev.clinpsy.3.022806.091432
61. Curran GM, Bauer M, Mittman B, Pyne JM, Stetler C. Effectiveness-implementation hybrid designs: combining elements of clinical effectiveness and implementation research to enhance public health impact. *Med Care*. 2012;50(3):217-226. doi:10.1097/MLR.0b013e3182408812
62. NCSS Statistical Software. PASS power analysis and sample size software. https://www.ncss.com/software/pass/. Accessed October 2, 2019.
63. Zyzanski SJ, Flocke SA, Dickinson LM. On the nature and analysis of clustered data. *Ann Fam Med*. 2004;2(3):199-200. doi:10.1370/afm.197
64. Cohen J. *Statistical Power Analysis for the Behavioral Sciences*. 2nd ed. Lawrence Erlbaum Associates; 1988.
65. Raudenbush SW, Bryk AS. *Hierarchical Linear Models: Applications and Data Analysis Methods*. Sage; 2002.
66. Hedeker D, Gibbons RD. *Longitudinal Data Analysis*. John Wiley & Sons; 2006.

67. Molenberghs G, Thijs H, Jansen I, et al. Analyzing incomplete longitudinal clinical trial data. *Biostatistics*. 2004;5(3):445-464. doi:10.1093/biostatistics/kxh001
68. Nutting PA, Crabtree BF, Stewart EE, et al. Effect of facilitation on practice outcomes in the National Demonstration Project model of the patient-centered medical home. *Ann Fam Med*. 2010;8(suppl 1):S33-S44. doi:10.1370/afm.1119
69. Krull JL, MacKinnon DP. Multilevel modeling of individual and group level mediated effects. *Multivariate Behav Res*. 2001;36(2):249-277. doi:10.1207/s15327906mbr3602_06
70. Pituch KA, Murphy DL, Tate RL. Three-level models for indirect effects in school- and class-randomized experiments in education. *J Exp Educ*. 2009;78(1):60-95. doi:10.1080/00220970903224685
71. Zhang Z, Zyphur MJ, Preacher KJ. Testing multilevel mediation using hierarchical linear models: problems and solutions. *Organ Res Methods*. 2009;12(4):695-719. doi:10.1177/1094428108327450
72. Williams NJ, Beidas RS. Navigating the storm: how proficient organizational culture promotes clinician retention in the shift to evidence-based practice. *PLoS One*. 2018;13(12):e0209745. doi:10.1371/journal.pone.0209745
73. Glisson C, Williams NJ, Hemmelgarn A, Proctor E, Green P. Aligning organizational priorities with ARC to improve youth mental health service outcomes. *J Consult Clin Psychol*. 2016;84(8):713-725. doi:10.1037/ccp0000107
74. MacKinnon DP, Lockwood CM, Hoffman JM, West SG, Sheets V. A comparison of methods to test mediation and other intervening variable effects. *Psychol Methods*. 2002;7(1):83-104. doi:10.1037/1082-989x.7.1.83
75. MacKinnon DP, Lockwood CM, Williams J. Confidence limits for the indirect effect: distribution of the product and resampling methods. *Multivariate Behav Res*. 2004;39(1):99-128. doi:10.1207/s15327906mbr3901_4
76. Biesanz JC, Falk CF, Savalei V. Assessing mediational models: testing and interval estimation for indirect effects. *Multivariate Behav Res*. 2010;45(4):661-701. doi:10.1080/00273171.2010.498292
77. Hayes AF, Scharkow M. The relative trustworthiness of inferential tests of the indirect effect in statistical mediation analysis: does method really matter? *Psychol Sci*. 2013;24(10):1918-1927. doi:10.1177/0956797613480187
78. Bradley EH, Curry LA, Devers KJ. Qualitative data analysis for health services research: developing taxonomy, themes, and theory. *Health Serv Res*. 2007;42(4):1758-1772. doi:10.1111/j.1475-6773.2006.00684.x
79. Palinkas LA, Aarons GA, Horwitz S, Chamberlain P, Hurlburt M, Landsverk J. Mixed method designs in implementation research. *Adm Policy Ment Health*. 2011;38(1):44-53. doi:10.1007/s10488-010-0314-z
80. Creswell JW, Klassen AC, Clark VLP, Smith KC. Best practices for mixed methods research in the health sciences. National Institutes of Health Office of Behavioral and Social Sciences. https://obssr.od.nih.gov/wp-content/uploads/2016/02/Best_Practices_for_Mixed_Methods_Research.pdf. Accessed October 1, 2019.
81. Beidas RS, Wolk CL, Walsh LM, Evans AC, Hurford MO, Barg FK. A complementary marriage of perspectives: understanding organizational social context using mixed methods. *Implement Sci*. 2014;9. doi:10.1186/s13012-014-0175-z
82. Locke J, Beidas RS, Marcus SC, et al. A mixed methods study of individual and organizational factors that affect implementation of interventions for children with autism in public schools. *Implement Sci*. 2016;11. doi:10.1186/s13012-016-0501-8
83. Ditty MS, Landes SJ, Doyle A, Beidas RS. It takes a village: a mixed method analysis of inner setting variables and dialectical behavior therapy implementation. *Adm Policy Ment Health*. 2015;42(6):672-681. doi:10.1007/s10488-014-0602-0
84. Wolk CB, Jager-Hyman S, Marcus SC, et al. Developing implementation strategies for firearm safety promotion in paediatric primary care for suicide prevention in two large US health systems: a study protocol for a mixed-methods implementation study. *BMJ Open*. 2017;7(6):e014407. doi:10.1136/bmjopen-2016-014407
