# Supplementary material for: Implementation of a Secure Firearm Storage Program in Pediatric Primary Care: A Cluster Randomized Trial
Source: JAMA Pediatr. 2024 Sep 3;178(11):1104–13. doi: 10.1001/jamapediatrics.2024.3274 (PMC11372656; doi:10.1001/jamapediatrics.2024.3274)
Supplement: Supplement 2. — eResults eFigure. Approximation of EHR nudges at the two health systems (i.e., not exact replicas or copies) [file jamapediatr-e243274-s002.pdf]

Supplemental Online Content

Beidas RS, Linn KA, Boggs JM, et al. Implementation of a secure firearm storage program in pediatric primary care: a cluster randomized trial. *JAMA Pediatrics*. Published online September 3, 2024. doi:10.1001/jamapediatrics.2024.3274

eResults
eFigure. Approximation of EHR nudges at the two health systems (i.e., not exact replicas or copies)

This supplemental material has been provided by the authors to give readers additional information about their work.

© 2024 Beidas RS et al. *JAMA Pediatrics*.

eResults

Additional Results: Unadjusted Models

Unadjusted models were fit using Generalized Estimating Equations (GEE) with a binomial distribution, logit link, and exchangeable working correlation at the clinic level. An indicator of condition (Nudge/Nudge+) was the only variable included in the model. We used the Kauermann and Carroll small cluster correction and fit the models using the *xtgeebcv* procedure and *margins* command in Stata Version 18.

Reach:
Based on the unadjusted model with reach as the outcome, the marginal probability of reach was 0.22 [95% CI, 0.15 – 0.30] under the Nudge condition and 0.50 [95% CI, 0.40 – 0.60] under Nudge+. The risk difference was 0.27 [95% CI, 0.15 – 0.40]. The risk difference point estimate and its corresponding 95% CI are similar to those based on the adjusted model, suggesting the results are robust to potential confounding by the variables we included in the adjusted model.

Counseling:
Based on the unadjusted model, the marginal probability of receiving the counseling component was 0.42 [97.5% CI, 0.31 – 0.53] under the Nudge condition and 0.62 [97.5% CI, 0.52 – 0.72] under Nudge+. The risk difference was 0.2 [97.5% CI, 0.05 – 0.35]. These point estimates and intervals were similar to those obtained from the adjusted model for counseling.

Lock:
Based on the unadjusted model, the marginal probability of receiving the lock component was 0.23 [97.5% CI, 0.14 – 0.31] under the Nudge condition and 0.50 [97.5% CI, 0.39 – 0.62] under Nudge+. The risk difference was 0.28 [97.5% CI, 0.13 – 0.42]. These point estimates and intervals were similar to those obtained from the adjusted model for locks.

Additional Results: Models Adjusting for Age, Sex, Ethnicity, and Race

Here we provide results from Generalized Estimating Equations (GEE) with a binomial distribution, logit link, and exchangeable working correlation at the clinic level that adjust for the following patient-level variables: age, sex, ethnicity (indicator for Hispanic ethnicity) and race (4 categories: White, Black, Other, Unknown; Other consisted of: Asian, American Indian / Alaskan Native, Hawaiian / Other Pacific Islander, other, more than one race). An indicator of condition (Nudge/Nudge+) was also included in the model. We used the Kauermann and Carroll small cluster correction and fit the models using the *xtgeebcv* procedure and *margins* command in Stata Version 18.

Reach:
The marginal probability of reach was 0.22 [95% CI, 0.09 – 0.34] under the Nudge condition and 0.51 [95% CI, 0.41 – 0.61] under Nudge+. The risk difference was 0.29 [95% CI, 0.12 – 0.46]. The risk difference point estimate and its corresponding 95% CI are similar to those based on the primary and unadjusted models, suggesting the results are robust to potential confounding by patient-level variables. Age was the only significant term in the reach model, with older age associated with lower reach (odds ratio 0.97 with 95% CI, 0.95 – 0.98).

Counseling:
The marginal probability of counseling was 0.42 [97.5% CI, 0.31 – 0.54] under the Nudge condition and 0.62 [97.5% CI, 0.51 – 0.73] under Nudge+. The risk difference was 0.20 [97.5% CI, 0.05 – 0.35]. Age was not significant in the counseling model, nor were any other patient-level covariates.

Lock:
The marginal probability of lock was 0.22 [97.5% CI, 0.08 – 0.36] under the Nudge condition and 0.51 [97.5% CI, 0.40 – 0.63] under Nudge+. The risk difference was 0.29 [97.5% CI, 0.10 – 0.48]. Age was the only significant term in the lock model, with older age associated with fewer lock offers (odds ratio 0.97 with 97.5% CI, 0.95 – 0.99).

© 2024 Beidas RS et al. *JAMA Pediatrics.*

eFigure. Approximation of EHR nudges at the two health systems (i.e., not exact replicas or copies)

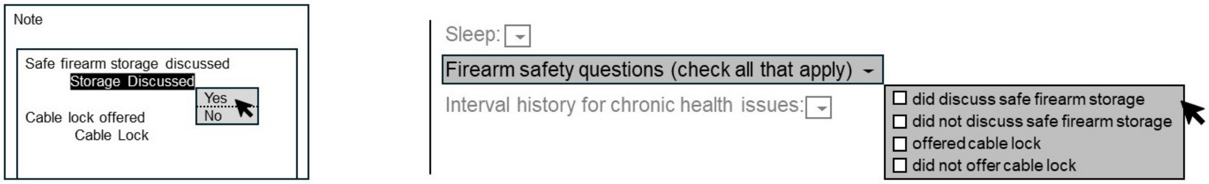

© 2024 Beidas RS et al. *JAMA Pediatrics*.
