# Supplementary material for: Implementation of a Secure Firearm Storage Program in Pediatric Primary Care: A Cluster Randomized Trial
Source: JAMA Pediatr. 2024 Sep 3;178(11):1104–13. doi: 10.1001/jamapediatrics.2024.3274 (PMC11372656; doi:10.1001/jamapediatrics.2024.3274)
Supplement: Supplement 3. — Data sharing statement [file jamapediatr-e243274-s003.pdf]

Data Sharing Statement

Beidas. Implementation of a Secure Firearm Storage Program in Pediatric Primary Care. *JAMA Pediatr*. Published September 03, 2024. doi:10.1001/jamapediatrics.2024.3274

Data
Data available: Yes
Data types: Deidentified participant data, Data dictionary
How to access data: The data analyzed in this paper can be access in the NIMH Data Archive at https://nda.nih.gov/data_structure.html?short_name=aspire_ehr01.
When available: With publication

Supporting Documents
Document types: None

Additional Information
Who can access the data: Those who meet NIMH Data Archive (NDA) data access requirements and receive NDA Data Access Committee approval.
Types of analyses: Analyses approved by the NDA Data Access Committee.
Mechanisms of data availability: NDA Data Access Committee approval.
Any additional restrictions: NA
